# NiPS_3_ ultrathin nanosheets as versatile platform advancing highly active photocatalytic H_2_ production

**DOI:** 10.1038/s41467-022-32256-6

**Published:** 2022-08-06

**Authors:** Jingrun Ran, Hongping Zhang, Sijia Fu, Mietek Jaroniec, Jieqiong Shan, Bingquan Xia, Yang Qu, Jiangtao Qu, Shuangming Chen, Li Song, Julie M. Cairney, Liqiang Jing, Shi-Zhang Qiao

**Affiliations:** 1 School of Chemical Engineering and Advanced Materials, University of Adelaide, Adelaide, SA 5005 Australia; 2 State Key Laboratory of Environmentally Friendly Energy Materials, Engineering Research Center of Biomass Materials (Ministry of Education), School of Materials Science and Engineering, Southwest University of Science and Technology, 621010 Mianyang, Sichuan China; 3 Department of Chemistry and Biochemistry & Advanced Materials and Liquid Crystal Institute, Kent State University, Kent, OH 44242 USA; 4 Key Laboratory of Functional Inorganic Material Chemistry (Ministry of Education), School of Chemistry and Materials Science, International Joint Research Center for Catalytic Technology, Heilongjiang University, 150080 Harbin, P. R. China; 5 Australian Centre for Microscopy and Microanalysis, The University of Sydney, Sydney, NSW 2006 Australia; 6 National Synchrotron Radiation Laboratory, CAS Center for Excellence in Nanoscience, University of Science and Technology of China, 230029 Hefei, Anhui P. R. China; 7 School of Physics, The University of Sydney, Sydney, NSW 2006 Australia

**Keywords:** Artificial photosynthesis, Photocatalysis, Two-dimensional materials

## Abstract

High-performance and low-cost photocatalysts play the key role in achieving the large-scale solar hydrogen production. In this work, we report a liquid-exfoliation approach to prepare NiPS_3_ ultrathin nanosheets as a versatile platform to greatly improve the light-induced hydrogen production on various photocatalysts, including TiO_2_, CdS, In_2_ZnS_4_ and C_3_N_4_. The superb visible-light-induced hydrogen production rate (13,600 μmol h^−1^ g^−1^) is achieved on NiPS_3_/CdS hetero-junction with the highest improvement factor (~1,667%) compared with that of pure CdS. This significantly better performance is attributed to the strongly correlated NiPS_3_/CdS interface assuring efficient electron-hole dissociation/transport, as well as abundant atomic-level edge P/S sites and activated basal S sites on NiPS_3_ ultrathin nanosheets advancing hydrogen evolution. These findings are revealed by the state-of-art characterizations and theoretical computations. Our work for the first time demonstrates the great potential of metal phosphorous chalcogenide as a general platform to tremendously raise the performance of different photocatalysts.

## Abstract (web summary)

While light-driven water splitting offers a renewable means to produce fuel, the limited availability of high-performance materials inspires the search for new photocatalysts. Here, authors demonstrate two-dimensional NiPS_3_ to enhance semiconductor photocatalytic H_2_ evolution activities.

## Introduction

The enormous consumption of non-renewable fossil fuels has led to the global energy shortage, environmental pollution and climate change. Therefore, seeking renewable, clean and carbon-free energy sources is of paramount importance. Solar hydrogen (H_2_) production via photocatalytic water splitting is considered a promising, inexpensive and environmentally benign technique to generate green H_2_ fuel using sunlight^1–36^. However, the large-scale application of this photocatalytic process is severely restricted by the low efficiency, poor stability and the high price of photocatalysts developed to date. Hence, seeking highly-active, robust and cheap photocatalysts is of great significance for realizing industrial-scale solar H_2_ generation^1–36^. Rational design and preparation of high-performance photocatalysts require not only the atomic-level understanding of the structure/composition–activity relationship^9,10,23,37–42^ but also the precise and insightful apprehension of the kinetics and thermodynamics of photo-generated electrons and holes in photocatalysts^5,12,43–49^ Merging the atomic-resolution aberration-corrected scanning transmission electron microscopy (AC-STEM) and theoretical computations could provide the atomic-level knowledge about the structure/composition-activity correlation for photocatalysts^9,10,23,37–42^. Particularly, various atomic-level reactive sites, e.g., single atoms^9,10,23,37,42^, edge sites^41^ and defects^38–40,42^, present in photocatalysts can be accurately revealed by the aforementioned approach. On the other hand, the separation/migration of photogenerated electrons and holes serve a pivotal role in determining the overall photocatalytic performance^1–36,43–49^. Thus, it is essential to adopt various advanced characterizations, e.g., ultrafast transient absorption spectroscopy (TAS), transient-state surface photovoltage (SPV) spectroscopy, transient-state photoluminescence (PL) spectroscopy and in situ X-ray photoelectron spectroscopy (XPS), for time-resolved study on the kinetics and thermodynamics of photogenerated electrons/holes in the bulk and, especially, on the surface of photocatalysts. Furthermore, it is of great importance to combine the above two strategies for simultaneous assessment of both the atomic-level structure/composition-performance relationship and the time-resolved charge-carrier separation/transfer mechanism for photocatalysts.

Recently, two-dimensional (2D) transitional metal phosphorous chalcogenides (MPC_*x*_) (M = Cr, Mn, Fe, Co, Ni, Zn, Ga, Cd, Sn and Bi; C = S, Se and Te) have attracted increasing attention in catalysis^50–62^, (opto)electronics^63,64^ and sensing^65^ owing to the distinct physicochemical properties. Nevertheless, this large group of materials is rarely applied in photocatalysis. This group of materials, due to their unique properties, is regarded as a potentially excellent platform for enhancing photocatalysis, which is ascribed to the following features: (1) ultrathin thickness favouring the dissociation of photogenerated electrons/holes, and transport to the surface; (2) large surface area facilitating the formation of strong interfacial electronic coupling with other materials; (3) abundant surface reactive sites promoting the redox catalytic reactions on the surface, and (4) thickness-dependent band gap width benefiting the flexible adjustment of electronic band structures for balancing light absorption and redox abilities of electrons/holes. To date, several experimental^50–56^ and computational^61,62^ works on 2D MPC_*x*_ materials in photocatalysis have been reported. For instance, Wang et al.^52^ reported that 2D NiPS_3_ nanosheets achieved the photocatalytic H_2_-production rates of ~26.4 and 74.67 μmol h^−1^ g^−1^ from pure water and Na_2_S/Na_2_SO_3_ aqueous solution, respectively, under xenon light irradiation. Additionally, Barua et al.^53^ reported the photocatalytic H_2_-production activity of 2600 μmol h^−1^ g^−1^ on Eosin Y-sensitized NiPS_3_ nanosheets in triethanolamine aqueous solution using a xenon lamp. Furthermore, FePS_3_ quantum sheets were synthesized to attain the photocatalytic H_2_-production rate of 290 μmol h^−1^ g^−1^ in triethanolamine aqueous solution using xenon light^51^. Moreover, theoretical computations were conducted to calculate the band gaps and band edge positions of MPS_3_ (M = Fe, Mn, Ni, Cd and Zn) and MPSe_3_ (M = Fe and Mn) monolayers^62^. These MPS_3_/MPSe_3_ monolayers were found to be good candidates for photocatalytic water splitting. Nevertheless, the application of MPC_*x*_ family as a general platform to greatly enhance the light-induced H_2_-production performance on various semiconductor photocatalysts, e.g., metal oxides, metal sulfides and metal-free nitrides, is not reported to date.

Here for the first time, we report a new liquid-exfoliation approach to acquire 2D MPC_*x*,_ NiPS_3_ ultrathin nanosheets (UNSs), as a versatile platform to apparently enhance the photocatalytic H_2_-production rates of metal oxide (TiO_2_), metal sulfides (CdS and In_2_ZnS_4_), and metal-free nitride (C_3_N_4_), respectively. Among them, the NiPS_3_/CdS heterojunction exhibits the largest photocatalytic H_2_-production rate of 13,600 μmol h^−1^ g^−1^ with the highest enhancement factor of ~1667% compared to CdS alone. The increased photocatalytic H_2_ production arises from the intimate electronic coupling promoting the interfacial charge separation/migration and the abundant atomic-level P/S edge sites together with activated S basal sites of NiPS_3_ boosting H_2_ evolution reaction. These findings are supported by both theoretical computations and state-of-art characterizations, which include atomic-resolution AC-STEM, electron energy loss (EELS) spectroscopy, synchrotron-based X-ray absorption near edge spectroscopy (XANES), in situ XPS, transient-state SPV spectroscopy, ultrafast TAS, transient-state PL spectroscopy and light-irradiated contact potential difference (CPD) test. This study further confirms the generality of NiPS_3_ UNSs in conjunction with the other semiconductor photocatalysts, e.g., TiO_2_, In_2_ZnS_4_ and C_3_N_4_, toward elevated photocatalytic H_2_ production. Our work not only demonstrates the great potential of this large MPC_*x*_ group in the photocatalysis field but more importantly, paves avenues for the rational design and preparation of high-performance photocatalysts via merging the advanced characterizations and theoretical calculations.

## Results

### Theoretical prediction, synthesis, characterization and application of 2D NiPS_3_

**Fig. 1:**
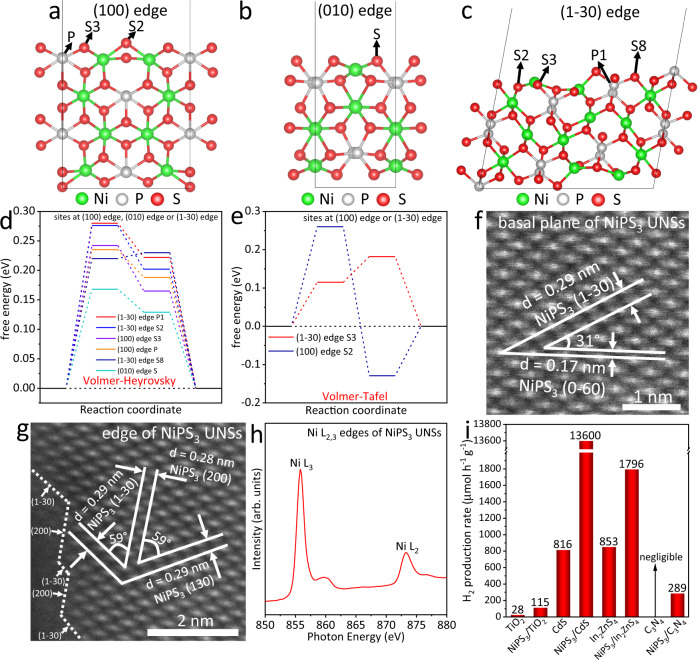
Theoretical prediction, characterization and application of NiPS_3_ UNSs. **a** Active P, S2 and S3 sites for HER at (100) edge of NiPS_3_ monolayer. **b** Active S site for HER at (010) edge of NiPS_3_ monolayer. **c** Active P1, S2, S3 and S8 sites for HER at (1–30) edge of NiPS_3_ monolayer. **d** Gibbs free energy diagrams for HER following the Volmer–Heyrovsky pathway on the active sites at (100) edge, (010) edge or (1−30) edge of NiPS_3_ monolayer. **e** Gibbs free energy diagrams for HER following the Volmer–Tafel pathway on the active sites at (100) or (1−30) edge of NiPS_3_ monolayer. Atomic-resolution HAADF-STEM images for **f** basal plane and **g** edge of NiPS_3_ UNSs. Synchrotron-based XANES **h** Ni L_2,3_ edges of NiPS_3_ UNSs. **i** Photocatalytic H_2_-production rates for TiO_2_, NiPS_3_/TiO_2_, CdS, NiPS_3_/CdS, In_2_ZnS_4_, NiPS_3_/In_2_ZnS_4_, C_3_N_4_ and NiPS_3_/C_3_N_4_ in ~17.0 vol% triethanolamine aqueous solution. Among them, TiO_2_ and NiPS_3_/TiO_2_ were excited by xenon light without cut-off filter. CdS, NiPS_3_/CdS, In_2_ZnS_4_, NiPS_3_/In_2_ZnS_4_, C_3_N_4_ and NiPS_3_/C_3_N_4_ were excited by visible-light irradiation (*λ* > 400 nm). All the Gibbs free energies were calculated considering the solvation effect in 17 vol% triethanolamine aqueous solution.

We selected NiPS_3_ from a series of transitional metal phosphorous chalcogenides (MPC_*x*_) (M = Cr, Mn, Fe, Co, Ni, Zn, Ga, Cd, Sn and Bi; C = S, Se and Te) and predicted the potential properties via density functional theory (DFT) based computations. We made this selection based on the previous experimental results as summarized in Supplementary Table 1. As shown in Supplementary Table 1, among all the reported MPS_*x*_ and MPSe_x_, NiPS_3_ shows the lowest overpotential (193 mV) for electrochemical hydrogen evolution reaction (HER) in the alkaline solution, as the current density reaches −10 mA cm^−2^. Besides, considering that MPTe_*x*_ is rarely reported for photocatalytic/electrocatalytic hydrogen (H_2_) evolution, we decide to select NiPS_3_ and study its HER ability using DFT-based calculations. In this work, we focus on the HER activity to explore whether NiPS_3_ can serve as a versatile platform promoting photocatalytic H_2_ production. Generally, a three-state diagram, which consists of an initial state H^+^ + *e*^−^, an intermediate adsorbed H*, and a final product ½H_2_, is utilized to summarize the whole HER process. The Gibbs free energy of the intermediate state, |Δ*G*_H*_|, is considered a major indicator for the HER activity on different types of catalysts. The most desirable value of |Δ*G*_H*_| is zero. For instance, the well-known HER catalyst with excellent activity, Pt, exhibits a near-zero value of Δ*G*_H*_ ≈ −0.09 eV. As a result, we apply DFT-based computations to calculate the Δ*G*_H*_ values for the sites at the basal plane and edge of NiPS_3_ monolayer. Twenty-four possible HER active sites on the basal plane (Supplementary Fig. 1a–c), (100) edge (Supplementary Fig. 1d–f), (010) edge (Supplementary Fig. 1g–i) and (1–30) edge (Supplementary Figs. 2a–f and 3a–i) of NiPS_3_ monolayer were studied to predict the most active sites for HER. Accordingly, the HER free energy diagrams via either the Volmer–Heyrovsky pathway (Supplementary Figs. 1b, e, h, 2b, e and 3b, e, h) or the Volmer–Tafel pathway (Supplementary Figs. 1c, f, i, 2c, f and 3c, f, i) were acquired to disclose the reaction mechanism. The corresponding Δ*G*_H*_ values via either Volmer–Heyrovsky or Volmer–Tafel pathway are displayed in Supplementary Tables 2–6. All the above theoretical calculations were performed considering the solvation effect in 17 vol% triethanolamine aqueous solution, in which the HER will occur. Among these 24 HER active sites, 8 most active sites for HER are displayed in Fig. 1a–c, which are P, S2 and S3 sites at (100) edge, S site at (010) edge together with P1, S2, S3 and S8 sites at (1–30) edge of NiPS_3_ monolayer. Furthermore, based on the Δ*G*_H*_ values of these 8 active sites (Supplementary Tables 3–6), the P and S3 sites at (100) edge, S site at (010) edge together with P1, S2 and S8 sites at (1–30) edge follow the Volmer-Heyrovsky pathway (Fig. 1d); while the S2 site at (100) edge and S3 site at (1–30) edge follow the Volmer-Tafel pathway (Fig. 1e). Among the other 16 sites of NiPS_3_ monolayer, S4, S5 and S7 sites at (1–30) edge show the small │Δ*G*_H*_│values for Volmer step owing to the edge effect (Supplementary Table 6). However, they are not deemed as effective active sites because of the high free energy change for the second step of H_2_ formation (Supplementary Table 6). Overall, the aforementioned DFT-based computations reveal the excellent HER activities on specific P and S edge sites of NiPS_3_ monolayer. In comparison, the basal plane sites and Ni edge sites of NiPS_3_ monolayer are not considered the active sites for HER. On the other hand, 2D NiPS_3_ also possesses the well-known advantages of ultrathin thickness and large surface area. These properties not only facilitate the efficient bulk-to-surface charge carrier migration but also enhance electronic interaction with other materials for rapid interfacial charge carrier transport and optimized catalytic activities.

Thus, we developed a new and facile liquid exfoliation procedure (see methods) to obtain the NiPS_3_ ultrathin nanosheets (UNSs) in ethanol. The transmission electron microscopy (TEM) image in Supplementary Fig. 4a shows NiPS_3_ UNSs with lateral sizes of ~216–263 nm. The high-resolution (HR)TEM image of NiPS_3_ UNSs (Supplementary Fig. 4b) shows the lattice spacing value of 0.17 nm, in accordance with the (0−60) facet of monoclinic NiPS_3_. The energy dispersive X-ray spectroscopy (EDX) pattern of NiPS_3_ UNSs (Supplementary Fig. 4c) shows the presence of Ni, P and S, in agreement with the TEM and HRTEM results. Moreover, the high-angle annular dark field scanning transmission electron microscopy (HAADF-STEM) image (Supplementary Fig. 5a) and the corresponding elemental mapping images of Ni (Supplementary Fig. 5b), S (Supplementary Fig. 5c) and P (Supplementary Fig. 5d) again confirm the successful preparation of NiPS_3_ UNSs. The atomic-resolution HAADF-STEM image (Fig. 1f) of the basal plane for NiPS_3_ UNSs shows two lattice spacing values of 0.29 and 0.17 nm, together with an angle of 31°. These two crystal planes, respectively, correspond to the (1−30) and (0−60) planes of monoclinic NiPS_3_. Additionally, three lattice spacing values of 0.29, 0.28 and 0.29 nm together with two angles of 59° and 59° are observed in Fig. 1g. These three crystal planes are assigned to the (1−30), (200) and (130) planes of monoclinic NiPS_3_, respectively. Furthermore, many steps along the (1−30) and (200) edges of NiPS_3_ UNSs can be observed in Fig. 1g, indicating the existence of many P and S active sites at the edges of NiPS_3_ UNSs. This implies the high activity of NiPS_3_ UNSs toward HER. The atomic force microscopy (AFM) image of NiPS_3_ UNSs (Supplementary Fig. 6a) shows the UNS morphology with lateral sizes of ~157–480 nm. The corresponding height profile of NiPS_3_ UNSs (Supplementary Fig. 6b) exhibits a thickness of ~3.16 nm, verifying the ultrathin thickness. Such a thickness (~3.16 nm) corresponds to 3 or 4 atomic layers of NiPS_3_ based on the single-layer thickness of ~0.8–1.1 nm^63^. The high-resolution XPS spectra of Ni *2p* (Supplementary Fig. 7a), P *2p* (Supplementary Fig. 7b) and S *2p* (Supplementary Fig. 7c) of NiPS_3_ UNSs are in accordance with those reported elsewhere^60^. Moreover, the synchrotron-based X-ray absorption near edge spectroscopy (XANES) Ni L_2,3_ edges for NiPS_3_ UNSs are displayed in Fig. 1h. The Raman spectrum of NiPS_3_ UNSs (Supplementary Fig. 8a) exhibits the presence of *E*^*(2)*^_*g*_, *A*^*(1)*^_*1g*_, *A*^*(2)*^_*1g*_ and *A*^*(3)*^_*1g*_ modes, in agreement with the prior report^60^. Moreover, the UV-Vis absorption spectrum of NiPS_3_ UNSs (Supplementary Fig. 8b) exhibits the absorption edge at 873 nm, suggesting the narrow band gap width (E) of 1.42 eV. The picture of NiPS_3_ UNSs ethanol solution (Supplementary Fig. 8b inset) shows the typical Tyndall effect, indicating the formation of a homogeneous dispersion of NiPS_3_ UNSs in ethanol.

To explore the potential application of the as-prepared NiPS_3_ UNSs in photocatalysis, we first investigate the detailed electronic band structure of NiPS_3_ UNSs. The flat band potential of NiPS_3_ UNSs is −0.58 V vs. Ag/AgCl electrode (Supplementary Fig. 9a). Thus, the Fermi level (*E*_F_) of NiPS_3_ UNSs is 0.05 V vs. standard hydrogen electrode (SHE). The XPS valence band (VB) spectrum of NiPS_3_ UNSs (Supplementary Fig. 9b) indicates that the VB edge potential of NiPS_3_ UNSs is 0.81 V vs. *E*_F_ of NiPS_3_ UNSs. Hence, the VB edge potential of NiPS_3_ UNSs is 0.86 V vs. SHE. As a result, the conduction band (CB) edge potential of NiPS_3_ UNSs is −0.56 V vs. SHE, based on the band gap of NiPS_3_ UNSs (*E* = 1.42 eV). The electronic band structure of NiPS_3_ UNSs is displayed in Supplementary Fig. 10. The CB edge potential of NiPS_3_ UNSs (−0.56 V vs. SHE) is much more negative than the HER potential (0.0 V vs. SHE). However, the VB edge potential of NiPS_3_ UNSs (0.86 V vs. SHE) is not sufficiently positive for the oxygen evolution reaction (1.23 V vs. SHE). These results reveal the strong reduction ability of photogenerated electrons and weak oxidation capacity of photogenerated holes in NiPS_3_ UNSs. On the other hand, the narrow band gap (*E* = 1.42 eV) of NiPS_3_ UNSs implies a wide light-responsive range (up to 873 nm) and excellent electronic conductivity. Nevertheless, the poor oxidation ability of photogenerated holes in NiPS_3_ UNSs renders it more suitable to be merged with other semiconductor photocatalysts supplying strongly oxidative photogenerated holes rather than being used alone.

Therefore, interface engineering was applied to combine NiPS_3_ UNSs with other semiconductor photocatalysts, which could establish strong interfacial electronic coupling to greatly facilitate the separation/migration of photogenerated electron-hole pairs and optimize the HER performance of the reactive sites on NiPS_3_ UNSs. In detail, the as-prepared 20.0 ml of NiPS_3_ UNSs ethanol solution was merged with 50 mg of four different semiconductor photocatalysts, respectively, which include metal oxide (TiO_2_), metal sulfides (CdS and In_2_ZnS_4_) and metal-free nitride (C_3_N_4_). The resulting hybrid photocatalysts were denoted, respectively, as NiPS_3_/TiO_2_, NiPS_3_/CdS, NiPS_3_/In_2_ZnS_4_ and NiPS_3_/C_3_N_4_. The photocatalytic H_2_-production rates of all the hybrid photocatalysts exhibit an apparent improvement in contrast to the photocatalysts without NiPS_3_ (Fig. 1i). Among them, NiPS_3_/CdS shows the highest photocatalytic H_2_-production rate (13,600 μmol h^−1^ g^−1^), surpassing that of bare CdS (816 μmol h^−1^ g^−1^) by ~1667%. These results show that NiPS_3_ UNSs could serve as a versatile and highly active platform to enhance the photocatalytic H_2_-production rates on various kinds of photocatalysts. Since the NiPS_3_/CdS system presents the highest photocatalytic H_2_-production rate (13,600 μmol h^−1^ g^−1^) and largest enhancement factor (~1667%), a range of experiments and theoretical computations were performed to investigate this representative NiPS_3_/CdS system.

### Phase structure, morphology and strong electronic interaction of NiPS_3_/CdS heterojunction

First, pure CdS NPs synthesized via a hydrothermal approach were studied using HAADF-STEM, elemental mapping, EDX spectroscopy and Raman spectroscopy. The HAADF-STEM image of CdS NPs (Supplementary Fig. 11a) shows the sizes of ~11–36 nm for CdS NPs. The high-resolution HAADF-STEM image of CdS NPs (Supplementary Fig. 11b) presents the two lattice spacing values of 0.34 and 0.34 nm, together with an angle of 70.5°, attributed, respectively, to the (111) and (11−1) facets of cubic CdS. Moreover, the HAADF-STEM image (Supplementary Fig. 11c) and corresponding elemental mapping images (Supplementary Fig. 11d, e) of CdS NPs further indicate the successful preparation of CdS NPs. The EDX spectrum of CdS NPs (Supplementary Fig. 11f) indicates the presence of Cd and S elements in agreement with the above results. The Raman spectrum of CdS NPs in Supplementary Fig. 12 confirms the presence of typical 1LO, 2LO and 3LO peaks for CdS in agreement with the previous work^66^.

Then, different volumes of NiPS_3_ UNSs ethanol solution (5.0, 10.0, 20.0 and 30.0 ml) were, respectively, added to a mortar to couple with 50 mg of the as-prepared CdS NPs via mechanical grinding at room temperature. The resulting photocatalysts were, respectively, labelled as 5.0N, 10.0N, 20.0N and 30.0N. Pure CdS NPs were denoted as 0.0N. First, the phase structures of all the as-prepared samples were revealed by X-ray diffraction (XRD). The XRD patterns of 0.0N, 5.0N, 10.0N, 20.0N and 30.0N are displayed in Supplementary Fig. 13. All the samples exhibit a Hawleyite cubic-phase structured CdS (JCPDS #10-0454). No apparent alteration of the intensities and positions of diffraction peaks are observed for 5.0N, 10.0N, 20.0N and 30.0N, in contrast with those of 0.0N (Supplementary Fig. 13). These results suggest that the physical mixing of CdS NPs with NiPS_3_ UNSs at room temperature does not impact the original crystal structure of CdS NPs. Moreover, the absence of NiPS_3_ diffraction peak in 5.0N, 10.0N, 20.0N and 30.0N arises from the low content and homogeneous dispersion of NiPS_3_ UNSs.

**Fig. 2:**
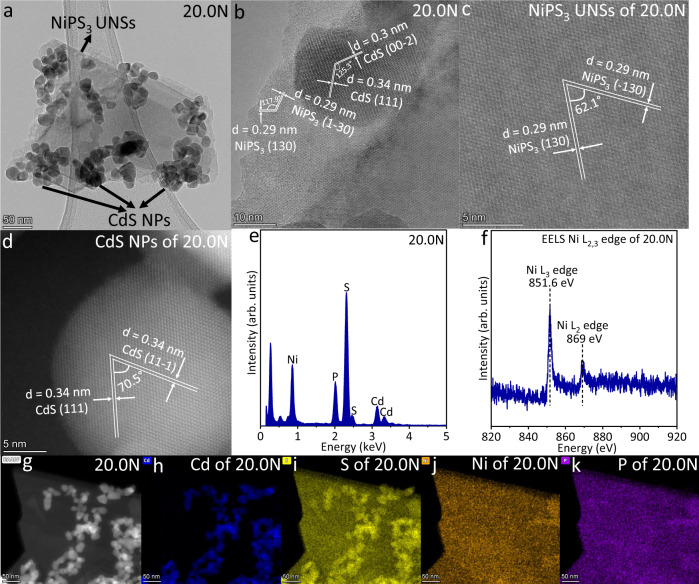
Morphology, microstructure, and chemical compositions of 20.0N. **a** TEM image and **b** HRTEM image of 20.0N. Atomic-resolution HAADF-STEM images of **c** NiPS_3_ UNSs and **d** CdS NPs in 20.0N. **e** EDX spectrum of 20.0N. **f** EELS spectrum of Ni L_2,3_ edge for 20.0N. **g** HAADF-STEM image of 20.0N and the corresponding elemental mapping images of **h** Cd, **i** S, **j** Ni and **k** P elements in 20.0N.

**Fig. 3:**
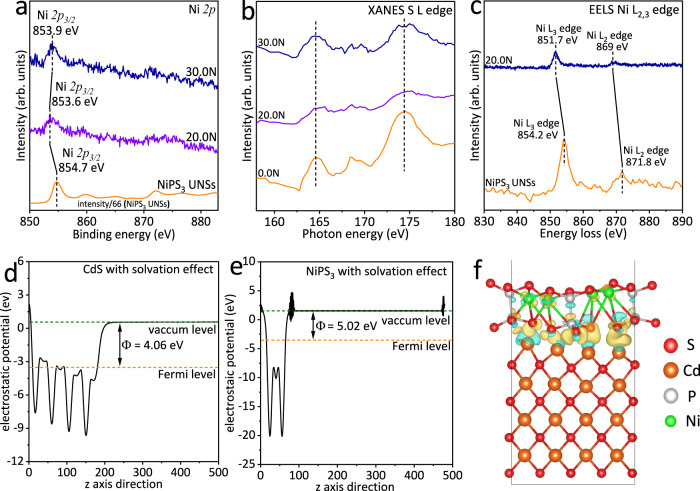
Strong electronic interaction in NiPS_3_/CdS system. **a** High-resolution XPS spectra of Ni *2p* for NiPS_3_ UNSs, 20.0N and 30.0N. **b** Synchrotron-based XANES S L edge of 0.0N, 20.0N and 30.0N. **c** EELS spectra of Ni L_2,3_ edge for NiPS_3_ UNSs and 20.0N. Average potential profiles along the *z* axis direction of **d** CdS (200) crystal facet and **e** NiPS_3_ (002) crystal facet. **f** The differential charge density map of NiPS_3_/CdS system. The golden and cyan iso-surfaces indicate the area of net-electron accumulation and deficit, respectively. The work functions and differential charge density map were calculated considering the solvation effect in 17 vol% triethanolamine aqueous solution.

Then, the morphology, microstructure and compositions of 20.0N were explored. The TEM image of 20.0N is shown in Fig. 2a. CdS NPs with sizes of ~15–39 nm are uniformly dispersed onto the surface of NiPS_3_ UNSs. The HRTEM image in Fig. 2b further shows two lattice spacing values of 0.30 and 0.34 nm, together with an angle of 125.3°. These two crystal planes are ascribed to the (00−2) and (111) facets of cubic CdS, respectively. Besides, the other two lattice spacing values of 0.29 and 0.29 nm, and an angle of 117.9° are also displayed in Fig. 2b. These two crystal planes are, respectively, assigned to the (130) and (1−30) planes of monoclinic NiPS_3_. The above results further confirm the successful loading of CdS NPs onto NiPS_3_ UNSs. The atomic-resolution HAADF-STEM image of NiPS_3_ UNSs in 20.0N (Fig. 2c) displays two lattice spacing values of 0.29 and 0.29 nm, accompanied by an angle of 62.1°. These two facets are ascribed to the (130) and (−130) planes of monoclinic NiPS_3_ UNSs, respectively. Figure 2d exhibits the atomic-resolution HAADF-STEM image of CdS NPs in 20.0N. Two lattice spacing values of 0.34 and 0.34 nm, as well as an angle of 70.5° are observed in Fig. 2d, corresponding to (111) and (11−1) facets of cubic CdS. The EDX spectrum of 20.0N (Fig. 2e) suggests the existence of Cd, S, Ni and P elements, in accordance with the above results. The electron energy loss spectroscopy (EELS) pattern of Ni L_2,3_ edges for 20.0N (Fig. 2f) shows that the Ni L_2_ edge and Ni L_3_ edge are located at 869 and 851.6 eV, respectively. Moreover, the HAADF-STEM image of 20.0N (Fig. 2g) further shows that many CdS NPs are loaded onto the surface of NiPS_3_ UNSs. The much brighter CdS NPs than NiPS_3_ NSs in Fig. 2g arises from the much larger atomic number of Cd (Z = 48) compared with those of Ni (Z = 28), P (Z = 15) and S (Z = 16). The corresponding elemental mapping images of Cd (Fig. 2h), S (Fig. 2i), Ni (Fig. 2j) and P (Fig. 2k) are in accordance with the HAADF-STEM image of 20.0N (Fig. 2g). All the above results support the successful merging of CdS NPs with NiPS_3_ UNSs in 20.0N. XPS analysis, which can reveal the surface element status, was adopted to detect the interfacial electronic interaction between CdS NPs and NiPS_3_ UNSs. As shown in Fig. 3a, the Ni *2p*_*3/2*_ peaks of 20.0N and 30.0N exhibit the obvious left shift toward the low binding energy direction, in contrast with that of NiPS_3_ UNSs. These results disclose the electron transfer from CdS NPs to NiPS_3_ UNSs after combining CdS NPs and NiPS_3_ UNSs in 20.0N and 30.0N. Notably, in comparison with the Ni *2p*_*3/2*_ peak of NiPS_3_ UNSs, the Ni *2p*_*3/2*_ peak of 20.0N shows a left shift of 1.1 eV; while the Ni *2p*_*3/2*_ peak of 30.0N exhibit a smaller left shift of 0.8 eV (Fig. 3a). This is attributed to the higher amount of NiPS_3_ UNSs in 30.0N than that in 20.0N. Moreover, compared to the Cd *3d* and S *2p* peaks of 0.0N (CdS NPs) in Supplementary Fig. 14a, b, no obvious shift of Cd *3d* and S *2p* peaks is observed in 20.0N and 30.0N, which is due to the following two reasons: (1) the weight amount of CdS NPs is much larger than that of NiPS_3_ UNSs in 20.0N or 30.0N. This is corroborated by the inductively coupled plasma atomic emission spectroscopy (ICP-AES) test, displaying the weight amount of NiPS_3_ UNSs (3.603 wt%) in 20.0N (Supplementary Table 7); (2) some CdS NPs tend to aggregate rather than contact with NiPS_3_ UNSs in 20.0N and 30.0N. In addition, the synchrotron-based X-ray absorption near edge structure (XANES) S L edges of 20.0N and 30.0N exhibit no obvious shift in contrast with that of 0.0N (Fig. 3b), which agrees with the aforementioned XPS results. Furthermore, the EELS Ni L_2,3_ edges for 20.0N show the apparent shift (2.5 or 2.8 eV) toward the low energy direction, in contrast with those of NiPS_3_ UNSs (Fig. 3c). This obvious shift is because the EELS Ni L_2,3_ edge spectrum was acquired in the selected region, where CdS NPs contact with NiPS_3_ UNSs. Hence, the EELS spectra (Fig. 3c) also corroborate the electron migration from CdS NPs to NiPS_3_ UNSs. Moreover, the Mott-Schottky plots in Supplementary Figs. 15 and 9a show that the flat band potentials of CdS NPs and NiPS_3_ UNSs are -0.89 and -0.58 V vs. Ag/AgCl electrode, corresponding to the −0.26 and 0.05 V vs. SHE. Thus, the E_F_ value for CdS NPs is more negative than that of NiPS_3_ UNSs. As a result, electrons would migrate from CdS NPs to NiPS_3_ UNSs via the interface in 20.0N. Furthermore, as shown in Fig. 3d, e, the work functions (Φ) of CdS (200) crystal facet and NiPS_3_ (002) crystal facet are, respectively, calculated to be 4.06 and 5.02 eV. These results also support the electron extraction from CdS to NiPS_3_ at the interface after combining CdS and NiPS_3_. Besides, the differential charge density map at the interface of CdS and NiPS_3_ (Fig. 3f) also suggests the interfacial electron transport from CdS to NiPS_3_ after coupling them. The Bader charge transferred from CdS to NiPS_3_ is │1.34│e, also in support of the electron migration from CdS to NiPS_3_. Moreover, the spin-up and spin-down electronic band structures and density of states (DOS) for CdS and NiPS_3_ were calculated, respectively. As displayed in Supplementary Fig. 16a, b, both the conduction band minimum (CBM) and valence band maximum (VBM) of CdS are located at the X point of the Brillouin zone with the direct band gap transition. The calculated direct (ΔE) band gap between CBM and VBM of CdS is 1.90 eV, which is close to the experimental value^13^. The spin-up and spin-down DOS of CdS (Supplementary Fig. 16c) also show the same band gap (Δ*E* = 1.90 eV), in accordance with the calculated band structures (Supplementary Fig. 16a, b). Moreover, NiPS_3_ shows the spin-polarized electronic band structures with a direct spin-up band gap of 1.75 eV (Supplementary Fig. 16d) and indirect spin-down band gap of 1.83 eV (Supplementary Fig. 16e), in agreement with the calculated and experimental results^52,57^. And the spin-up and spin-down DOS of NiPS_3_ (Supplementary Fig. 16f) display an identical band gap (Δ*E* = 1.75 eV), in agreement with the calculated band structures (Supplementary Fig. 16d, e).

The presence of strong electronic interaction at the interface between CdS and NiPS_3_ is confirmed by both the above experimental results and theoretical calculations. To further corroborate that NiPS_3_ UNSs can establish a strong electronic coupling between various semiconductor photocatalysts, we have conducted a range of high-resolution XPS studies for NiPS_3_/TiO_2_ (Supplementary Fig. 17a–d), NiPS_3_/In_2_ZnS_4_ (Supplementary Fig. 17e–h) and NiPS_3_/C_3_N_4_ (Supplementary Fig. 17i–l). All the results confirm the electrons migration from TiO_2_, In_2_ZnS_4_ or C_3_N_4_ to NiPS_3_ UNSs, as evidenced by the pronounced shift of high-resolution XPS peaks (Supplementary Fig. 17a–l).

### Excellent photocatalytic performance of NiPS_3_/CdS heterojunction

**Fig. 4:**
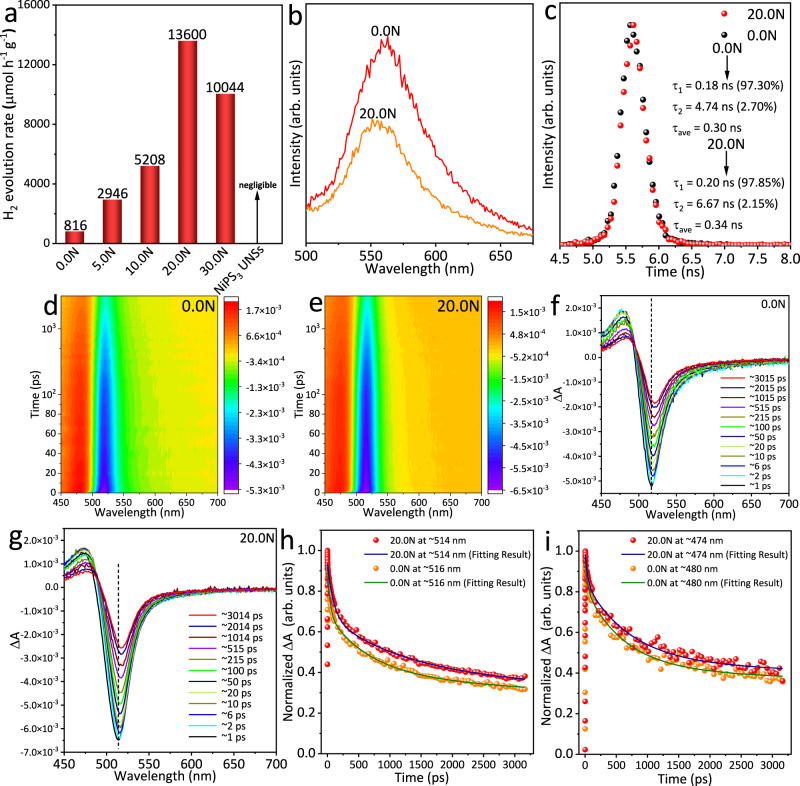
Photocatalytic H_2_-production activities and charge carrier kinetics of NiPS_3_/CdS system. **a** Photocatalytic H_2_-production rates of 0.0N, 5.0N, 10.0N, 20.0N, 30.0N and NiPS_3_ UNSs in ~17.0 vol% triethanolamine aqueous solution using visible-light illumination (*λ* > 400 nm). **b** Steady-state and **c** transient-state PL spectra of 0.0N and 20.0N. **c** Inset shows the fitting charge lifetimes for 0.0N and 20.0N. 2D pseudocolor TA spectra of **d** 0.0N and **e** 20.0N in ethanol solution after the excitation with a 400 nm laser pulse. The TA spectra of **f** 0.0N and **g** 20.0N at different pump-probe delay time. **h** Normalized decay kinetics and fitting lines for 0.0N and 20.0N taken through the GSB peaks at ~516 and ~514 nm, respectively. **i** Normalized decay kinetics and fitting lines for 0.0N and 20.0N taken through the ESA peaks at ~480 and ~474 nm, respectively.

The photocatalytic H_2_-production rates of all the as-synthesized samples were examined in ~17 vol% triethanolamine aqueous solution with visible-light illumination (*λ* > 400 nm). 0.0N (pure CdS NPs) presents a low photocatalytic H_2_-production rate of 816 μmol h^−1^ g^−1^ (Fig. 4a) owing to the fast recombination of photoinduced electron-hole pairs and insufficient active sites on the surface. After combining CdS NPs with NiPS_3_ UNSs, 5.0N exhibits an obviously-increased photocatalytic H_2_-production rate (2946 μmol h^−1^ g^−1^). Further increase in the NiPS_3_ UNSs amount results in an even larger photocatalytic H_2_-production rate of 5208 μmol h^−1^ g^−1^ on 10.0N. Remarkably, 20.0N exhibits the largest photocatalytic H_2_-production rate (13,600 μmol h^−1^ g^−1^). This excellent activity is ranked among the most efficient noble-metal-free CdS-based photocatalysts (Supplementary Table 8). Besides, this activity is also higher than the other MPC_*x*_-based photocatalysts (Supplementary Table 9)^50–55^, such as Eosin Y-sensitized NiPS_3_ sheets (2600 μmol h^−1^ g^−1^)^53^, NiPS_3_ nanosheets covered carbon fibre (74.67 μmol h^−1^ g^−1^)^52^, FePS_3_ nanosheets (402.4 μmol h^−1^ g^−1^)^55^ and MnPSe_3_ nanosheets covered carbon fibre (43.5 μmol h^−1^ g^−1^)^50^. These results also support that NiPS_3_ is better to be combined with another photocatalyst rather than being used alone for achieving a further raise in photocatalytic H_2_-production activity. Additionally, 20.0N shows an apparent quantum yield (AQY) of 20.2% at 420 nm, ranking as one of the most active noble-metal-free CdS-based photocatalysts (Supplementary Table 8). Nevertheless, we have also noticed that some works reported higher photocatalytic H_2_-production rates and AQYs as displayed in Supplementary Table 8. For instance, MoS_2_/CdS heterostructure shows a photocatalytic H_2_-production rate of 49,800 μmol h^−1^ g^−1^ in lactic acid aqueous solution under visible-light illumination^14^. Besides, this heterostructure also displays an AQY of 41.37% at 420 nm. In another work, WO_3_/CdS/WS_2_ direct *Z*-scheme heterojunction exhibits a photocatalytic H_2_-production rate of 14,340 μmol h^−1^ g^−1^ in lactic acid aqueous solution with visible light irradiation^36^. And an AQY of 22.96% at 435 nm was also achieved on this heterojunction. However, the above rates and AQYs were acquired in the photocatalytic test systems with different reaction conditions, such as weight of catalyst, pH value of reaction solution and type/concentration of sacrificial reagent. Apart from the intrinsic activities of photocatalysts, these factors can also affect the above rates and AQYs^33–35^. On the other hand, the Mo and W elements in MoS_2_ and WS_2_, respectively, possess a much lower abundance (1.20 and 1.25 ppm for Mo and W, respectively) than that of Ni element (84.00 ppm) in Earth’s crust^67^. Thus, the NiPS_3_ UNSs developed in our work are a more cost-effective platform for advancing photocatalytic H_2_ production, compared with MoS_2_ or WS_2_. Notably, a further increase in the NiPS_3_ UNSs amount results in a decrease in the photocatalytic H_2_-production rate to 10,044 μmol h^−1^ g^−1^ on 30.0N, possibly due to the excessive NiPS_3_ UNSs partially blocking light harvesting and covering surface active sites. We also tested the photocatalytic H_2_-production rate of pure NiPS_3_ UNSs. However, no H_2_ production was observed on pure NiPS_3_ UNSs under the same reaction conditions, probably due to the weak oxidation abilities of photogenerated holes in the VB of NiPS_3_ UNSs (Supplementary Fig. 10). Thus, the photogenerated holes cannot oxidize the sacrificial electron donor, triethanolamine, leading to the fast recombination of photo-generated electron-hole pairs.

The stability of 20.0N was also tested for four cycles. The H_2_-production amount in the fourth cycle accounts for 49.17% of that in the first hour (Supplementary Fig. 18). To find the reason for the reduced activity, we have conducted the XRD, TEM, EDX and atomic-resolution HAADF-STEM characterizations for 20.0N after the 4-h reaction (20.0N-A). As shown in Supplementary Fig. 19, there is a slight reduction in the peak intensities of the XRD peaks for 20.0N-A compared to those of 20.0N, suggesting the weakened crystallinity of CdS NPs after the reaction. However, the TEM image (Supplementary Fig. 20a) and EDX spectrum (Supplementary Fig. 20b) of 20.0N-A show that no obvious alteration in the morphology and chemical compositions is found for 20.0N-A in comparison to those of 20.0N (Fig. 2a, e). Thus, the atomic-resolution HAADF-STEM images of 20.0N-A were explored. We found that the amorphous region appears on the surface of CdS NPs (Supplementary Fig. 20c) and at the edge of NiPS_3_ UNSs (Supplementary Fig. 20d). It seems that the generation of an amorphous region on the surface of CdS NPs (Supplementary Fig. 20c) led to the reduced crystallinity of CdS NPs in 20.0N-A (Supplementary Fig. 19). Notably, since abundant H_2_-evolution active sites exist at the edges of NiPS_3_ UNSs, the structure destruction at the edges of NiPS_3_ UNSs resulted in the reduced H_2_-production activity of 20.0N-A (Supplementary Fig. 18). To further explore the stability of 20.0N, we have tested its stability in 0.35 M Na_2_S and 0.25 M Na_2_SO_3_ aqueous solution, which is usually applied as the sacrificial electron donor to inhibit the self-corrosion of metal sulfide photocatalysts. However, a similar reduction of activity in the second to the fourth hour is also observed in 0.35 M Na_2_S and 0.25 M Na_2_SO_3_ aqueous solution (Supplementary Fig. 21). And we have acquired the TEM image, EDX spectrum and atomic-resolution HAADF-STEM images of 20.0N after the four-hour reaction in Na_2_S and Na_2_SO_3_ aqueous solution (20.0N-A-S) as shown in Supplementary Fig. 22a–d. No apparent difference is observed in the morphology (Supplementary Fig. 22a) and chemical compositions (Supplementary Fig. 22b) of 20.0N-A-S, in comparison to those of 20.0N (Fig. 2a, e). However, the atomic-resolution HAADF-STEM images of 20.0N-A-S reveal the existence of an amorphous region on the surface of CdS NPs (Supplementary Fig. 22c) and at the edge of NiPS_3_ UNSs (Supplementary Fig. 22d), respectively. These are attributed to the photo-corrosion caused by the photogenerated holes in 20.0N, thus indicating that Na_2_S/Na_2_SO_3_, unfortunately, cannot effectively impede the photo-corrosion on CdS NPs and NiPS_3_ UNSs in this work.

### Kinetics and thermodynamics of photogenerated electrons/holes in NiPS_3_/CdS heterojunction

The origin of the excellent photocatalytic performance on 20.0N was investigated by both the advanced characterizations and theoretical computations. Since the interfacial charge separation/migration serves a pivotal role in the whole photocatalysis process, the dissociation and transport of photogenerated electrons/holes were investigated using a range of advanced characterizations, including steady-state and transient-state photoluminescence (PL) spectroscopy, ultrafast transient absorption spectroscopy (TAS), steady-state and transient-state surface photovoltage (SPV) spectroscopy, light-irradiated contact potential difference (CPD) test, in situ XPS, and transient photocurrent (TPC) density measurements.

To study the charge carrier separation and migration, we need to determine the heterojunction type of NiPS_3_/CdS system first. As shown in Supplementary Fig. 23a, the VB edge of 0.0N (CdS NPs) is 1.72 V vs. the *E*_F_ of 0.0N. Since the *E*_F_ of 0.0N is decided to be −0.26 V vs. SHE (Supplementary Fig. 15), the VB edge of 0.0N (CdS NPs) is 1.46 V vs. SHE. Thus, the CB edge of 0.0N (CdS NPs) is −0.78 V vs. SHE, based on the band gap value (*E* = 2.24 eV; Supplementary Fig. 23b). Therefore, as shown in Supplementary Fig. 24, a type I (straddling type) heterojunction is formed in the NiPS_3_/CdS system. However, type I heterojunction is usually not regarded as an effective heterostructure, since both photogenerated electrons and holes would migrate to the same semiconductor with a smaller band gap width. This would lead not only to the decreased redox abilities of electrons and holes but also to the inefficient dissociation of electrons and holes. But NiPS_3_/CdS system has achieved a very high photocatalytic H_2_-production rate (Fig. 4a). So, various state-of-art characterizations were performed to understand the kinetics and thermodynamics of photogenerated charge carriers in NiPS_3_/CdS system (20.0N). Both the steady-state and transient-state PL spectroscopy measurements were performed to study the radiative recombination of photo-generated electrons/holes in 20.0N. The steady-state PL spectra of 0.0N and 20.0N are displayed in Fig. 4b. 0.0N show a PL peak at ~563 nm, due to the radiative band-to-band recombination of photogenerated electrons and holes. In comparison, 20.0N exhibits a depressed PL peak, indicating that the radiative band-to-band recombination in 20.0N is suppressed compared to that of 0.0N. Furthermore, the transient-state PL spectra of 0.0N and 20.0N are presented in Fig. 4c. The charge carrier lifetimes were calculated for 0.0N and 20.0N via bi-exponential fitting of the transient-state PL spectra in Fig. 4c. The as-acquired short (*τ*_1_), long (*τ*_2_) and intensity-averaged (*τ*_ave_) lifetimes for 0.0N and 20.0N are displayed in Fig. 4c inset. The short lifetime (*τ*_1_) is mainly caused by the radiative pathways; while the long lifetime (*τ*_2_) is related to the non-radiative behaviour^11^. As shown in Fig. 4c inset, 20.0N exhibits a longer *τ*_1_ (0.20 ns) for a majority of charge carriers (97.85%), a prolonged *τ*_2_ (6.67 ns) for a very small content of charge carriers (2.15%) and an elongated *τ*_ave_ (0.34 ns), in comparison to 0.0N (*τ*_1_ = 0.18 ns; *τ*_2_ = 4.74 ns; *τ*_ave_ = 0.30 ns). As a result, the transient-state PL spectra and the corresponding lifetime fitting results together confirm that the charge carrier separation/transport in 20.0N is promoted in contrast to that in 0.0N.

Ultrafast TAS is a powerful technique for understanding behaviour of light-induced electrons and holes in photocatalysts. Hence, we used TAS to explore the charge carrier dynamics of 0.0N and 20.0N dispersed in ethanol after a 400 nm laser pulse with an energy of 0.1 μJ. As displayed in Fig. 4d, e, both the TA spectra of 0.0N and 20.0N show distinct negative and positive absorption bands, which are assigned, respectively, to the ground-state bleaching (GSB) and excited-state absorption (ESA) signals. Moreover, the ultrafast TA spectra of 0.0N and 20.0N at different pump-probe delay times are, respectively, displayed in Fig. 4f, g. The redshifts of the GSB peaks over time can be observed in both Fig. 4f, g. This is because CdS NPs in 0.0N and 20.0N possess a broad size distribution, in which the smaller CdS NPs have the larger band gaps and the faster exciton annihilation^68^. To further explore the detailed dynamic behaviours of photogenerated charge carriers, the GSB and ESA decay kinetics of both 0.0N and 20.0N are shown in Fig. 4h, i, respectively. All the fitting results are presented in Supplementary Table 10 accordingly. The suppressed GSB (Fig. 4h) and ESA (Fig. 4i) kinetic decays in the probing timescale are visible in comparison to that of 0.0N. These findings are further supported by the three-exponential fitting results of TA spectra for 0.0N and 20.0N (Supplementary Table 10). Since the three-exponential fitting results of 0.0N and 20.0N samples (Supplementary Table 10) indicate the existence of long-lived lifetime (*τ* > 3 ns), we have also collected the long-time TA spectra of 0.0N and 20.0N (up to 7.73 ns) and the results are shown in Supplementary Fig. 25a, b, respectively. The obvious negative absorption bands can be observed in both Supplementary Fig. 25a, b, ascribed to the GSB signals. Supplementary Fig. 25c, d show the ultrafast TA spectra of 0.0N and 20.0N at different pump–probe delay times, respectively. The GSB decay kinetics of 0.0N and 20.0N in the range of 0–7.73 ns and the corresponding fitting results are displayed in Supplementary Fig. 25e and Supplementary Table 11, respectively. Both the impeded GSB decay kinetics (Supplementary Fig. 25e) and elongated fitting lifetimes (Supplementary Table 11) of 20.0N compared to those of 0.0N are in accordance with the above results. Hence, the TAS results also corroborate that the coupling of NiPS_3_ UNSs with CdS NPs efficiently impedes the charge carrier recombination in 20.0N.

**Fig. 5:**
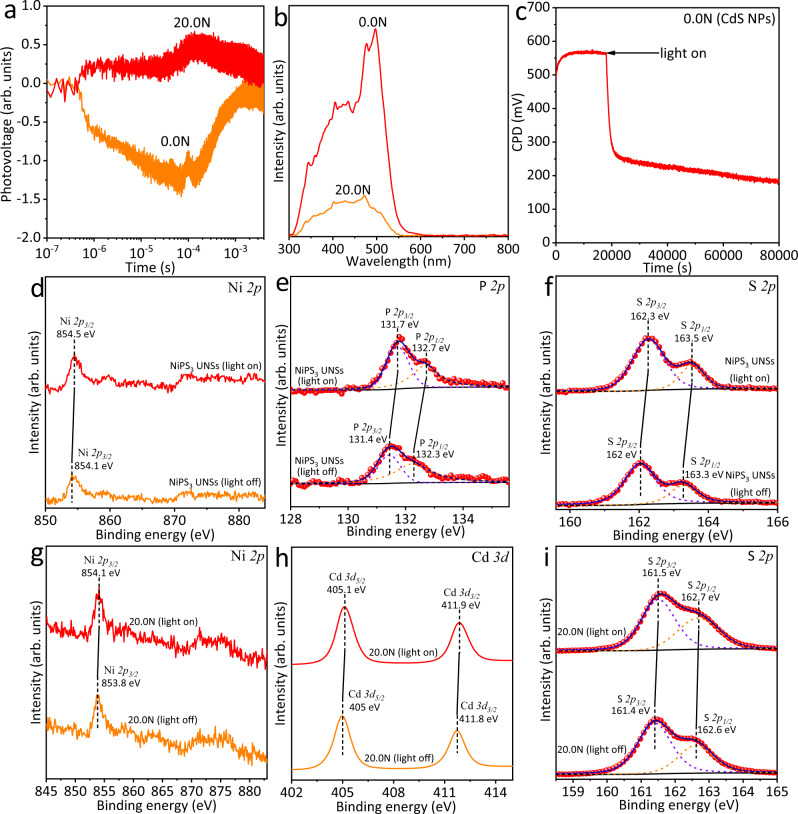
Charge carrier kinetics in NiPS_3_/CdS system. **a** Transient-state and **b** steady-state SPV spectra of 0.0N and 20.0N. **c** CPD test of 0.0N in the dark and under light irradiation. High-resolution XPS spectra of **d** Ni *2p*, **e** P *2p* and **f** S *2p* of NiPS_3_ UNSs with the light on and off, respectively. High-resolution XPS spectra of **g** Ni *2p*, **h** Cd *3d* and **i** S *2p* of 20.0N with the light on and off, respectively.

Since photocatalysis mainly involves the charge carriers at the surface/interface of photocatalysts, the behaviours of charge carriers at the surface/interface of photocatalysts were explored using a range of state-of-art techniques, such as steady-state and transient-state SPV spectroscopy, light-irradiated CPD test, and in situ XPS test. As can be seen in the transient-state SPV spectrum of 0.0N (Fig. 5a), after the excitation with a 355-nm pulse laser, a negative SPV signal is observed for 0.0N. This result indicates that compared to photo-generated holes, more photo-generated electrons migrate from the bulk to the surface of 0.0N (CdS NPs). In contrast, 20.0N exhibits a positive SPV signal in Fig. 5a. This result suggests that the loading of CdS NPs onto NiPS_3_ UNSs apparently boosts the migration of photo-generated holes from the bulk to the surface. This is attributed to the establishment of type I heterojunction between CdS NPs and NiPS_3_ UNSs, which promotes both the photogenerated electrons and holes in the CB and VB of CdS NPs to the CB and VB of NiPS_3_ UNSs, respectively (Supplementary Fig. 24). Notably, as displayed in Supplementary Fig. 24, the potential difference (0.60 V) between the VB edges of CdS NPs and NiPS_3_ UNSs is much larger than the potential difference (0.22 V) between the CB edges of CdS NPs and NiPS_3_ UNSs. From the viewpoint of thermodynamics, compared with the photogenerated electrons in CdS NPs, the photogenerated holes in CdS NPs would migrate more efficiently to NiPS_3_ UNSs in 20.0N. Thus, with light excitation, more photogenerated holes would accumulate on the surface/interface of CdS NPs and NiPS_3_ UNSs in 20.0N, resulting in a positive SPV signal in Fig. 5a. Furthermore, Fig. 5a shows that for 20.0N the absolute value of the SPV signal is reduced as compared with that of 0.0N. This is because both photogenerated electrons and holes are promoted to migrate from CdS NPs to NiPS_3_ UNSs, leading to the accumulation of both photogenerated electrons and holes on the surface/interface. As a result, for 20.0N the net value of the positive SPV signal is reduced in contrast with that of 0.0N. This is further supported by the steady-state SPV spectra of 0.0N and 20.0N (Fig. 5b) showing that for 20.0N the absolute value of the SPV signal is smaller than that of 0.0N. Moreover, Fig. 5c shows that the CPD value of 0.0N (CdS NPs) is rapidly decreased from 550 mV in the dark to 250 mV under light irradiation. This result suggests that under light excitation more photogenerated electrons than holes migrate from the bulk to the surface of CdS NPs, resulting in the upward band bending and improvement of *E*_F_. This phenomenon is in accordance with the negative value of the transient-state SPV signal for 0.0N (Fig. 5a).

Furthermore, the in situ XPS tests were also conducted to reveal the dissociation and migration of photo-generated electrons and holes on/near the surface of photocatalysts. As shown in Fig. 5d–f, the Ni *2p*, P *2p* and S *2p* peaks for NiPS_3_ UNSs shift to the high binding energy direction by 0.2–0.4 eV under light irradiation as compared to those in the dark. These results disclose that more photogenerated holes than electrons transfer from the bulk to the surface of NiPS_3_ UNSs under light illumination. Further studies show that the Ni *2p* (Fig. 5g), Cd *3d* (Fig. 5h) and S *2p* (Fig. 5i) peaks observed for 20.0N move toward the high binding energy, suggesting that for this system more photogenerated holes than electrons migrate to the surface of both CdS NPs and NiPS_3_ UNSs after light excitation. These results are in accordance with the positive transient-state SPV signal of 20.0N (Fig. 5a). However, it should be noted that the steady-state/transient-state SPV spectroscopy tests, in situ XPS tests and light-irradiated CPD test were all performed using dry powder-form of the photocatalysts, in the absence of sacrificial electron donor (triethanolamine). But in realistic photocatalytic H_2_-production tests, 0.0N or 20.0N were first dispersed in triethanolamine aqueous solution under constant stirring. Hence, these photogenerated holes migrating to the surface of CdS NPs would be rapidly captured by the sacrificial electron donor, triethanolamine, leaving the photogenerated electrons for H_2_ evolution.

Furthermore, the transient photocurrent (TPC) density measurement (Supplementary Fig. 26) displays that 20.0N electrode possesses a larger anodic current density than that of 0.0N electrode. This is ascribed to the two following reasons: (1) for 20.0N having NiPS_3_ UNSs, more photoinduced holes are transported to the surface and interface, favouring the oxidation reaction occurring at the electrode/electrolyte interface; (2) for 20.0N the promoted electron–hole separation/transfer leads to more photoinduced electrons transferred to the counter electrode for reduction reaction.

### Surface catalytic redox reactions and light absorption/excitation of NiPS_3_/CdS heterojunction

**Fig. 6:**
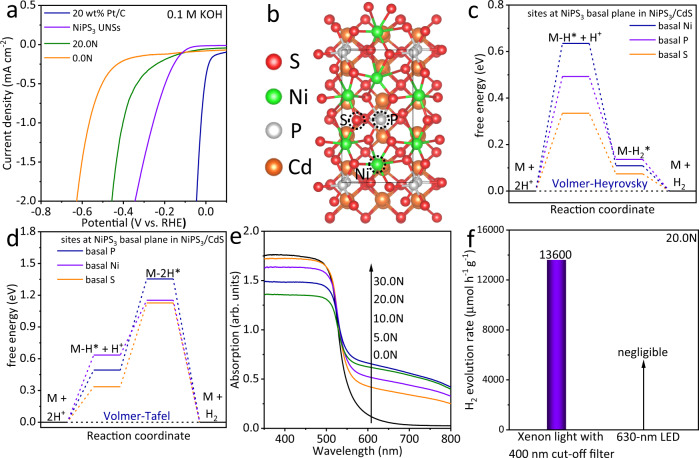
Surface catalytic reactions and light absorption of NiPS_3_/CdS system. **a** Electrochemical HER activities in 0.1 M KOH aqueous solution for 0.0N, 20.0N, NiPS_3_ UNSs and 20 wt% Pt/C. **b** top-view atomic structure of NiPS_3_/CdS showing the Ni, P and S basal sites. **c** The calculated free energy diagrams of HER following the Volmer-Heyrovsky pathway on the Ni, P and S sites of NiPS_3_ basal plane in NiPS_3_/CdS system. **d** The calculated free energy diagrams of HER following the Volmer-Tafel pathway on the Ni, P and S sites of NiPS_3_ basal plane in NiPS_3_/CdS system. **e** UV-Vis diffuse reflectance spectra of 0.0N, 5.0N, 10.0N, 20.0N and 30.0N. **f** Photocatalytic H_2_-production rates measured for 20.0N in ~17.0 vol% triethanolamine aqueous solution with xenon light irradiation (*λ* > 400 nm) and 630-nm LED, respectively. All the Gibbs free energy calculations were conducted considering the solvation effect in 17 vol% triethanolamine aqueous solution.

Apart from charge carrier separation/migration, the surface catalytic redox reactions and light absorption/excitation are the other two major factors affecting the whole photocatalysis process. For the surface catalytic redox reactions, we focus on the reduction reaction or HER on NiPS_3_/CdS (20.0N). Figure 6a presents the linear sweep voltammetry (LSV) curves for 0.0N, 20.0N, NiPS_3_ UNSs and 20 wt% Pt/C in 0.1 M KOH aqueous solution. Pure NiPS_3_ UNSs exhibit excellent HER activity (Fig. 6a), although the HER activity of NiPS_3_ UNSs is inferior to that of 20 wt% Pt/C. 20.0N presents a superior HER activity in contrast to that of 0.0N, due to the presence of NiPS_3_ UNSs with abundant atomic-level edge P/S active sites. Besides, the HER electrochemical stability tests for NiPS_3_ UNSs, NiPS_3_/TiO_2_, 20.0N, NiPS_3_/In_2_ZnS_4_ and NiPS_3_/C_3_N_4_ at the potential of −0.6 V vs. RHE are displayed in Supplementary Fig. 27a–e, respectively. The above results support that NiPS_3_ UNSs and all the composites possess excellent HER stability in 0.1 M KOH aqueous solution. This further indicates that the NiPS_3_ UNSs will not be corroded in the reduction environment in an alkaline solution. And the results also suggest the photo-generated hole is the reason causing the deactivation of NiPS_3_ UNSs active sites. Additionally, the DFT-based computations reveal that the combination of NiPS_3_ with CdS leads to the greatly-reduced Δ*G*_H*_ values on P and S sites at the basal plane of NiPS_3_ monolayer in NiPS_3_/CdS system (Fig. 6b–d and Supplementary Table 12), in comparison to those of pure NiPS_3_ monolayer (Supplementary Fig. 1a–c and Supplementary Table 2). Especially, the inactive S site on the basal plane of NiPS_3_ monolayer becomes much more active in NiPS_3_/CdS system for HER following the Volmer-Heyrovsky pathway (Supplementary Tables 2 and 12). In contrast, the ΔG_H*_ values on Ni site at the basal plane of NiPS_3_ monolayer in NiPS_3_/CdS are obviously increased for the first Volmer step (Supplementary Table 12), compared to the Ni site at the basal plane of NiPS_3_ monolayer alone (Supplementary Table 2). This result suggests the reduced HER activity of Ni site after the combination of NiPS_3_ and CdS. Overall, the strong electronic combination of NiPS_3_ with CdS leads to the activated S site of NiPS_3_ monolayer in NiPS_3_/CdS system. For the surface catalytic oxidation reaction, since more photogenerated holes migrate to the surface of 20.0N after coupling CdS and NiPS_3_, the oxidation of electron donor, triethanolamine, to the oxidation product(s) is facilitated. Moreover, the N_2_ sorption isotherm measured for 20.0N shifts downs compared with that of 0.0N (Supplementary Fig. 28). Consequently, the Brunauer–Emmett–Teller (BET) surface area and pore volume evaluated for 20.0N are also slightly lower than those obtained for 0.0N (Supplementary Table 7). This is due to the mild aggregation of CdS NPs in 20.0N after evaporating ethanol in the physical mixing process. These results also indicate that the surface area does not have a significant impact on the photocatalytic activity of NiPS_3_/CdS system.

Since NiPS_3_ UNSs possess a small band gap of 1.42 eV with a wide light absorption spectrum (up to 873 nm), intensified visible-light absorption is observed for 5.0N, 10.0N, 20.0N and 30.0N (Fig. 6e). The visible-light absorption is gradually improved with an increasing amount of NiPS_3_ UNSs, i.e, from 5.0N, 10.0N, 20.0N to 30.0N (Fig. 6e). To study whether the increased visible-light absorption of 20.0N (Fig. 6e) contributed to the high H_2_-production rate (Fig. 4a), we tested the photocatalytic H_2_-production rate of 20.0N under 630-nm light emitting diode (LED) illumination. However, 20.0N exhibits a negligible photocatalytic H_2_ production under 630-nm LED light irradiation (Fig. 6f). This is because the photons of 630-nm LED light (*hν* = 1.97 eV) can only excite NiPS_3_ UNSs rather than CdS NPs. However, the weak photogenerated holes in the VB of NiPS_3_ UNSs (Supplementary Fig. 10) cannot oxidize the electron donor, triethanolamine. Thus, the photogenerated electrons and holes rapidly recombine with each other, leading to negligible photocatalytic H_2_ production on pure NiPS_3_ UNSs. This also suggests that the strengthened absorption observed for NiPS_3_ UNSs (Fig. 6e) does not contribute to the improved photocatalytic activity.

### Photocatalytic mechanism for NiPS_3_/CdS heterojunction

**Fig. 7:**
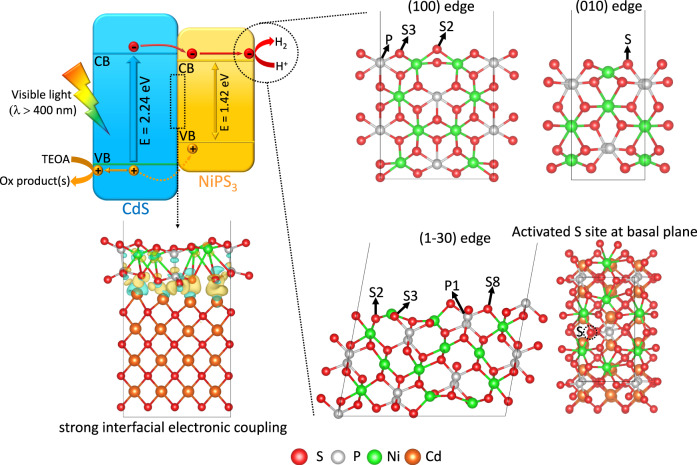
Schematic illustration of photocatalytic H_2_-production mechanism in NiPS_3_/CdS system. The visible-light excitation (*λ* > 400 nm), separation/migration of photogenerated electrons and holes, and the surface catalytic reactions of NiPS_3_/CdS system are shown in the figure.

Based on the aforementioned experiments and calculations, a photocatalytic mechanism is proposed for the NiPS_3_/CdS heterojunction (Fig. 7). First, under visible-light irradiation (*λ* > 400 nm), both CdS NPs and NiPS_3_ UNSs are excited and photoinduced electrons and holes are, respectively, generated on the CB and VB. Due to the formation of type I heterojunction (straddling type) between CdS NPs and NiPS_3_ UNSs, together with the strong interfacial electronic coupling between CdS NPs and NiPS_3_ UNSs (Fig. 7), the photoinduced electrons and holes should, respectively, migrate from the CB and VB of CdS NPs to those of NiPS_3_ UNSs. However, in the existence of a sacrificial electron donor, triethanolamine, most of the photogenerated holes are actually harvested and consumed by triethanolamine molecules on the surface of CdS NPs, generating oxidation product(s). Only a small amount of the photo-generated holes in the VB of CdS NPs is transferred to the VB of NiPS_3_ UNSs (Fig. 7). In contrast, much more photogenerated electrons in the CB of CdS NPs are transported to the CB of NiPS_3_ UNSs. As shown in Fig. 7, four kinds of HER reactive sites are present on NiPS_3_: (1) P, S2 and S3 sites at (100) edge, (2) S site at (010) edge, (3) P1, S2, S3 and S8 sites at (1−30) edge and (4) activated S site at the basal plane. Hence, the photogenerated electrons could efficiently reduce the protons to evolve H_2_ gas molecules at the above four kinds of highly active HER sites on NiPS_3_ UNSs in NiPS_3_/CdS system. Therefore, this NiPS_3_/CdS heterojunction assures both efficient electron-hole dissociation/migration and boosts HER activity, thus leading to the highly enlarged photocatalytic H_2_ production (Fig. 4a). Additionally, since the enhanced visible-light absorption by NiPS_3_ UNSs cannot contribute to the photocatalytic H_2_ production, the light absorption and generation of photoinduced charge carriers by NiPS_3_ UNSs are not depicted in Fig. 7. This mechanism demonstrates that the type I heterojunction can also achieve highly efficient photocatalytic performance in some conditions.

### Generality of NiPS_3_ UNSs

To support the generality of NiPS_3_ UNSs platform for advancing photocatalytic H_2_ production, the other three systems were also explored. The XRD patterns of TiO_2_ and NiPS_3_/TiO_2_ (Supplementary Fig. 29a), In_2_ZnS_4_ and NiPS_3_/In_2_ZnS_4_ (Supplementary Fig. 29b), as well as C_3_N_4_ and NiPS_3_/C_3_N_4_ (Supplementary Fig. 29c) are displayed in the supporting information. As shown in Supplementary Fig. 29a–c, no obvious alteration in the positions and intensities of diffraction peaks are observed on the patterns for hybrid samples (NiPS_3_/TiO_2_, NiPS_3_/In_2_ZnS_4_ and NiPS_3_/C_3_N_4_), in contrast to their pure counterparts (TiO_2_, In_2_ZnS_4_ and C_3_N_4_). These results indicate that the physical mixing process in the mortar at room temperature does not alter the crystal/phase structures of TiO_2_, In_2_ZnS_4_ or C_3_N_4_. No diffraction peaks assigned to NiPS_3_ UNSs are found on the XRD patterns of NiPS_3_/TiO_2_, NiPS_3_/In_2_ZnS_4_ and NiPS_3_/C_3_N_4_ (Supplementary Fig. 29a–c), suggesting the homogenous dispersion and low amount of NiPS_3_ UNSs in the merged samples (NiPS_3_/TiO_2_, NiPS_3_/In_2_ZnS_4_ and NiPS_3_/C_3_N_4_).

The TEM images of NiPS_3_/TiO_2_ (Supplementary Fig. 30a), NiPS_3_/In_2_ZnS_4_ (Supplementary Fig. 30b) and NiPS_3_/C_3_N_4_ (Supplementary Fig. 30c), respectively, confirm the combination of NiPS_3_ UNSs with TiO_2_ NPs, In_2_ZnS_4_ NSs and C_3_N_4_, respectively. Also, the HRTEM images (Supplementary Fig. 30d–f) as well as the EDX spectra (Supplementary Fig. 30g–i) further support the merging of NiPS_3_ UNSs with TiO_2_ NPs, In_2_ZnS_4_ NSs and C_3_N_4,_ respectively. Moreover, the HAADF-STEM images and the corresponding elemental mapping images of NiPS_3_/TiO_2_ (Supplementary Fig. 31a–f), NiPS_3_/In_2_ZnS_4_ (Supplementary Fig. 31g–l) and NiPS_3_/C_3_N_4_ (Supplementary Fig. 31m–r) also support coupling of NiPS_3_ UNSs with TiO_2_ NPs, In_2_ZnS_4_ NSs and C_3_N_4,_ respectively. The high-resolution XPS spectra of NiPS_3_/TiO_2_ (Supplementary Fig. 17a–d), NiPS_3_/In_2_ZnS_4_ (Supplementary Fig. 17e–h) and NiPS_3_/C_3_N_4_ (Supplementary Fig. 17i–l) confirm the intimate electronic coupling between NiPS_3_ and TiO_2_, In_2_ZnS_4_, C_3_N_4_, respectively. The efficient charge separation and transport in NiPS_3_/TiO_2_, NiPS_3_/In_2_ZnS_4_ and NiPS_3_/C_3_N_4_ are supported by both the steady-state PL spectra in Supplementary Fig. 32a–c and transient-state PL spectra in Supplementary Fig. 32d–f. The data presented in Supplementary Fig. 32d–f insets also prove that all the fitting charge carrier lifetimes and intensity-averaged charge carrier lifetimes obtained for NiPS_3_/TiO_2_, NiPS_3_/In_2_ZnS_4_ and NiPS_3_/C_3_N_4_ are elongated after loading of NiPS_3_ UNSs, compared to the bare counterparts, respectively. To study the intimate interaction in all the NiPS_3_-based samples, the Zeta potentials of NiPS_3_ UNSs, TiO_2_ NPs, CdS NPs, In_2_ZnS_4_ NSs and C_3_N_4_ in ethanol were acquired. As shown in Supplementary Fig. 33, NiPS_3_ UNSs, TiO_2_ NPs, CdS NPs, In_2_ZnS_4_ NSs and C_3_N_4_ show the Zeta potentials of −2.73, −0.36, −57.3, −58 and 12.5 mV in ethanol, respectively. These results indicate the repulsive electrostatic forces between NiPS_3_ UNSs and TiO_2_ NPs, CdS NPs or In_2_ZnS_4_ NSs. However, the TEM images (Fig. 2a and Supplementary Fig. 30a, b) confirm the combination of NiPS_3_ UNSs with TiO_2_ NPs, CdS NPs and In_2_ZnS_4_ NSs, respectively. And the XPS results (Fig. 3a and Supplementary Fig. 17a–h) further indicate the electronic coupling between NiPS_3_ UNSs and TiO_2_ NPs, CdS NPs or In_2_ZnS_4_ NSs. These results suggest that Van der Waals force rather than the electrostatic force should be the main force leading to the strong electronic coupling in NiPS_3_/CdS, NiPS_3_/TiO_2_ and NiPS_3_/In_2_ZnS_4_ systems. Furthermore, as for the NiPS_3_/C_3_N_4_ system, the opposite Zeta potentials for NiPS_3_ (−2.73 mV) and C_3_N_4_ (12.5 mV) are observed in Supplementary Fig. 33. Thus, the attractive electrostatic force and Van der Waals force together result in the strong coupling between NiPS_3_ UNSs and C_3_N_4_, as evidenced by the TEM image (Supplementary Fig. 30c) and XPS results (Supplementary Fig. 17i–l).

The electrochemical HER activities shown in Supplementary Fig. 34a–c further prove that the integration of NiPS_3_ UNSs with TiO_2_ NPs, In_2_ZnS_4_ NSs and C_3_N_4_ can greatly boost the HER activities. In addition, the UV-Vis diffuse reflectance spectra of TiO_2_ and NiPS_3_/TiO_2_ (Supplementary Fig. 35a), In_2_ZnS_4_ and NiPS_3_/In_2_ZnS_4_ (Supplementary Fig. 35b), as well as C_3_N_4_ and NiPS_3_/C_3_N_4_ (Supplementary Fig. 35c) are also presented in supporting information. The addition of NiPS_3_ UNSs leads to greatly improved visible-light absorption owing to the strong absorption of NiPS_3_ with a small band gap (*E* = 1.42 eV). However, under the illumination of 630-nm LED, NiPS_3_/TiO_2_, NiPS_3_/In_2_ZnS_4_ and NiPS_3_/C_3_N_4_ exhibit negligible photocatalytic H_2_-production activity (Supplementary Fig. 35d–f), again suggesting that the increased light absorption caused by NiPS_3_ UNSs does not contribute to the activity enhancement. The above-mentioned results strongly support the ability of NiPS_3_ UNSs to efficiently improve the photocatalytic H_2_-production rates for different kinds of semiconductor photocatalysts, which demonstrates a huge potential of the emerging MPC_x_ family in photocatalysis.

This work shows that the edge of NiPS_3_ UNSs possesses abundant atomic-level P/S active sites while the basal plane exhibits relatively lower HER activity. Although it is hard to selectively load the CdS NPs at the edge rather than the basal plane of NiPS_3_ UNSs for achieving further activity improvement, NiPS_3_ UNSs with smaller lateral sizes and more edge active sites can be synthesized to facilitate the loading of CdS NPs at/near the edge of NiPS_3_ UNSs. This will be explored in our future study.

## Discussion

In summary, we report for the first time a facile liquid exfoliation technique to synthesize the MPC_*x*_ group of materials, 2D NiPS_3_, with an ultrathin thickness (~3.16 nm). The as-synthesized NiPS_3_ UNSs serve as a universal platform to elevate the light-driven H_2_-production performance of various photocatalysts, including TiO_2_, CdS, In_2_ZnS_4_ and C_3_N_4_. The as-prepared NiPS_3_/CdS hybrid displays the highest photocatalytic hydrogen (H_2_) production activity (13,600 μmol h^−1^ g^−1^), with the largest enhancement factor of ~1667%, in contrast to that of pristine CdS. The greatly raised performance of NiPS_3_/CdS is due to two reasons: (1) the electronically coupled interfaces between NiPS_3_ UNSs and CdS NPs apparently facilitate the charge-carrier separation/transport. Particularly, the transport of photogenerated holes to the surface of CdS NPs is significantly boosted, which are harvested by the sacrificial electron donor, triethanolamine. Thus, the remaining photogenerated electrons on CdS NPs could efficiently migrate to NiPS_3_ UNSs for H_2_ evolution; (2) numerous atomic-level P/S edge sites and activated S basal sites of NiPS_3_ UNSs tremendously advance H_2_ evolution reaction. These findings are supported by both theoretical computations and advanced characterizations, such as atomic-resolution AC-STEM, transient-state PL spectroscopy, transient-state SPV spectroscopy, ultrafast TAS and in situ XPS. Our study not only shows the great potential of MPC_*x*_ family as a general platform to immensely increase the light-induced H_2_-production activities on various semiconductor photocatalysts but more importantly, shed light on the rational design/preparation of photocatalysts through understanding of the atomic-level structure/composition-activity correlation and electron-hole kinetics/thermodynamics in photocatalysis.

## Methods

### Synthesis of NiPS_3_ UNSs

NiPS_3_ UNSs were fabricated by a liquid exfoliation method. In detail, 50 mg of commercial bulk NiPS_3_ powder (Ossila, UK) were ground into a fine powder and added to 50 mL of ethanol and ultrasonicated in an ice bath for 200 min. The resultant dispersion was centrifuged at 8000 RPM (6016 × *g*) for 2 min and the supernatant was reserved for use. The concentration of NiPS_3_ UNSs in ethanol solution was determined to be ~109.97 μg mL^−1^ using ICP-AES.

### Synthesis of CdS NPs

CdS NPs were synthesized using a precipitation-hydrothermal technique. First, 3.424 g of Cd(NO_3_)_2_·4H_2_O were put into 87 mL of deionized water followed by stirring for 1 h. Then, 20 mL of 0.9 M Na_2_S aqueous solution were added dropwise into the above solution, followed by 1-h stirring. At last, the suspension was transferred to a 200 mL Teflon-lined autoclave and maintained at 180 °C for 12 h. The resulting products were washed with deionized water and ethanol twice, respectively, and dried at 60 °C for 300 min. CdS NPs were denoted as 0.0N.

### Synthesis of In_2_ZnS_4_ nanosheets (NSs)

In_2_ZnS_4_ NSs were fabricated utilizing a hydrothermal method. Specifically, 0.277 g of Zn(NO_3_)_2_·6H_2_O, 0.560 g of In(NO_3_)_3_·*x*H_2_O and 0.559 g of thioacetamide were put into 140 mL of deionized water with constant stirring. Afterward, 21.1 mL of 1.0 M HCl aqueous solution were added to the above aqueous solution. The resulting aqueous solution was transferred to a 200 mL hydrothermal autoclave and kept at 160 °C for 12 h.

### Synthesis of C_3_N_4_

C_3_N_4_ was synthesized via grinding 5 g of urea, 5 g of thiourea and 20 mg of Pluronic F127 and adding the resulting mixture into a crucible followed by heating at 350 °C for 1 h and 600 °C for 3 h. The as-synthesized sample was ground into fine powder.

### Synthesis of NiPS_3_/CdS heterojunction

NiPS_3_/CdS heterostructure was synthesized by a self-assembly approach via mechanical mixing at room temperature. Specifically, 50 mg of the as-synthesized CdS NPs were put into the mortar, followed by adding 5.0, 10.0, 20.0 and 30.0 mL of NiPS_3_ UNSs in ethanol, respectively. The suspension was then ground for 1 min. After evaporation of ethanol, the solid samples were finally ground to obtain fine powders and denoted as 5.0N, 10.0N, 20.0N and 30.0N, respectively.

### Synthesis of NiPS_3_/In_2_ZnS_4_ heterojunction

NiPS_3_/In_2_ZnS_4_ heterostructure was fabricated using a self-assembly method through physical mixing at room temperature. In detail, 50 mg of the as-synthesized In_2_ZnS_4_ NSs were added into the mortar followed by adding 20.0 mL of NiPS_3_ UNSs in ethanol. The suspension was then ground for 1 min. After evaporation of ethanol, the solid sample was finally ground to a fine powder.

### Synthesis of NiPS_3_/TiO_2_ heterojunction

NiPS_3_/TiO_2_ heterostructure was fabricated by a self-assembly method through mechanical mixing at room temperature. In detail, 50 mg of the as-synthesized Degussa P25 TiO_2_ NPs were added into the mortar, followed by adding 20.0 mL of NiPS_3_ UNSs in ethanol. The suspension was then ground for 1 min. After evaporation of ethanol, the solid sample was finally ground to obtain a fine powder.

### Synthesis of NiPS_3_/C_3_N_4_ heterojunction

NiPS_3_/C_3_N_4_ heterojunction was synthesized by a self-assembly approach through mechanical mixing at room temperature. In detail, 50 mg of the as-synthesized C_3_N_4_ were added into the mortar, followed by adding 20.0 mL of NiPS_3_ UNSs in ethanol. The suspension was then ground for 1 min. After evaporation of ethanol, the solid sample was finally ground to obtain a fine powder.

### Physicochemical characterizations

The XRD patterns of 0.0N, 5.0N, 10.0N, 20.0N, 30.0N and 20.0N-A were acquired on a silicon substrate on a powder X-ray diffractometer (D4 ENDEAVOR, Bruker) utilizing Co Kα radiation. The XRD patterns of In_2_ZnS_4_, NiPS_3_/In_2_ZnS_4_, TiO_2_, NiPS_3_/TiO_2_, C_3_N_4_ and NiPS_3_/C_3_N_4_ were obtained on a powder X-ray diffractometer (Miniflex, Rigaku) utilizing Cu Kα radiation. The FEI Themis Z double corrected S/TEM (Thermo Fisher Scientific, USA), FEI Titan S/TEM (Thermo Fisher Scientific, USA) and FEI tecani G2 Spirit TEM (Thermo Fisher Scientific, USA) were applied to acquire the TEM images, HRTEM images, EDS spectra, HAADF-STEM images, elemental mapping images and EELS spectra. A Multimode 8 (Bruker, USA) was used to obtain the AFM image and the height profile accordingly. The AFM profile was acquired after flattening treatment. An iHR550 Raman microscope (HORIBA scientific) with a charge-coupled device (CCD) detector and a confocal microscope were applied to acquire the Raman spectra. An Optima 8000 ICP-OES (Perkin Elmer, UK) was utilized to obtain the actual NiPS_3_ amount in 20.0N sample. The in situ and ex-situ XPS measurements were performed on a K-Alpha plus XPS system (Thermo Fisher Scientific, USA). A light emitting diode was adopted as the light source to excite the photocatalysts in the in situ XPS measurements. A UV-Vis spectrophotometer (UV2600, Shimadzu, Japan) was utilized to obtain the UV-Vis diffuse reflectance spectra and UV-Vis absorption spectra. N_2_ sorption analysis was conducted on a tristar II 3020 (Micromeritics, USA). A RF-5301PC spectrofluorophotometer (Shimadzu, Japan) was used to obtain the steady-state photoluminescence (PL) spectra at room temperature. A FLS1000 spectrometer (Edinburgh Instruments, UK) was utilized to acquire the transient-state PL spectra. The synchrotron radiation-based XANES measurements were performed at Hefei Synchrotron Radiation Facility (MCD-A and MCD-B Soochow Beamline for Energy Materials, NSRL) and XAS beamline in Australian Synchrotron (AS). A home-built apparatus introduced by Jing et al.^69^ was used to acquire the steady-state SPV spectra. Another device described in the previous reference was adopted to obtain the transient-state SPV spectra. TAS used laser pulses sourced from the output of a Ti: sapphire regenerative amplifier (Spectra Physics, Spitfire Pro XP 100F), providing pulses centred at 800 nm with 100 fs duration and a 1 kHz repetition rate. The 400 nm excitation pulse was generated by frequency doubling of the fundamental output using a 0.5 mm BBO crystal. Pump-probe spectroscopic experiments were performed on a TA spectrometer (Ultrafast Systems, Helios). The 400 nm pump pulses had an energy of 0.1 μJ with a fwhm spot size of 350 μm, with a polarization rotated to the magic angle relative to the probe. The visible probe light was produced by focusing a small portion of the 800 nm amplifier output onto a 3.2 mm sapphire crystal. The white-light continuum was then split into signal and reference beams and focused onto the sample with a fwhm spot size of 100 μm. Samples were continuously stirred throughout the experiment, and photodegradation was <5%. The long-time TA spectra (up to 7.73 ns) were collected using a 400 nm laser with a power of 120 μW. The contact potential difference (CPD) test was measured on a Kelvin probe apparatus (Instytut Fotonowy, Poland) using the excitation light source of 427 nm. The Zeta potentials were acquired on a Zetasizer nano instrument (Malvern Panalytical, UK).

### Photocatalytic H_2_ production test

The photocatalytic H_2_-production test was performed in a 152 mL Pyrex flask with sealed silicone rubber septa at room temperature and atmospheric pressure. The light source is a 300 W xenon arc lamp with a UV-cutoff filter (*λ* > 400 nm) for testing CdS-, In_2_ZnS_4_- and C_3_N_4_-based photocatalysts, and without any UV-cutoff filter for testing TiO_2_ based photocatalysts. In a typical test, 20 mg of photocatalyst were added into 80 mL of ~17.0 vol% triethanolamine aqueous solution. Subsequently, ultrahigh purity Argon gas was purged into the suspension of photocatalyst for 30 min to remove air prior to illumination and make sure the anaerobic condition of the reactor. After that, 200 μL of gas were sampled intermittently through the septum followed by examining the generated H_2_ on a gas chromatograph (Clarus 480, Perkin Elmer, USA) with a TDX-01 column and ultrahigh Argon as the carrier gas. Photocatalytic H_2_-production test using 630-nm LED was performed in the same conditions except that 300 W Xenon arc lamp was replaced by a 77 W 630-nm LED. The photocatalytic H_2_-production stability test of 20.0N in 0.35 M Na_2_S and 0.25 M Na_2_SO_3_ aqueous solution was conducted in identical conditions except that ~17.0 vol% triethanolamine aqueous solution was replaced by the 0.35 M Na_2_S and 0.25 M Na_2_SO_3_ aqueous solution.

### Apparent quantum yield test

The apparent quantum yield (AQY) test was conducted in a top-irradiated 254 mL reactor using 70 mg 20.0N photocatalysts in 70 mL ~17.0 vol% triethanolamine aqueous solution. A 420-nm LED with a light intensity of 16.8 mW cm^−2^ was utilized as the light source. The AQY was calculated according to the following equation:

1 $${{{\mathrm{AQY}}}}\;(\%)=	\frac{{{{\mathrm{Reacted}}}}\;{{{\mathrm{electron}}}\;{{{\mathrm{number}}}}}}{{{{\mathrm{Incident}}}}\;{{{\mathrm{photon}}}}\;{{{\mathrm{number}}}}}\times 100 \\=	\frac{{{{\mathrm{Generated}}}}\;{{{\mbox{H}}}}_{2}\;{{{\mathrm{molecule}}}}\; {{{\mathrm{number}}}}\times 2}{{{{\mathrm{Incident}}}}\;{{{\mathrm{photon}}}} \;{{{\mathrm{number}}}}}\times 100$$

### Electrochemical and photoelectrochemical measurements

The Mott-Schottky plots were acquired using an electrochemical analyser (CHI760E instruments) in 0.5 M Na_2_SO_4_ aqueous solution with a standard three-electrode system. Moreover, the identical three-electrode system was utilized to measure the TPC density in 0.5 M Na_2_SO_4_ aqueous solution. A 300 W xenon light with a UV-cutoff filter (*λ* > 400 nm) was utilized as the light source. 0.0N or 20.0N working electrode was prepared in a process as below: 10 mg of 0.0N or 20.0N, 15 mg of polyethylene glycol (PEG; molecular weight: 20,000) and 1.0 mL of ethanol were ground to produce a slurry. Then 0.1 mL of the slurry (equivalent to ~1 mg of 0.0N or 20.0N and 1.5 mg of PEG) was coated onto a 12 mm × 8 mm FTO glass electrode through a doctor-blade method. Finally, the coated electrode was dried and heated at 350 °C for 30 min with flowing ultrahigh purity argon flow. The NiPS_3_ working electrode was synthesized as follows: 2 mL of NiPS_3_ UNSs in ethanol (100 drops with 20 μL each time; equivalent to ~219.94 μg NiPS_3_ UNSs) were dispersed onto the surface of a 12 mm × 8 mm FTO glass electrode. The acquired electrode was naturally dried in air and heated at 350 °C for 0.5 h under ultrahigh purity argon flow. Electrocatalyst ink was prepared by dispersing catalyst powder (2 mg) in a solution containing distilled water (Milli-Q, 482.5 μL), ethanol (482.5 μL) and 5 wt % Nafion solution (35 μL) followed by ultrasonication for 2 h. In all, 10 μL of catalyst ink was then deposited onto a polished glassy carbon electrode (diameter = 5 mm, area = 0.196 cm^2^, Pine Research Instrument). The mass loading of catalysts was determined as ~102 μg cm^−2^. Electrochemical experiments were carried out using the rotating disk electrode method in a standard three-electrode glass cell at room temperature. A carbon rod was used as the counter electrode and an Ag/AgCl electrode (Pine Research Instrumentation) was used as the reference electrode. All potentials were converted to the reversible hydrogen electrode (RHE) and corrected with 95% iR-compensation. HER measurements were conducted in argon-saturated 0.1 M KOH electrolyte with a CHI potentiostat (CHI 760D) at a rotating speed of 1600 rpm. The polarization curves were recorded with a sweeping rate of 5 mV s^−1^. The stability test was performed with 20 μL of catalyst ink deposited onto carbon paper (area = ~0.4 cm^2^, the mass loading of catalysts was ~100 μg cm^−2^) under a potential of −0.6 V vs. RHE.

### DFT calculations

DFT calculations were applied to conduct all the electronic structure optimizations and calculations in this study. All the DFT calculations were conducted considering the solvation effect in 17 vol% triethanolamine aqueous solution using the VASPSOL, in which photocatalytic HER occurs. The Perdew–Burke–Ernzerhof (PBE) generalized gradient approximation (GGA) exchange–correlation functionals were applied using the projector-enhanced wave method through VASP software. Considering the solvent effect in 17% triethanolamine aqueous solution by volume, we set the dielectric constant of the solution to 29.36 and the Debye length to 2.68 Å. According to different cut-off energy tests, a plane wave cut-off energy of 520 eV was set, and the energy error is 0.01 eV. The convergence criteria for structural relaxation and energy calculation are: (1) self-consistent field energy tolerance is 1.0 × 10^-5^ eV; (2) all the atoms in the systems were fully relaxed and maximum force tolerance on each atom is smaller than 0.05 eV Å^−1^. During the geometry optimization and the total energy calculations, the smearing value was set as 0.2 eV. The approximate method developed by Grimme et al.^70^ (zero damping DFT-D3 method of Grimme) was applied during all calculations to account for the contribution of the Van der Waals interactions between atoms to the energy. The structural optimization parameters for acquiring the hydrogen adsorption Gibbs free energy are displayed as follows: (i) (002) basal plane of NiPS_3_: unit cell parameters, *a* = 5.82 Å, *b* = 10.08 Å, *c* = 43.04 Å, *α* = 90º, *β* = 90º, *γ* = 90º, *K*-points setting of 5 × 3 × 1. (ii) (100) edge of NiPS_3_: unit cell parameters, *a* = 10.08 Å, *b* = 34.92 Å, *c* = 43.04 Å, *α* = 90º, *β* = 90º, *γ* = 90º, *K*-points setting of 3 × 1 × 1. (iii) (010) edge of NiPS_3_: unit cell parameters, *a* = 5.82 Å, *b* = 30.25 Å, *c* = 43.04 Å, *α* = 90º, *β* = 90º, *γ* = 90º, *K*-points setting of 5 × 1 × 1. (iv) (1−30) edge of NiPS_3:_ unit cell parameters, *a* = 6.90 Å, *b* = 15.40 Å, *c* = 33.65 Å, *α* = 79.33º, *β* = 94.87º, *γ* = 93.02º, *K*-points setting of 5 × 1 × 1. (v) NiPS_3_ (002) facet/CdS (200) facet heterostructure: unit cell parameters, *a* = 5.84 Å, *b* = 10.91 Å, *c* = 57.25 Å, *α* = 90º, *β* = 90º, *γ* = 90º, *K*-points setting of 5 × 3 × 1. The structural optimization parameters for acquiring the work functions (Φ) are shown as follows: (i) (200) facet of CdS (space group F-43m): *a* = 5.86 Å, *b* = 11.73 Å, *c* = 50.26 Å, *α* = *β* = *γ* = 90º, *K*-points setting of 5 × 3 × 1. (ii) (002) facet of NiPS_3_ (space group C2/m): *a* = 5.82 Å, *b* = 10.08 Å, *c* = 43.04 Å, *α* = *β* = *γ* = 90º, *K*-points setting of 5 × 3 × 1. The structural optimization parameters for acquiring the differential charge density map of NiPS_3_ (002) facet/CdS (200) facet CdS heterostructure are shown as follows: *a* = 5.84 Å, *b* = 10.91 Å, *c* = 57.25 Å, *α* = 90º, *β* = 90º, *γ* = 90º, *K*-points setting of 5 × 3 × 1. The HSE06 hybrid functional was used to calculate the band structures and DOS of CdS (200) facet and NiPS_3_. The plane wave cut-off energy of 520 eV was set, and the energy error is 0.01 eV. The self-consistent field energy tolerance is 1.0 × 10^−5^ eV. The calculation path of CdS (200) facet band structure is G-X-M-G, and the numbers of *K*-points are 17, 8 and 20. The calculation path of NiPS_3_ band structure is G-M-K-G, and the numbers of *K*-points are 15, 8 and 17. The lattice parameters of CdS (200) facet and NiPS_3_ are as follows: (i) CdS (200) facet, *a* = 5.86 Å, *b* = 11.73 Å, *c* = 21.46 Å, *α* = *β* = *γ* = 90º. (ii) NiPS_3_, *a* = 5.82 Å, *b* = 5.82 Å, *c* = 26.28 Å, *α* = *β* = 90º, *γ* = 120.02º. The atomic coordinates of the above structures are displayed in Supplementary Tables 13–18.

## Supplementary information

Supplementary Information

## Data Availability

All data supporting the findings of this study are available in the article and its Supplementary Information. Source data are provided with this paper.

## Source data

Source Data
