# Supplementary material for: NiPS3 ultrathin nanosheets as versatile platform advancing highly active photocatalytic H2 production
Source: Nat Commun. 2022 Aug 6;13:4600. doi: 10.1038/s41467-022-32256-6 (PMC9357043; doi:10.1038/s41467-022-32256-6)
Supplement: Supplementary file 1 — Supplementary Information [file 41467_2022_32256_MOESM1_ESM.pdf]

Supplementary Information for

$NiPS_3$ ultrathin nanosheets as versatile platform advancing highly active photocatalytic H_2 production

Jingrun Ran[1†], Hongping Zhang[2†], Sijia Fu[1†], Mietek Jaroniec,[3] Jieqiong Shan[1], Bingquan Xia[1], Yang Qu[4], Jiangtao Qu[5], Shuangming Chen[6], Li Song[6], Julie M. Cairney[7], Liqiang Jing[4], Shi-Zhang Qiao[1]*

[1] School of Chemical Engineering and Advanced Materials, University of Adelaide, Adelaide, South Australia 5005, Australia

[2] State Key Laboratory of Environmentally Friendly Energy Materials, Engineering Research Center of Biomass Materials (Ministry of Education), School of Materials Science and Engineering, Southwest University of Science and Technology, Mianyang, Sichuan 621010, China

[3] Department of Chemistry and Biochemistry & Advanced Materials and Liquid Crystal Institute, Kent State University, Kent, Ohio 44242, United States

[4] Key Laboratory of Functional Inorganic Material Chemistry (Ministry of Education), School of Chemistry and Materials Science, International Joint Research Center for Catalytic Technology, Heilongjiang University, Harbin 150080, P. R. China

[5] Australian Centre for Microscopy and Microanalysis, the University of Sydney, New South Wales 2006, Australia

[6] National Synchrotron Radiation Laboratory, CAS Center for Excellence in Nanoscience, University of Science and Technology of China, Hefei, Anhui 230029, P. R. China

[7] School of Physics, The University of Sydney, Sydney, New South Wales 2006, Australia

* Correspondence to: s.qiao@adelaide.edu.au

† These authors contributed equally to this work.

Supplementary Results

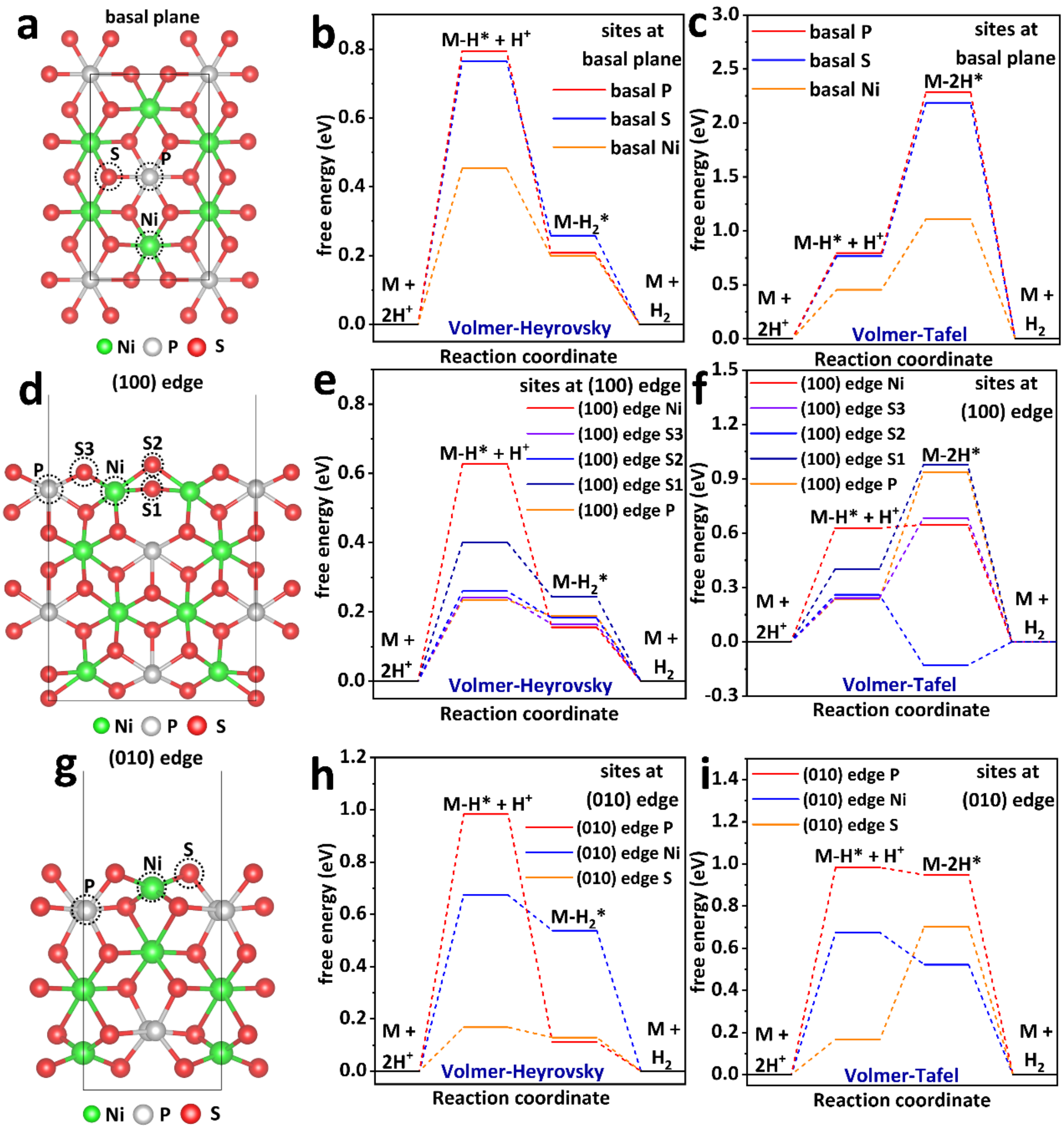

Supplementary Fig. 1. Sites calculated for basal plane, (100) edge and (010) edge of $NiPS_3$ monolayer. (a) sites calculated for hydrogen adsorption on basal plane of $NiPS_3$ monolayer. Free energy diagrams for HER following the (b) Volmer-Heyrovsky pathway and (c) Volmer-Tafel pathway for the sites at basal plane of $NiPS_3$ monolayer. (d) sites calculated for hydrogen adsorption on (100) edge of $NiPS_3$ monolayer. Free energy diagrams for HER following the (e) Volmer-Heyrovsky pathway and (f) Volmer-Tafel pathway for the sites at (100) edge of $NiPS_3$ monolayer. (g) sites calculated for hydrogen adsorption on (010) edge of $NiPS_3$ monolayer. Free energy diagrams for HER following the (h) Volmer-Heyrovsky pathway and (i) Volmer-Tafel pathway for the sites at (010) edge of $NiPS_3$ monolayer. All the Gibbs energy calculations were conducted considering the solvation effect in 17 vol% triethanolamine aqueous solution.

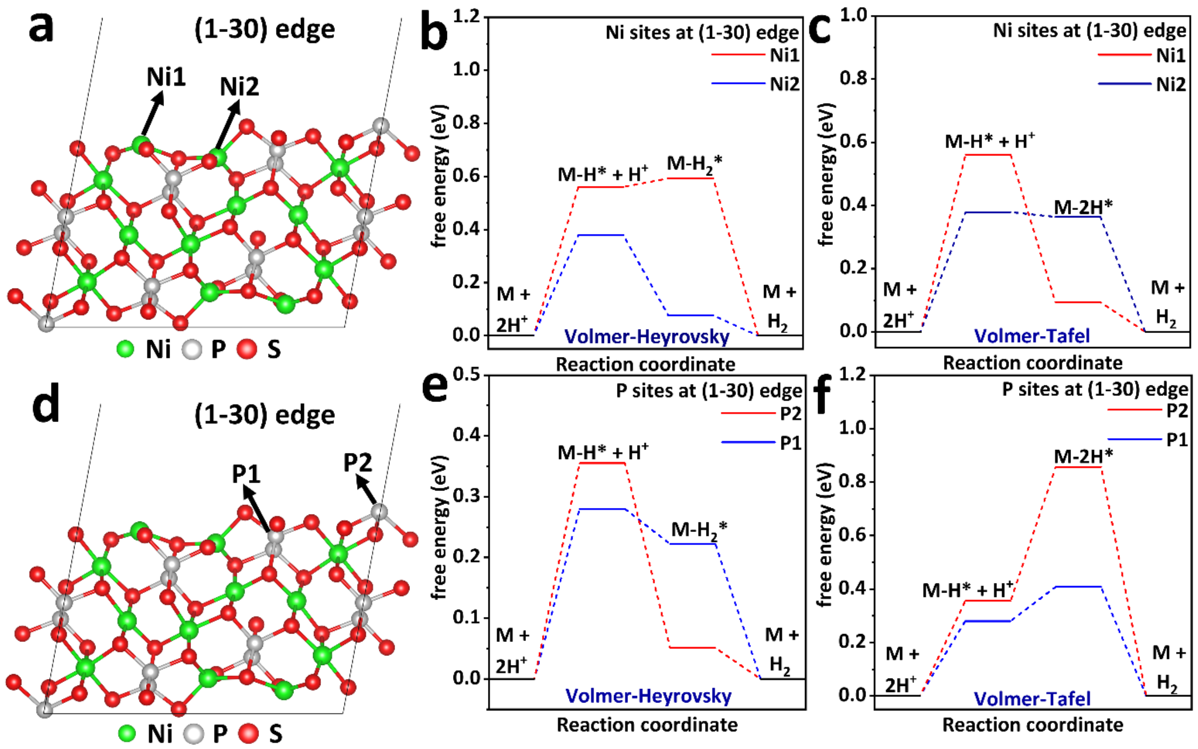

Supplementary Fig. 2. Ni and P sites calculated for (1-30) edge of $NiPS_3$ monolayer. (a) Ni1 and Ni2 sites calculated for hydrogen adsorption on (1-30) edge of $NiPS_3$ monolayer. Free energy diagrams for HER following the (b) Volmer-Heyrovsky pathway and (c) Volmer-Tafel pathway for Ni1 and Ni2 sites on (1-30) edge of $NiPS_3$ monolayer. (d) P1 and P2 sites calculated for hydrogen adsorption on (1-30) edge of $NiPS_3$ monolayer. Free energy diagrams for HER following the (e) Volmer-Heyrovsky pathway and (f) Volmer-Tafel pathway for P1 and P2 sites on (1-30) edge of $NiPS_3$ monolayer. All the Gibbs energy calculations were conducted considering the solvation effect in 17 vol% triethanolamine aqueous solution.

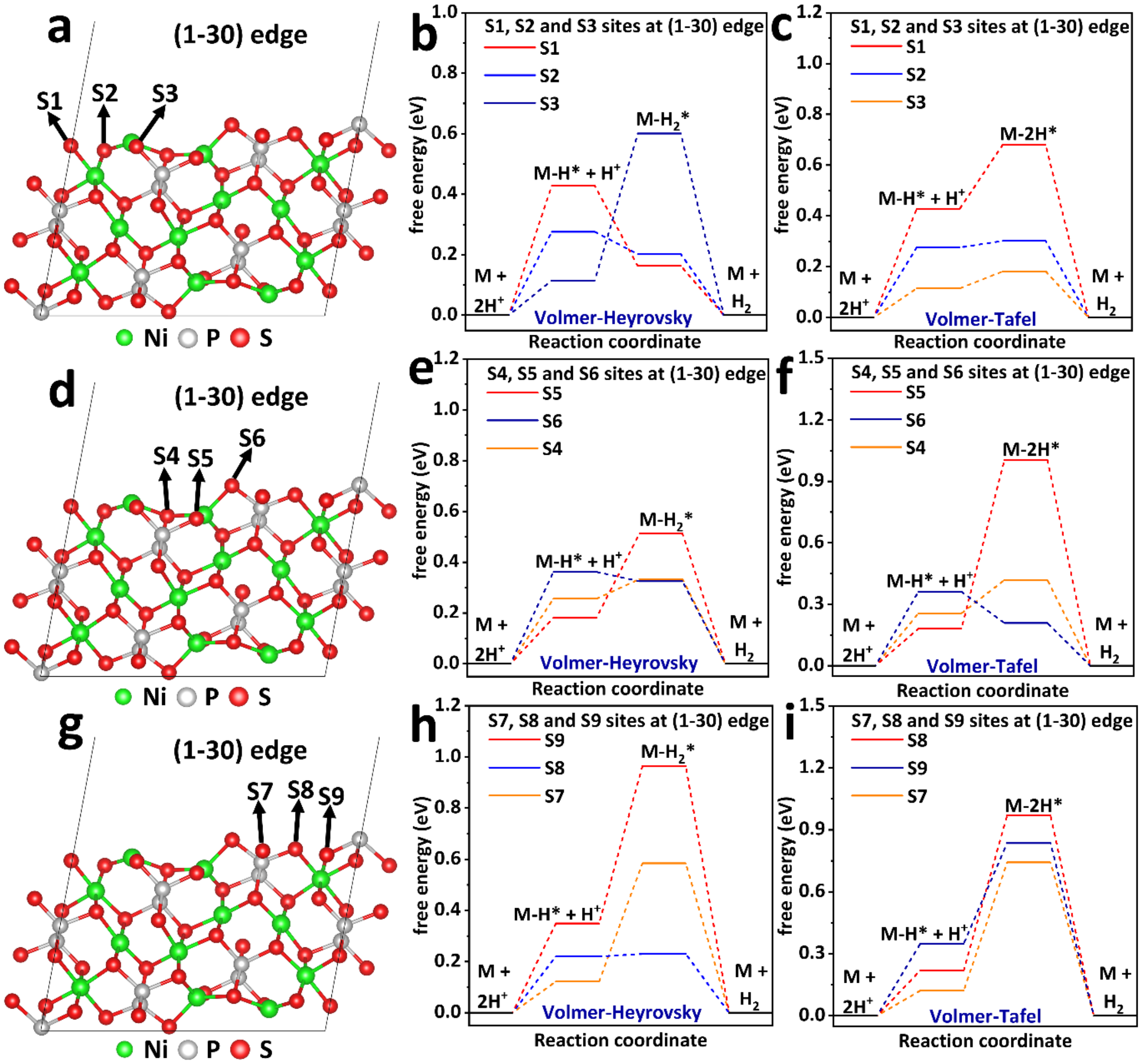

Supplementary Fig. 3. S sites calculated for (1-30) edge of $NiPS_3$ monolayer. (a) S1, S2 and S3 sites calculated for hydrogen adsorption on (1-30) edge of $NiPS_3$ monolayer. Free energy diagrams for HER following the (b) Volmer-Heyrovsky pathway and (c) Volmer-Tafel pathway for S1, S2 and S3 sites on (1-30) edge of $NiPS_3$ monolayer. (d) S4, S5 and S6 sites calculated for hydrogen adsorption on (1-30) edge of $NiPS_3$ monolayer. Free energy diagrams for HER following the (e) Volmer-Heyrovsky pathway and (f) Volmer-Tafel pathway for S4, S5 and S6 sites at (1-30) edge of $NiPS_3$ monolayer. (g) S7, S8 and S9 sites calculated for hydrogen adsorption on (1-30) edge of $NiPS_3$ monolayer. Free energy diagrams for HER following the (h) Volmer-Heyrovsky pathway and (i) Volmer-Tafel pathway for S7, S8 and S9 sites at (1-30) edge of $NiPS_3$ monolayer. All the Gibbs energy calculations were conducted considering the solvation effect in 17 vol% triethanolamine aqueous solution.

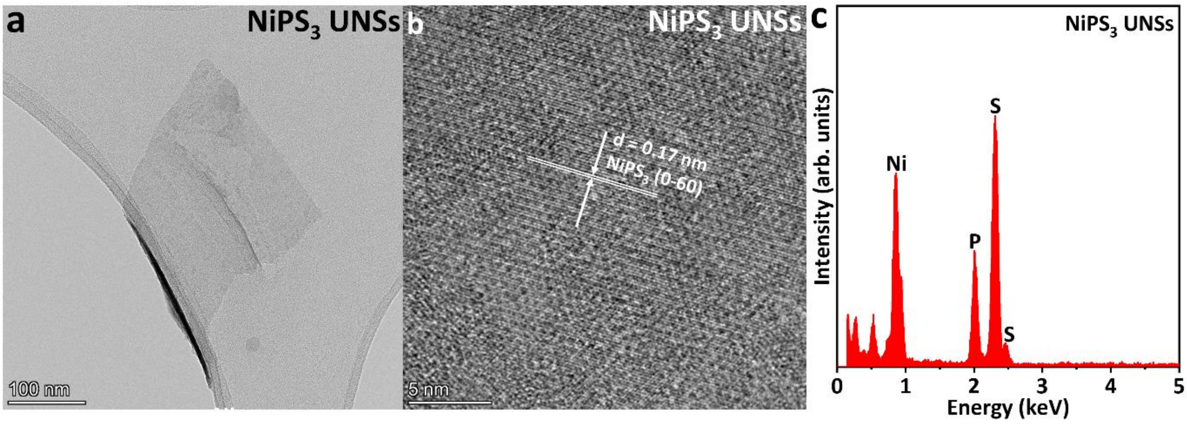

Supplementary Fig. 4. Morphology, microstructure and composition. (a) TEM image, (b) HRTEM image and (c) EDX spectrum of $NiPS_3$ UNSs.

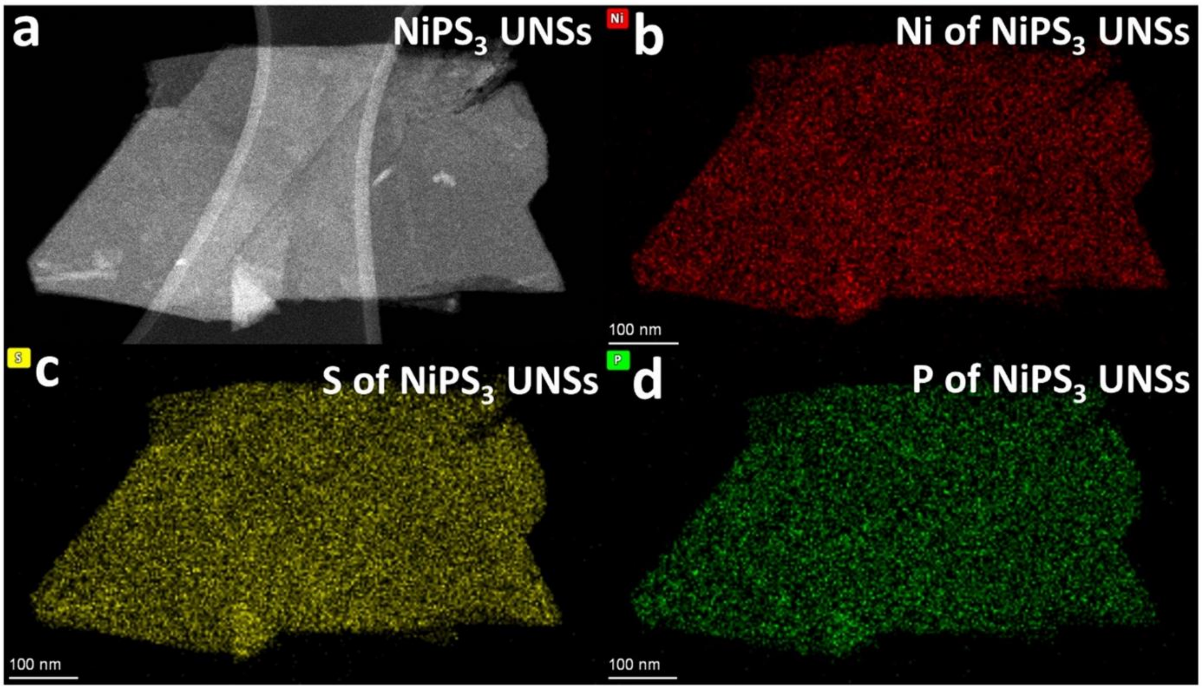

Supplementary Fig. 5. Morphology and composition. (a) HAADF-STEM image and the corresponding elemental mapping images of (b) Ni, (c) S and (d) P for $NiPS_3$ UNSs.

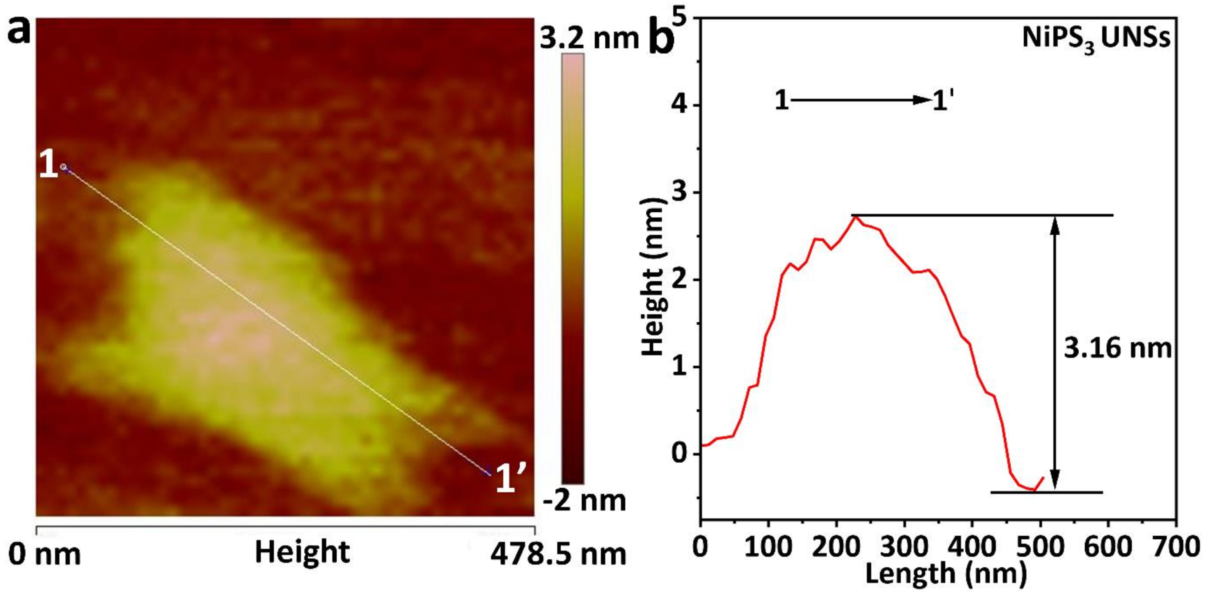

Supplementary Fig. 6. AFM characterization. (a) AFM image and (b) the corresponding height profile of $NiPS_3$ UNSs.

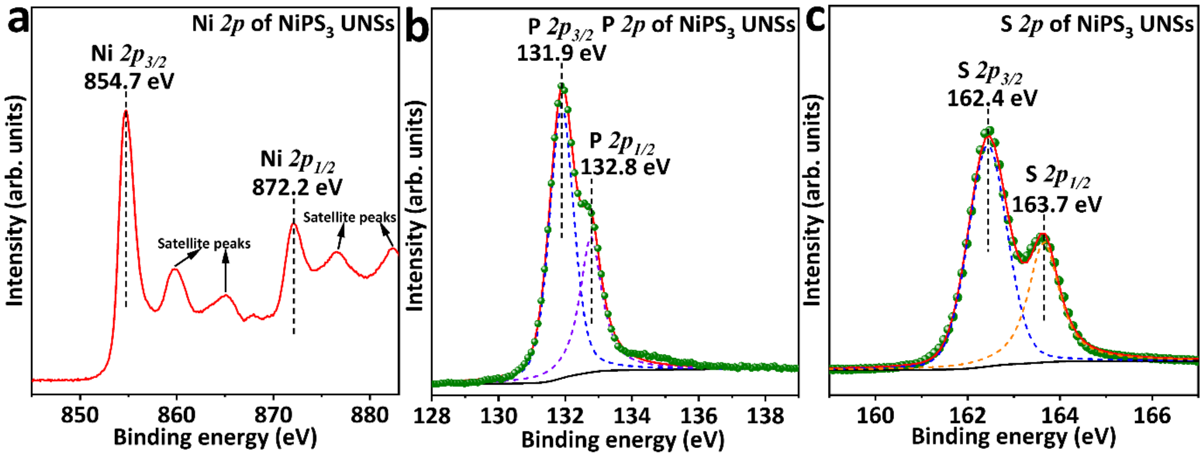

Supplementary Fig. 7. XPS characterizations. High-resolution XPS spectra of (a) Ni *2p*, (b) P *2p* and (c) S *2p* for $NiPS_3$ UNSs.

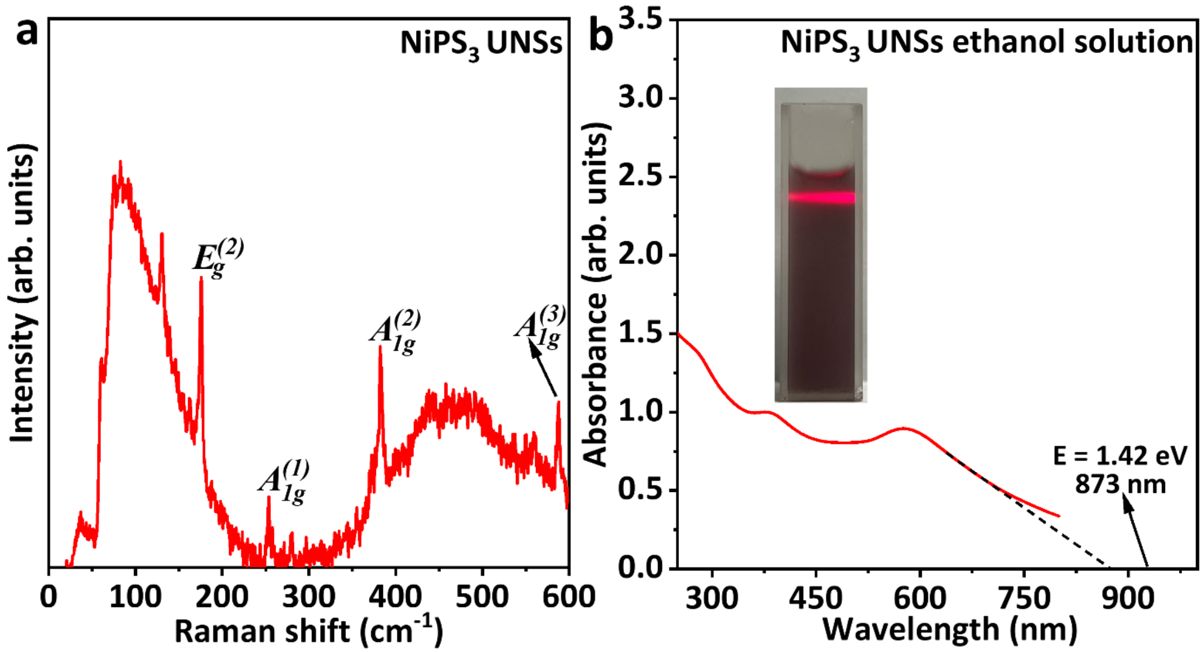

Supplementary Fig. 8. Raman and UV-Vis spectroscopy characterizations. (a) Raman spectroscopy of $NiPS_3$ UNSs. (b) UV-Vis absorption spectrum of $NiPS_3$ UNSs in ethanol. Supplementary Fig. 8b inset shows the color and Tyndall effect of $NiPS_3$ UNSs in ethanol.

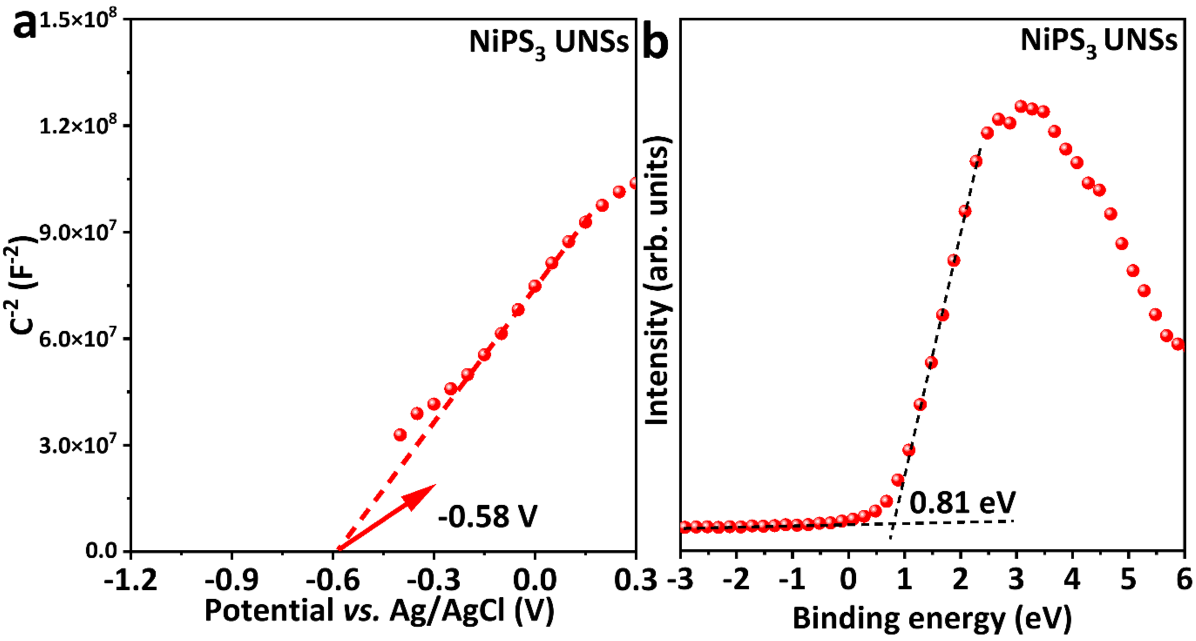

Supplementary Fig. 9. Band structure calculation. (a) Mott-Schottky plot of $NiPS_3$ UNSs in 0.5 M Na_2SO_4 aqueous solution. (b) XPS valence band spectrum of $NiPS_3$ UNSs.

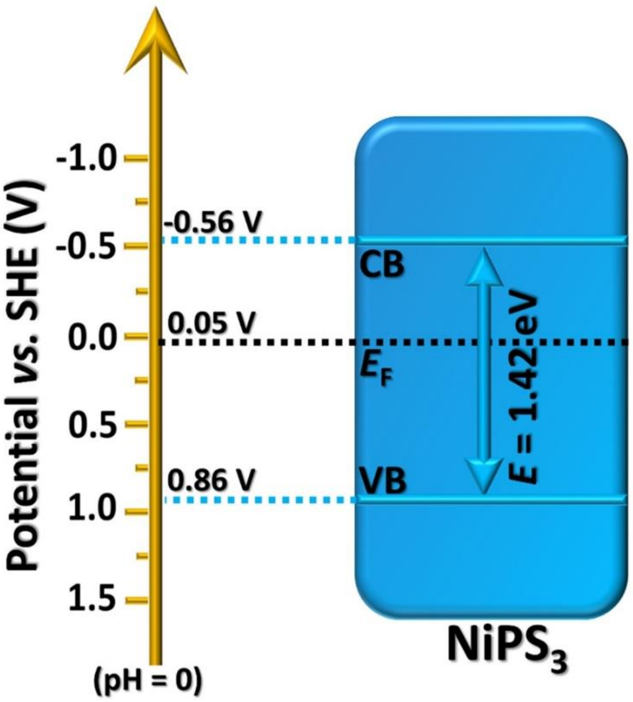

Supplementary Fig. 10. Graphic illustration. The electronic band structure for $NiPS_3$ UNSs.

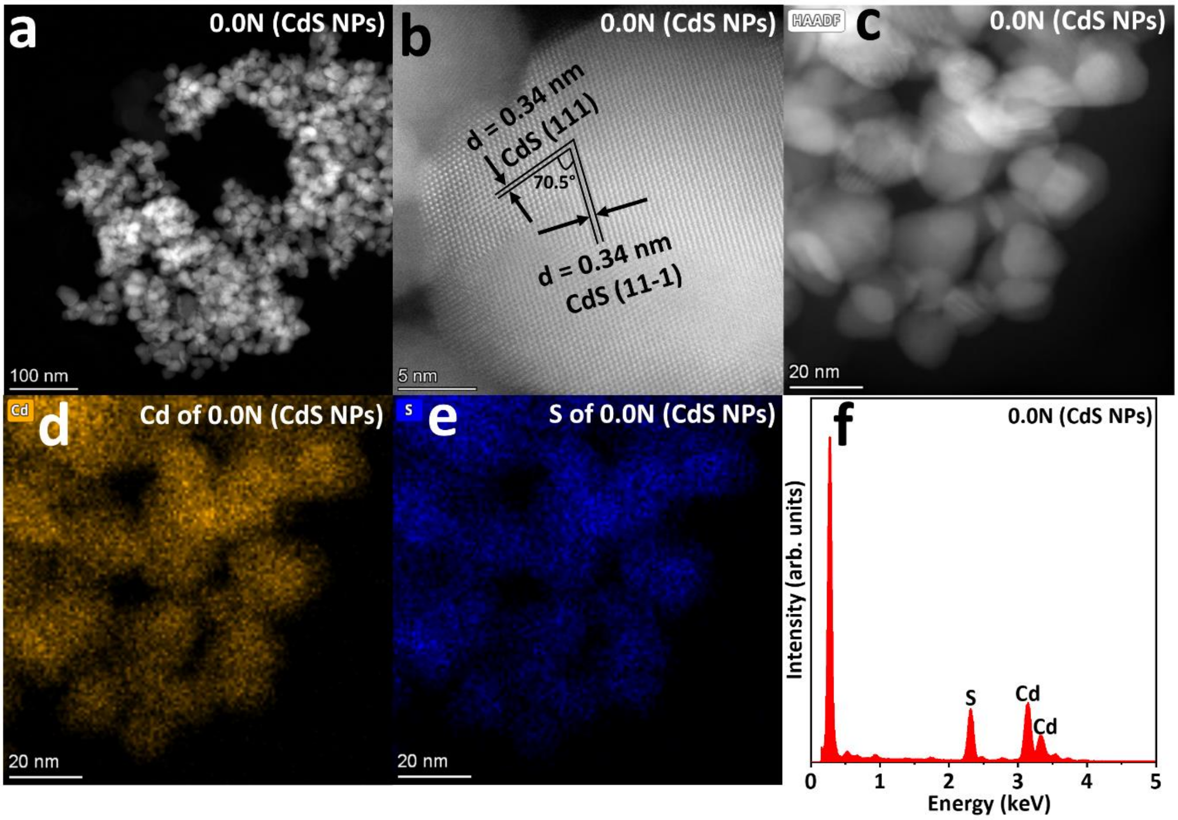

Supplementary Fig. 11. Morphology, microstructure and composition. (a) HAADF-STEM image and (b) high-resolution HAADF-STEM image of 0.0N (CdS NPs). (c) HAADF-STEM image of 0.0N (CdS NPs) and the corresponding elemental mapping images of (d) Cd and (e) S. (f) EDX spectrum of 0.0N (CdS NPs).

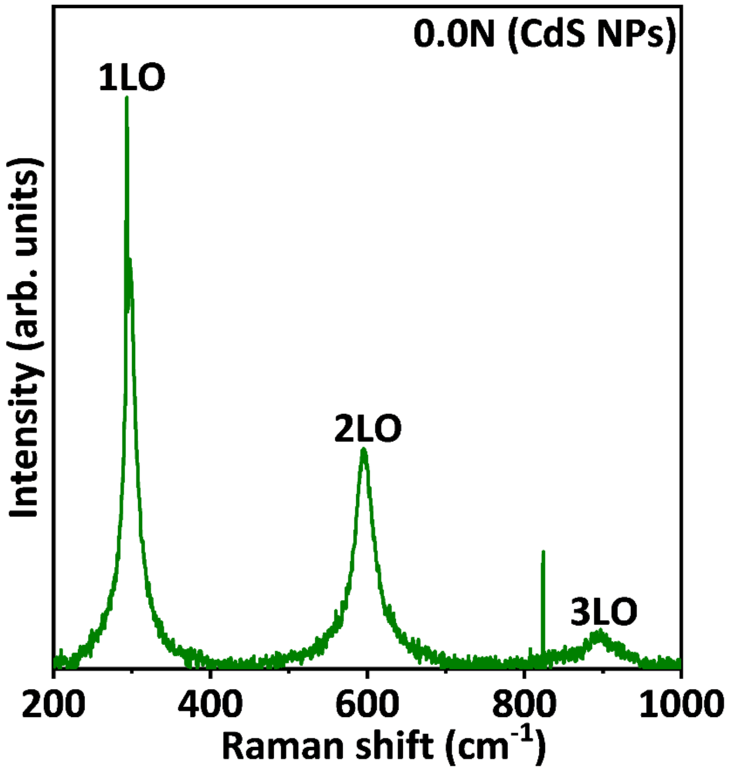

Supplementary Fig. 12. Raman characterization. Raman spectroscopy of 0.0N (CdS NPs).

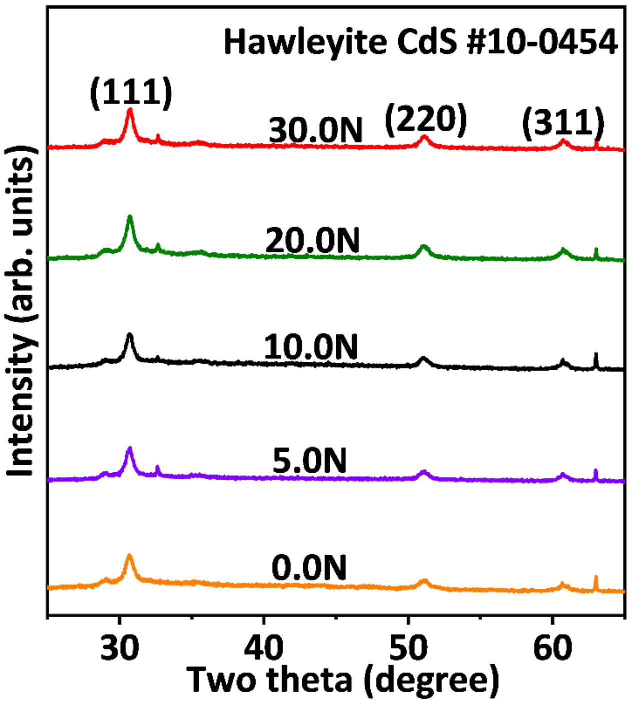

Supplementary Fig. 13. XRD results. XRD patterns of 0.0N, 5.0N, 10.0N, 20.0N and 30.0N.

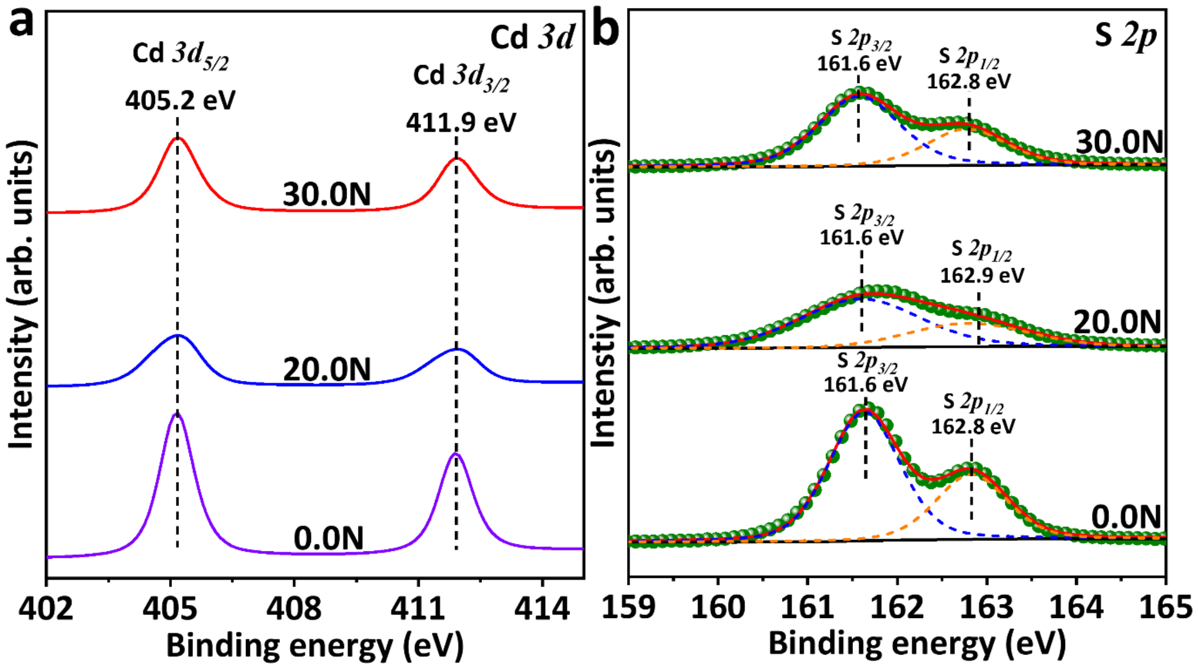

Supplementary Fig. 14. XPS characterizations. High-resolution XPS spectra of (a) Cd *3d* and (b) S *2p* for 0.0N, 20.0N and 30.0N.

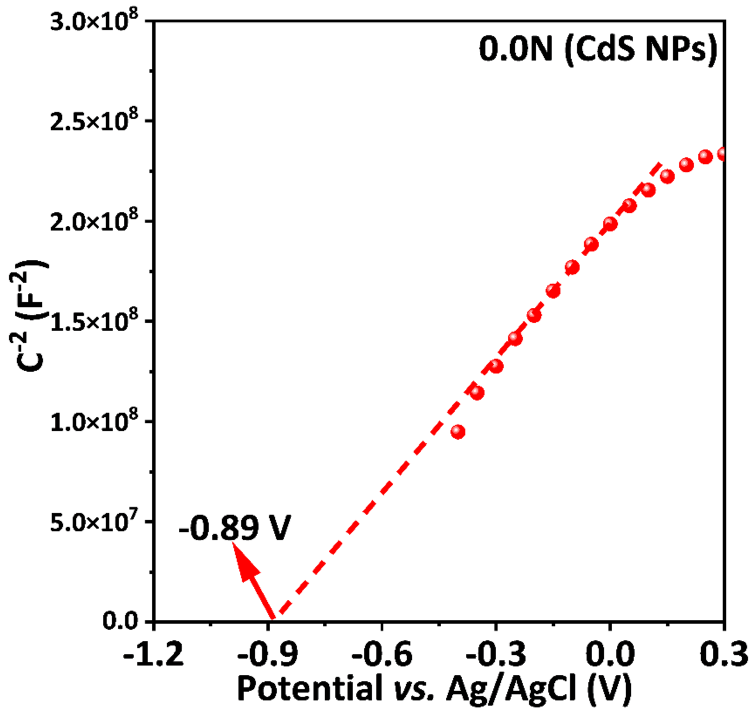

Supplementary Fig. 15. Mott-Schottky (MS) plot. MS plot of 0.0N (CdS NPs) in 0.5 M Na_2SO_4 aqueous solution.

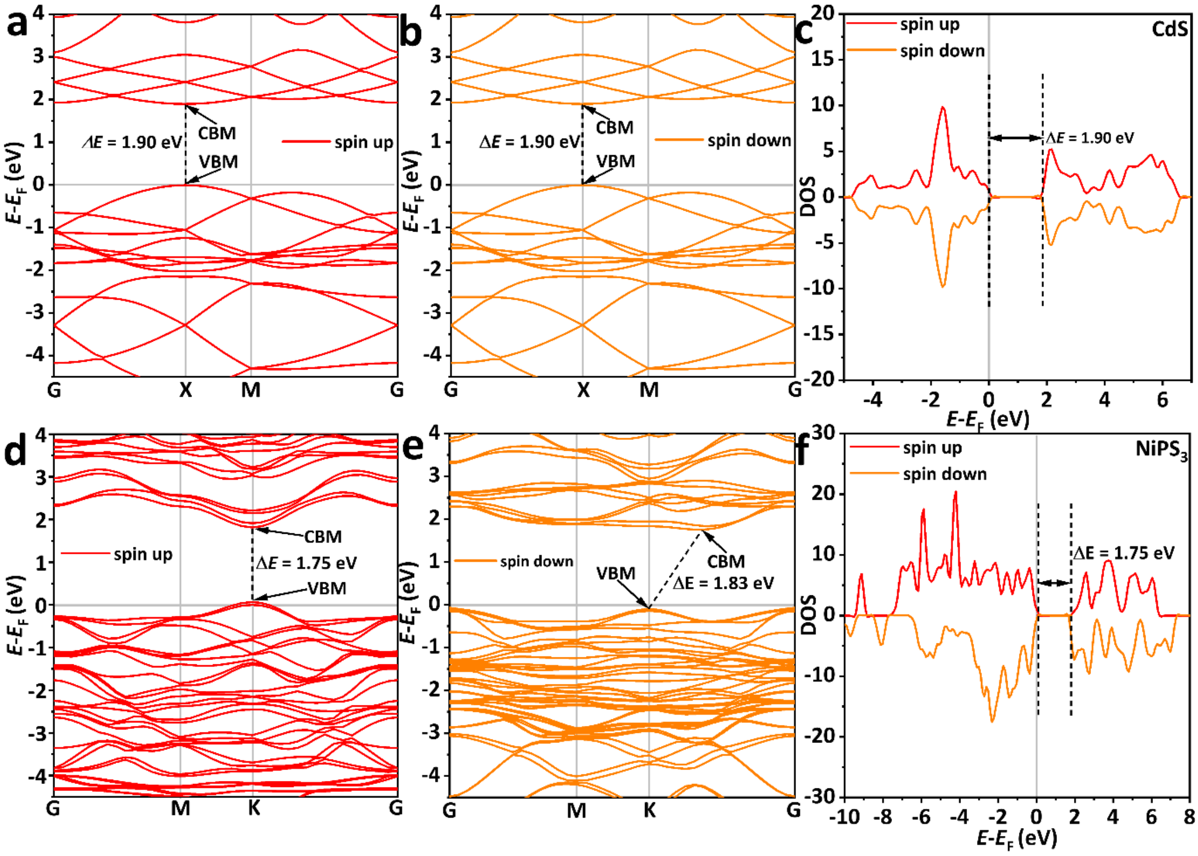

Supplementary Fig. 16. Calculated band structures and density of states (DOSs). (a) spin up and (b) spin down electronic band structures of CdS. (c) spin up and spin down DOS of CdS. (d) spin up and (e) spin down electronic band structures of $NiPS_3$. (f) spin up and spin down DOS of $NiPS_3$. The band structures and DOS were calculated considering the solvation effect in 17 vol% triethanolamine aqueous solution.

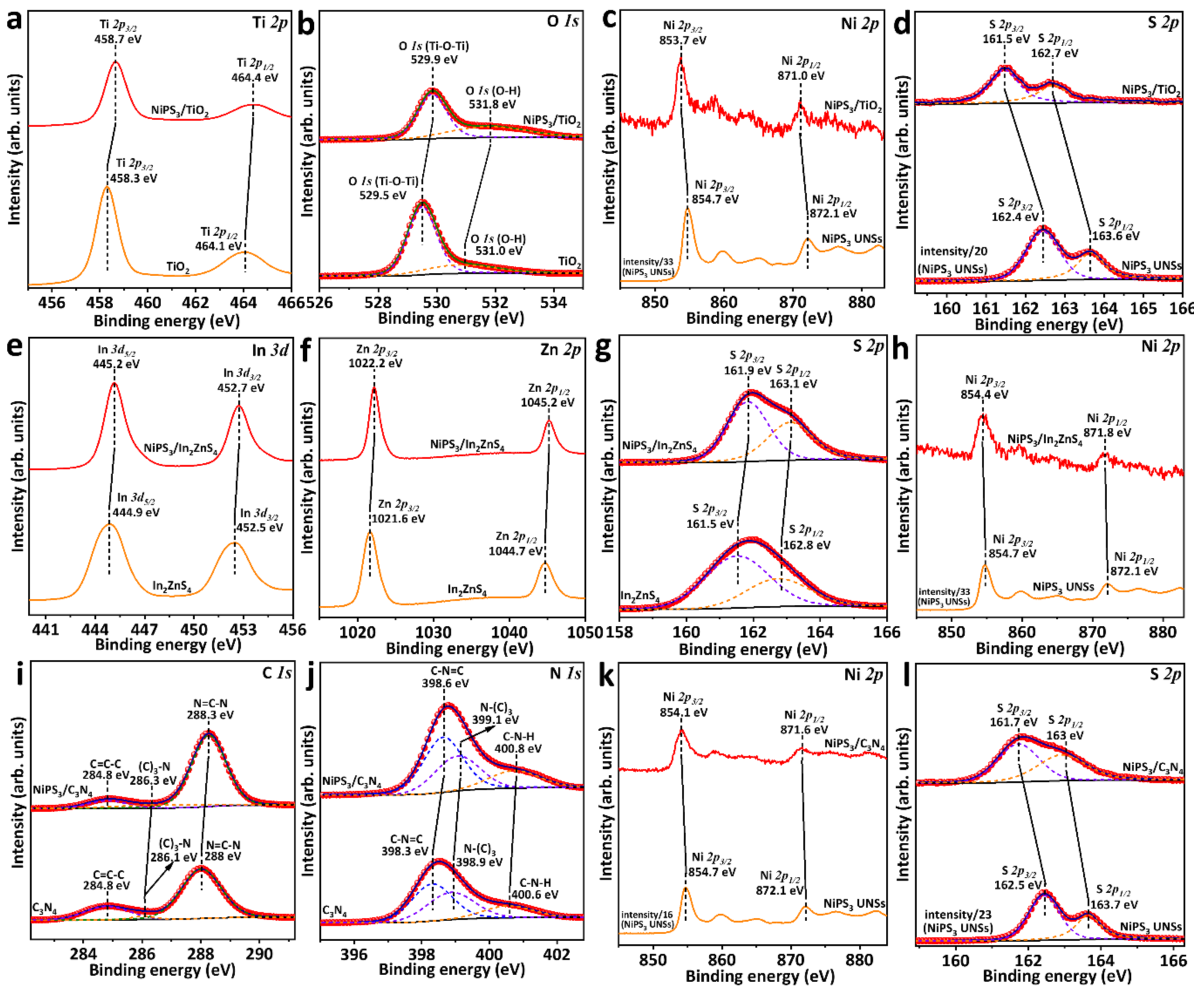

Supplementary Fig. 17. XPS characterizations. High-resolution XPS spectra of (a) Ti *2p* and (b) O *1s* for TiO_2 and $NiPS_3/TiO_2$. High-resolution spectra of (c) Ni *2p* (d) S *2p* for $NiPS_3$ UNSs and $NiPS_3/TiO_2$. High-resolution XPS spectra of (e) In *3d*, (f) Zn *2p* and (g) S *2p* for In_2ZnS_4 and $NiPS_3/In_2ZnS_4$. (h) High-resolution XPS spectra of Ni *2p* for $NiPS_3$ UNSs and $NiPS_3/In_2ZnS_4$. High-resolution XPS spectra of (i) C *1s* and (j) N *1s* of C_3N_4 and $NiPS_3/C_3N_4$. High-resolution XPS spectra of (k) Ni *2p* and (l) S *2p* of $NiPS_3$ UNSs and $NiPS_3/C_3N_4$.

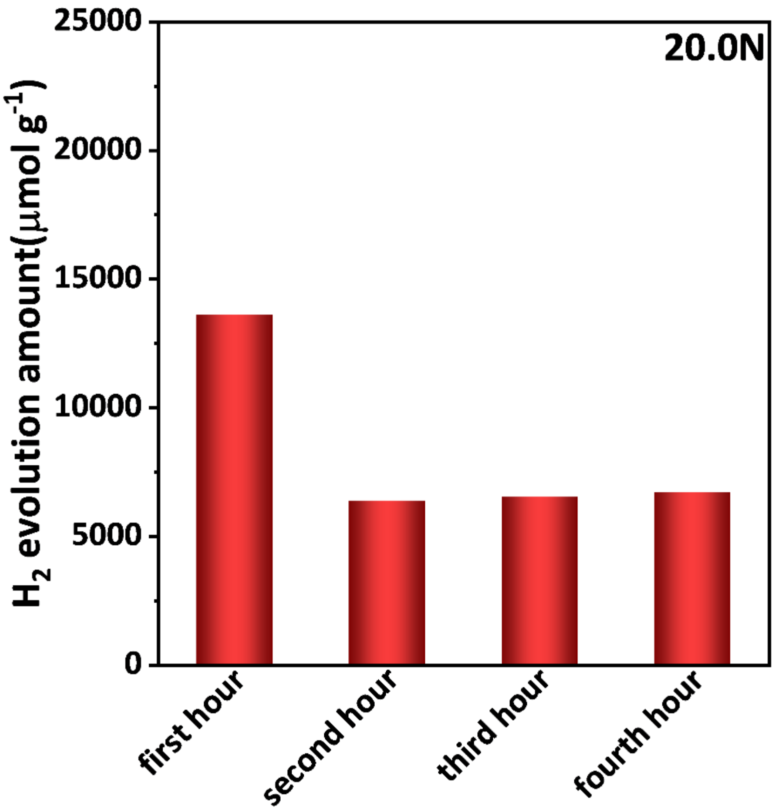

Supplementary Fig. 18. Photocatalytic stability. Photocatalytic H_2-production amount in the first, second, third and fourth hour in ~17 vol% triethanolamine aqueous solution under visible-light illumination (λ > 400 nm).

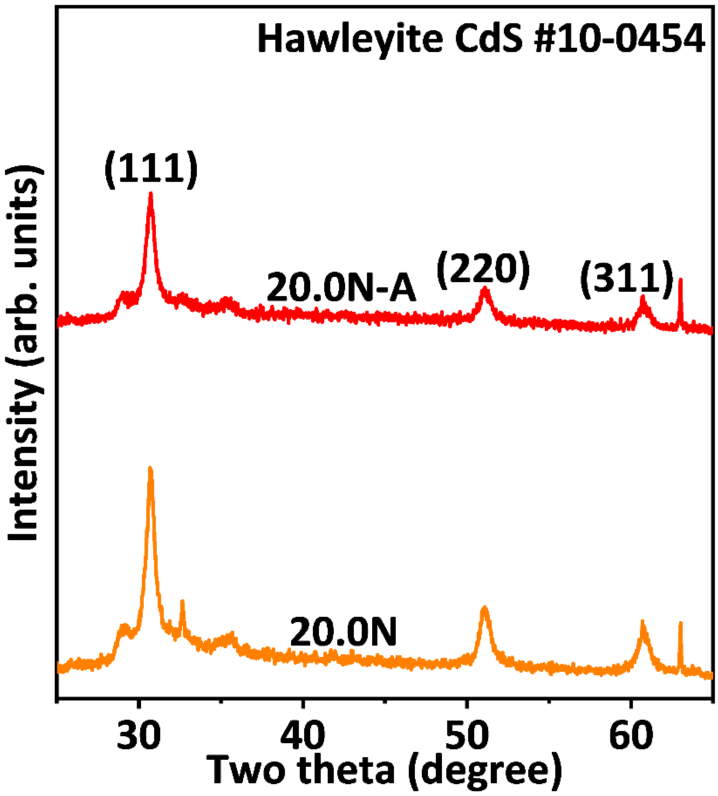

Supplementary Fig. 19. XRD results. XRD patterns of 20.0N and 20.0N-A.

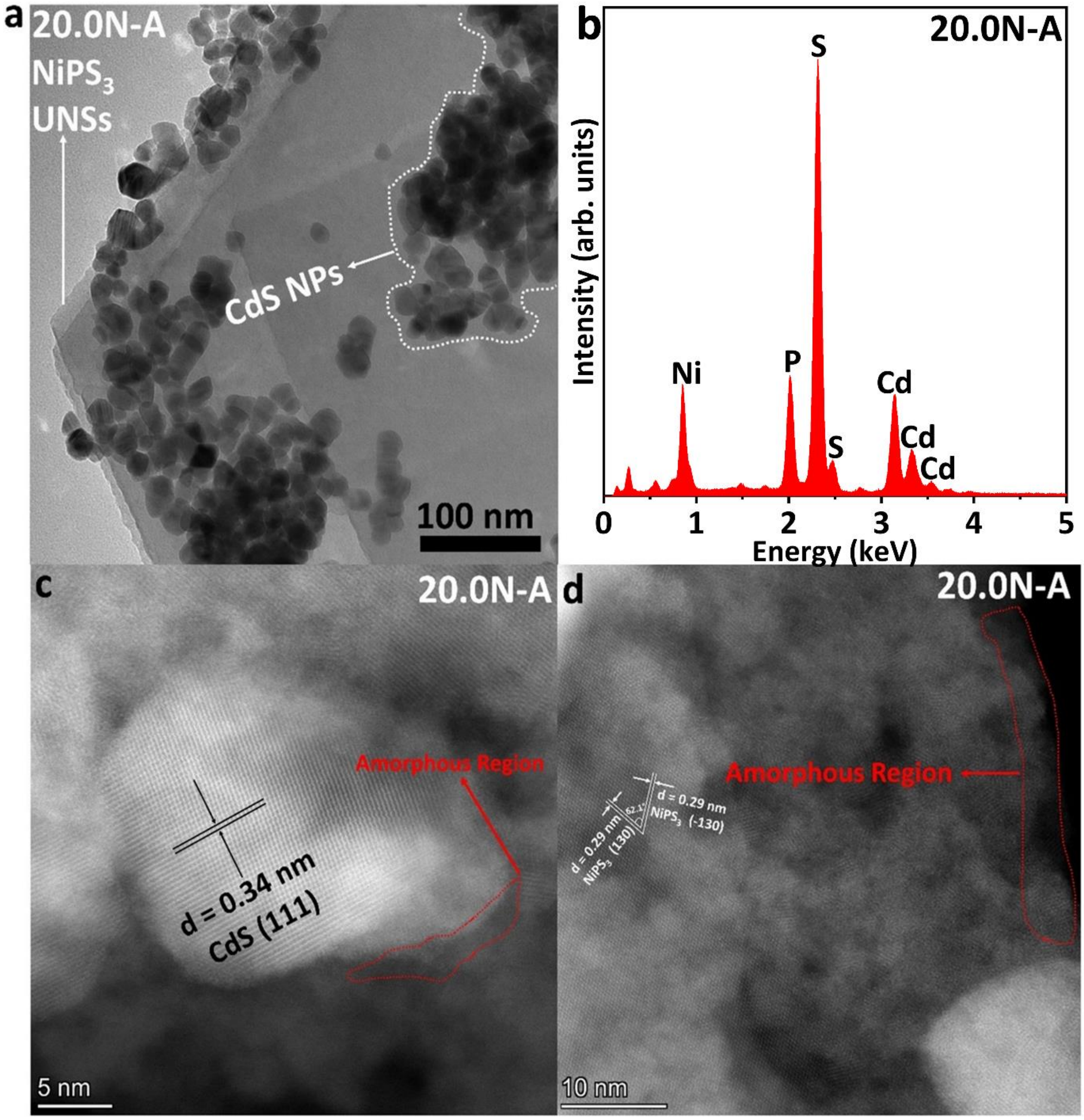

Supplementary Fig. 20. Morphology, microstructure and composition. (a) TEM image and (b) EDX spectrum of 20.0N-A. Atomic-resolution HAADF-STEM images showing the amorphous region at (c) CdS NPs and (d) $NiPS_3$ UNSs of 20.0N-A, which were generated after the four-hour photocatalytic H_2 production.

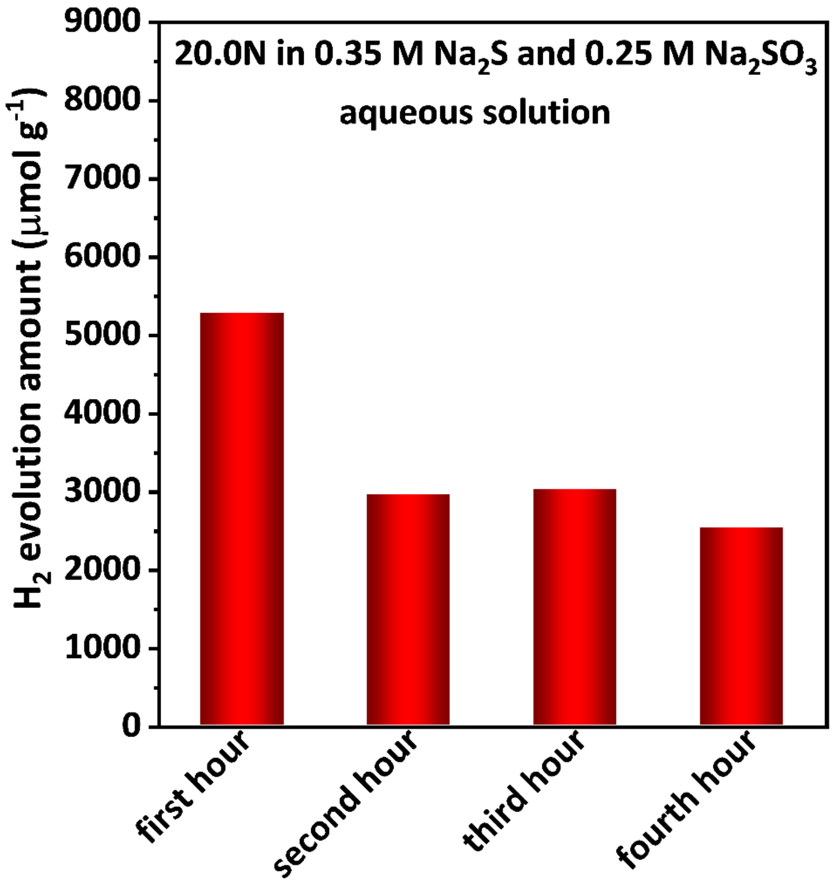

Supplementary Fig. 21. Photocatalytic stability. Photocatalytic H_2-production amounts in the first, second, third and fourth hour in 0.35 M Na_2S and 0.25 M Na_2SO_3 aqueous solution under visible-light illumination (λ > 400 nm).

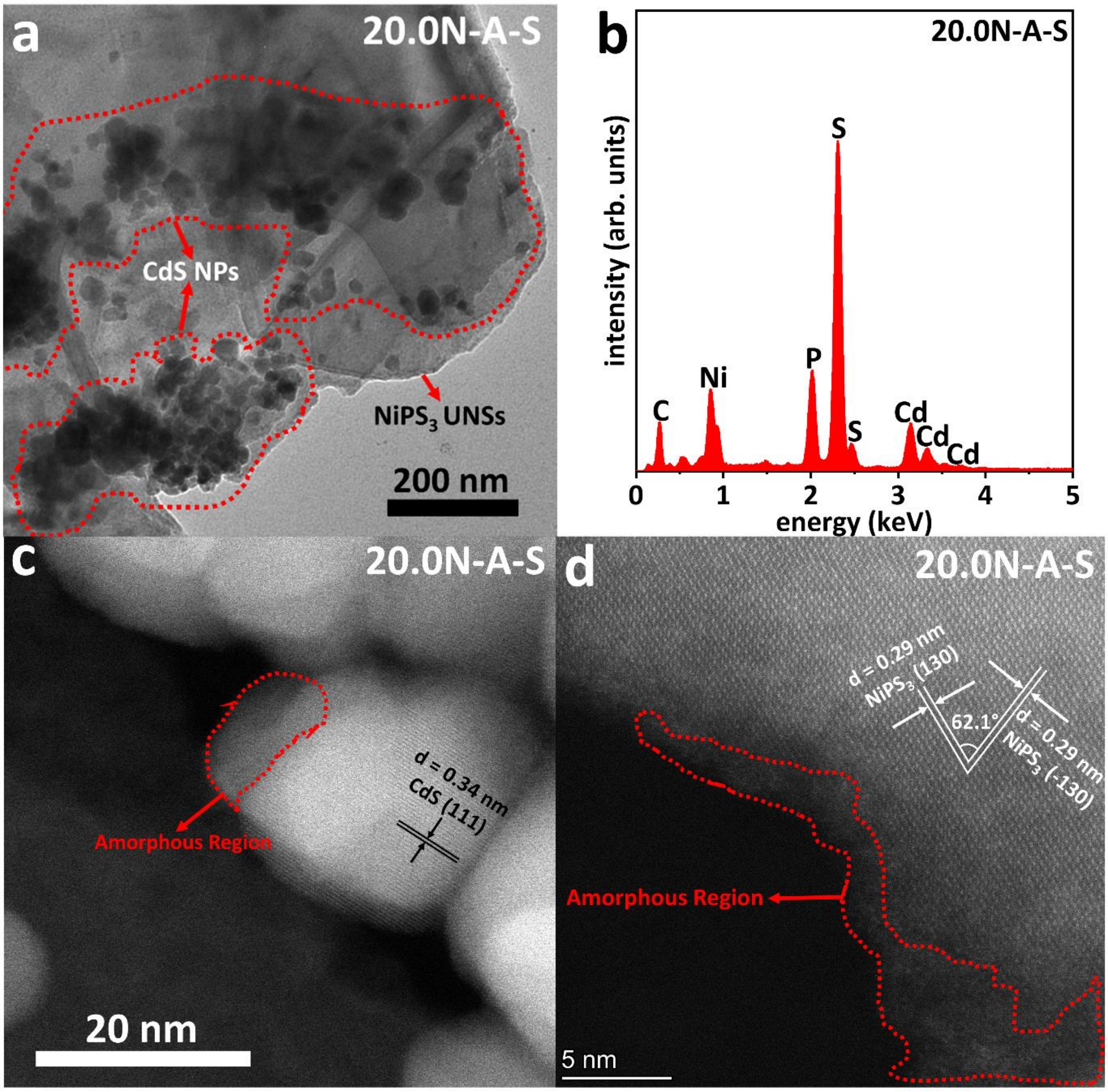

Supplementary Fig. 22. Morphology, microstructure and composition. (a) TEM image, (b) EDX spectrum and (c, d) atomic-resolution HAADF-STEM images of 20.0N-A-S.

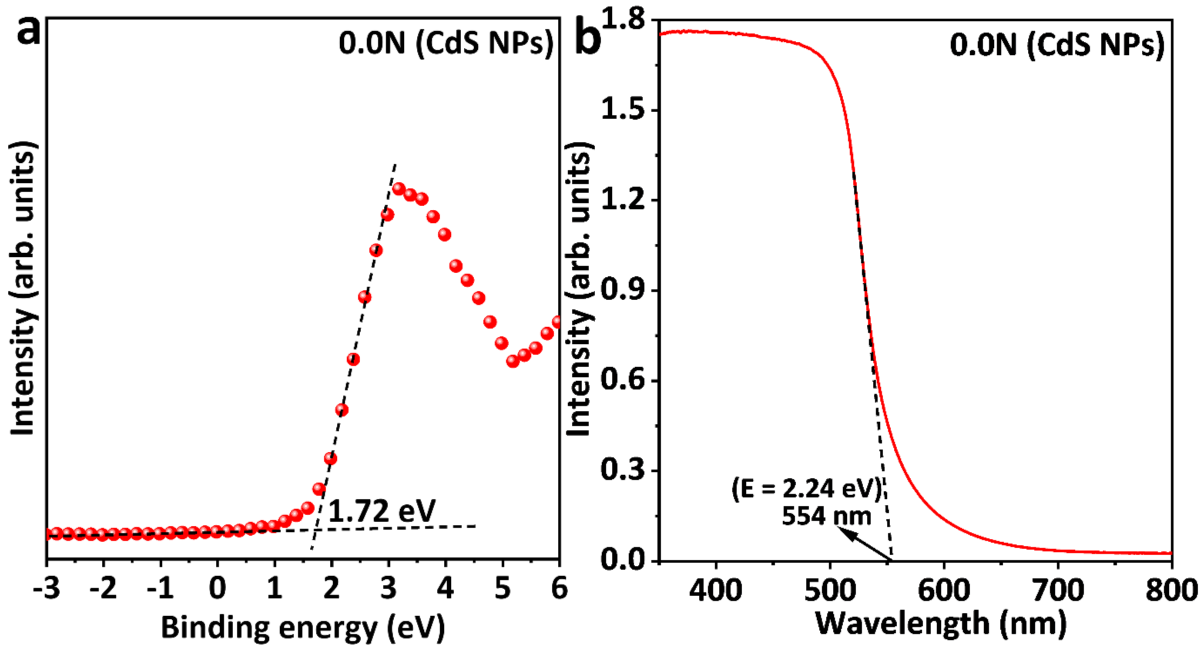

Supplementary Fig. 23. XPS and UV-Vis spectroscopy characterizations. (a) XPS valence band spectrum of 0.0N (CdS NPs). (b) UV-Vis diffuse reflectance spectrum of 0.0N (CdS NPs).

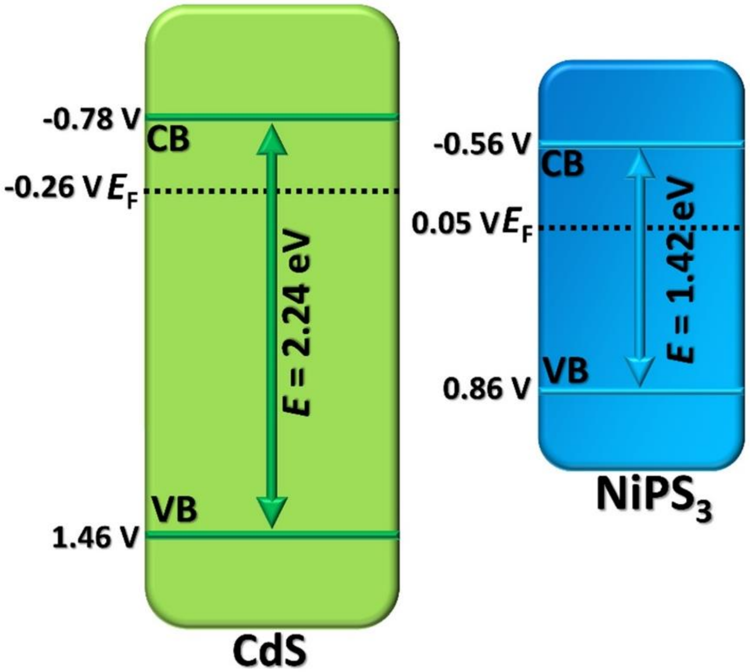

Supplementary Fig. 24. Schematic illustration. The electronic band structures for CdS NPs and $NiPS_3$ UNSs.

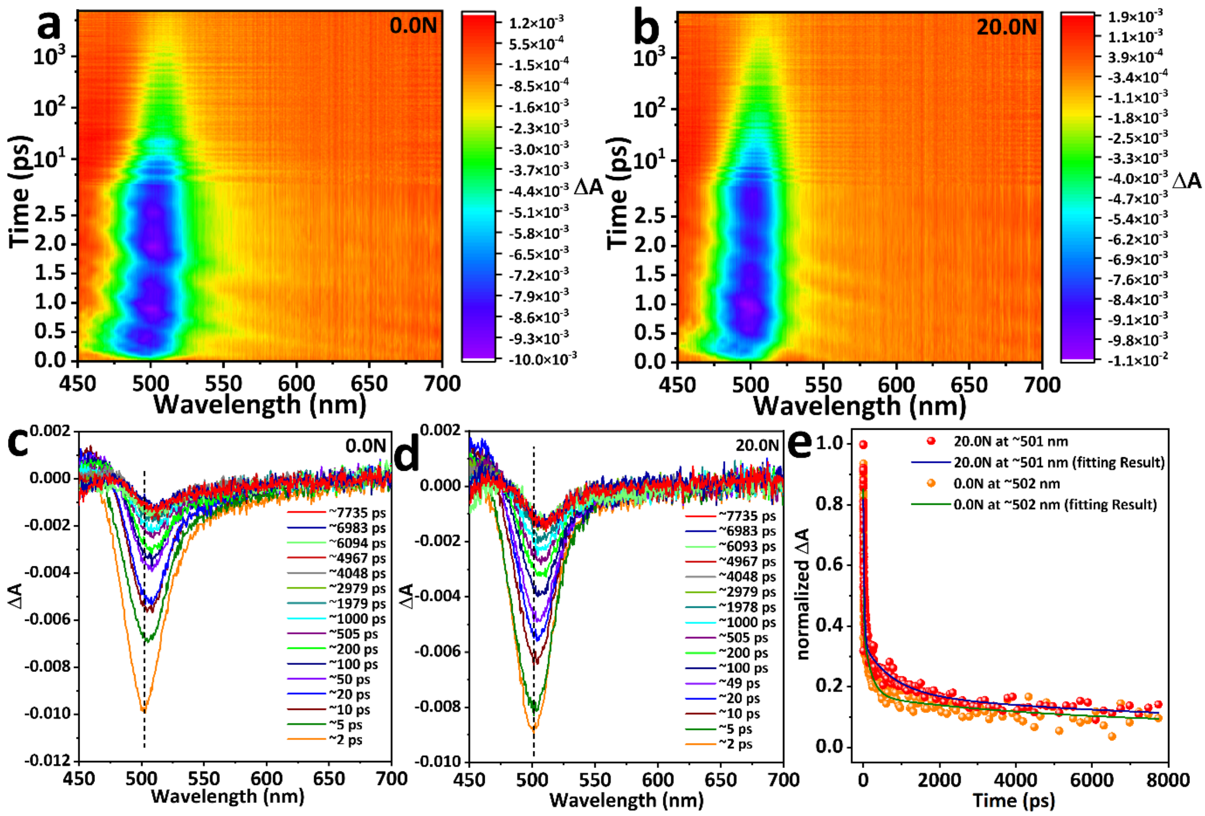

Supplementary Fig. 25. Transient absorption (TA) spectroscopy. 2D pseudocolor TA spectra of (a) 0.0N and (b) 20.0N in ethanol solution after the excitation by a 400 nm laser with a power of 120 μW. The TA spectra of (c) 0.0N and (d) 20.0N at different pump-probe delay time. (e) Normalized decay kinetics and fitting lines for 0.0N and 20.0N taken through the GSB peaks at ~502 and ~501 nm, respectively.

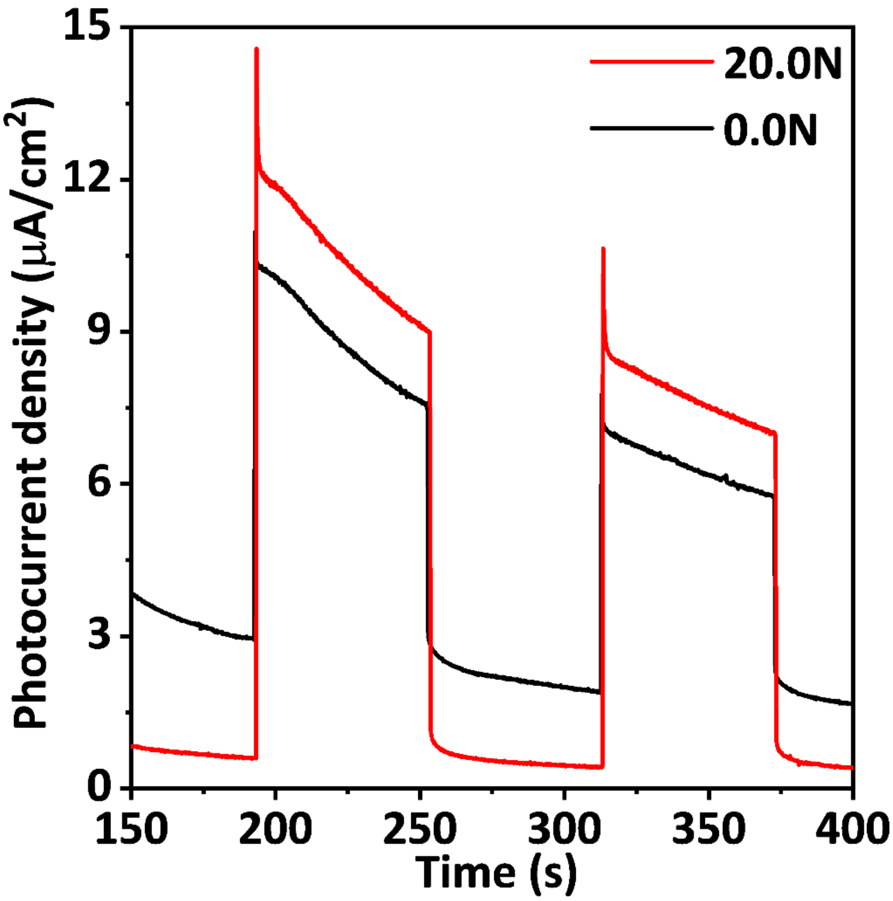

Supplementary Fig. 26. Transient photocurrent (TPC) density measurement. TPC density measurement of 0.0N and 20.0N electrodes in 0.5 M Na_2SO_4 aqueous solution.

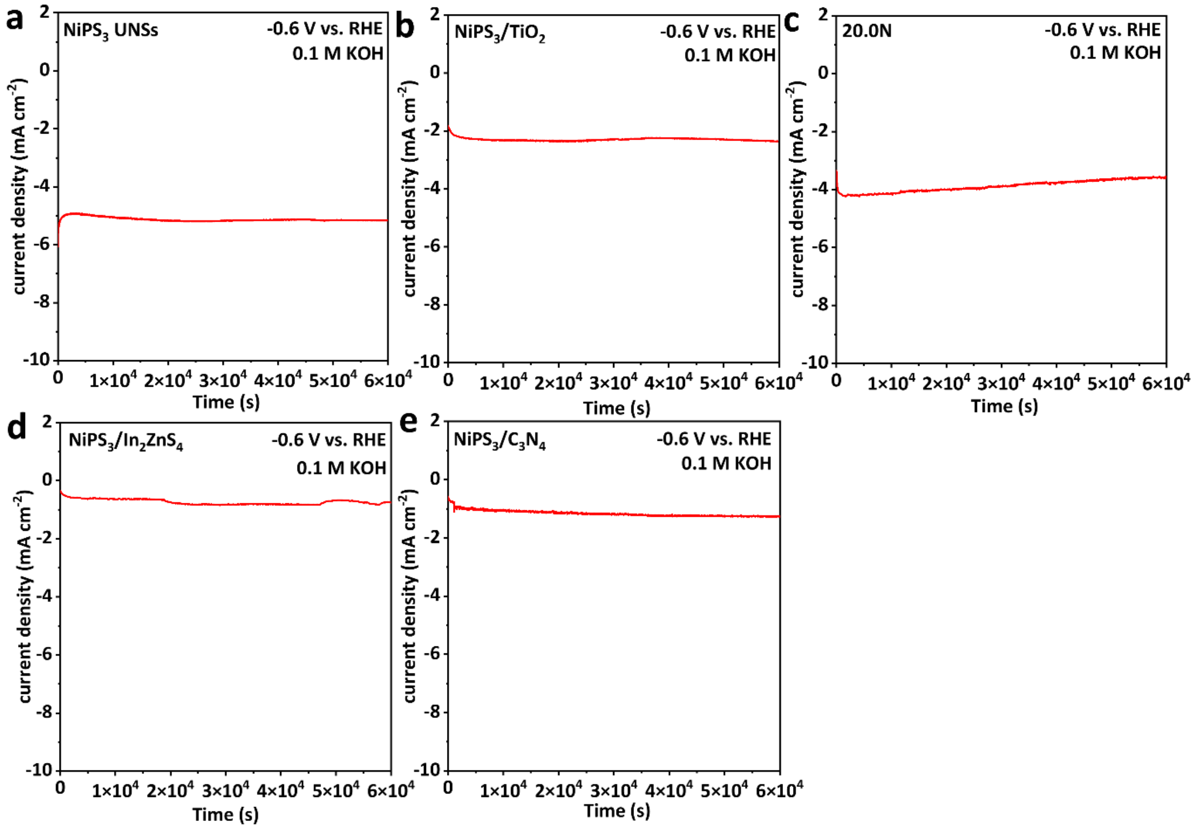

Supplementary Fig. 27. HER stability. HER electrochemical stability tests of (a) $NiPS_3$ UNSs, (b) $NiPS_3/TiO_2$, (c) 20.0N, (d) $NiPS_3/In_2ZnS_4$ and (e) $NiPS_3/C_3N_4$ loaded onto carbon paper at the potential of -0.6 V vs. RHE in 0.1 M KOH aqueous solution.

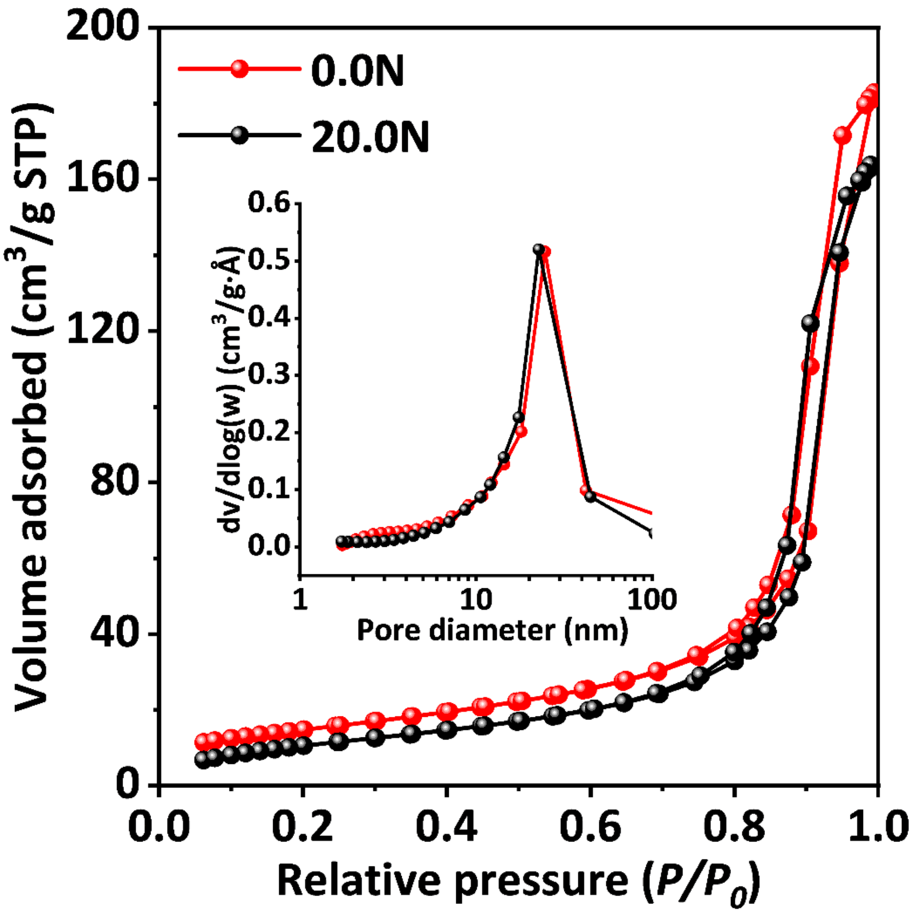

Supplementary Fig. 28. N_2 sorption analysis. N_2 sorption isotherms of 0.0N and 20.0N. Supplementary Fig. 28 inset shows the pore size distribution curves of 0.0N and 20.0N.

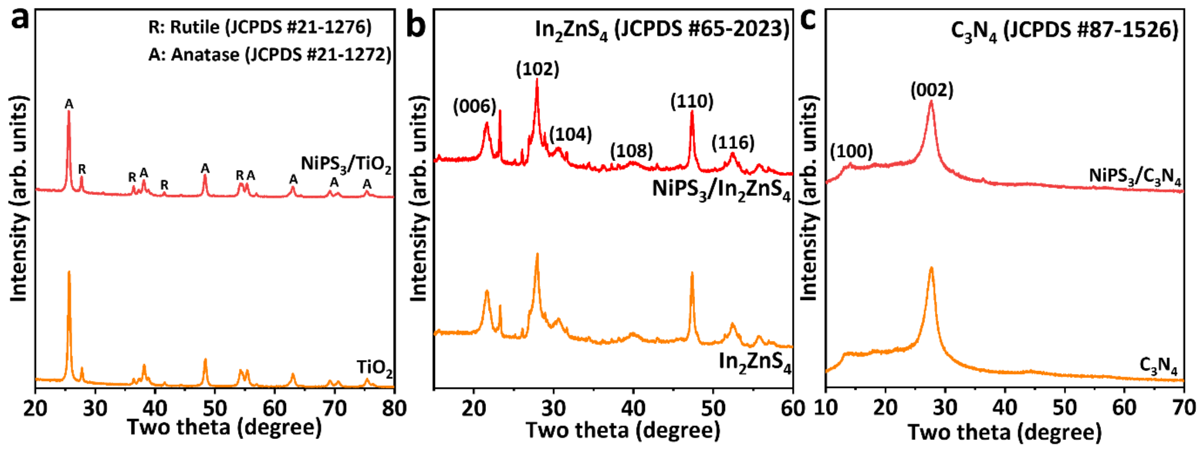

Supplementary Fig. 29. XRD results. XRD patterns of (a) TiO_2 and $NiPS_3/TiO_2$, (b) In_2ZnS_4 and $NiPS_3/In_2ZnS_4$ and (c) C_3N_4 and $NiPS_3/C_3N_4$.

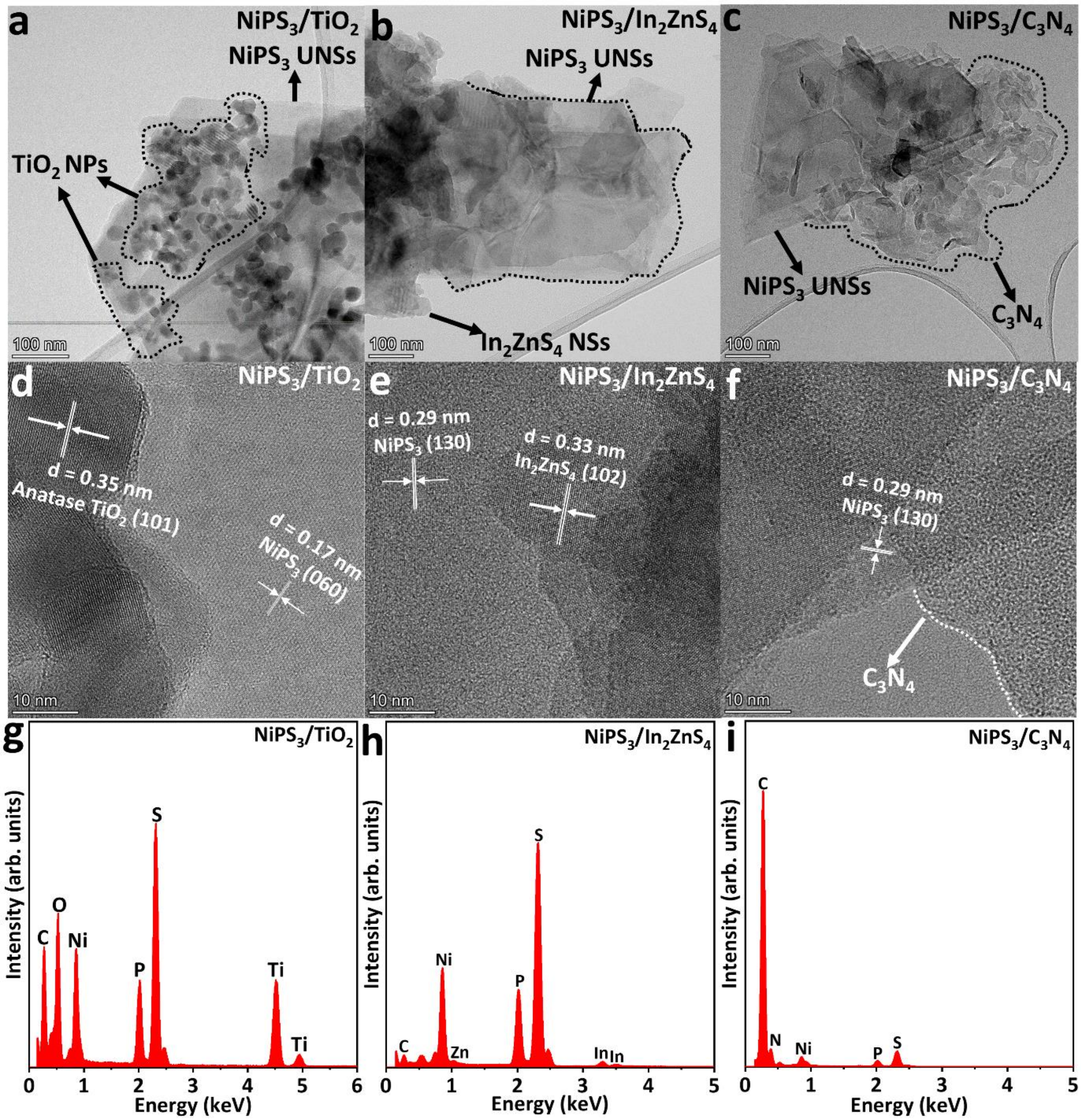

Supplementary Fig. 30. Morphology, microstructure and composition. TEM images of (a) $NiPS_3/TiO_2$, (b) $NiPS_3/In_2ZnS_4$ and (c) $NiPS_3/C_3N_4$. HRTEM images of (d) $NiPS_3/TiO_2$, (e) $NiPS_3/In_2ZnS_4$ and (f) $NiPS_3/C_3N_4$. EDX spectra of (g) $NiPS_3/TiO_2$, (h) $NiPS_3/In_2ZnS_4$ and (i) $NiPS_3/C_3N_4$.

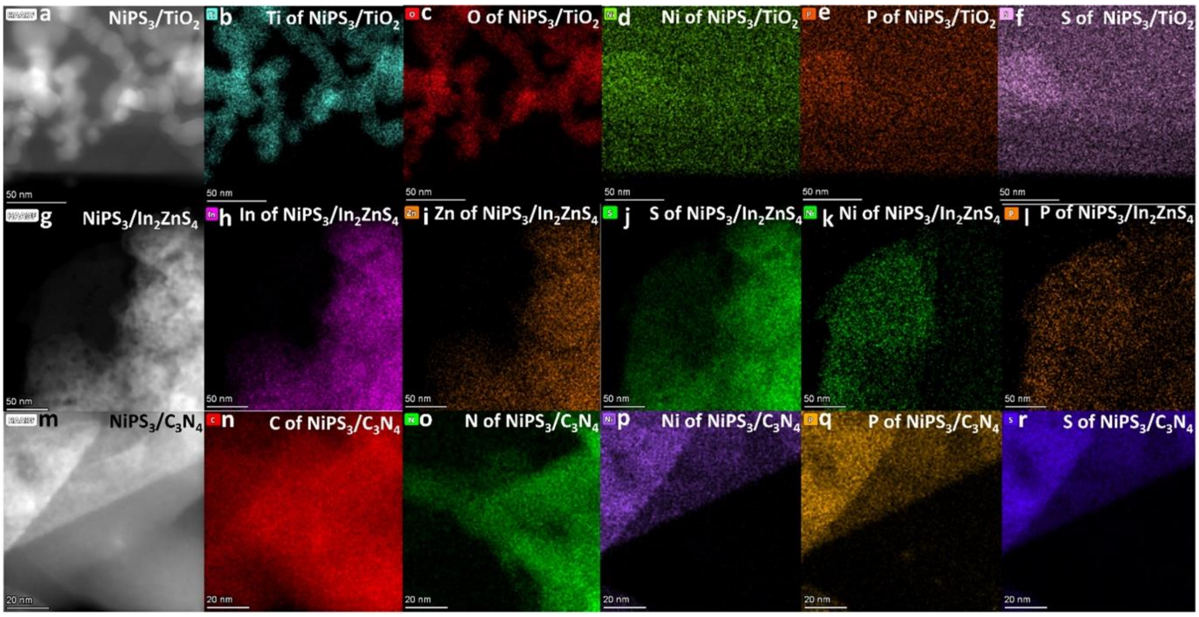

Supplementary Fig. 31. Morphology, microstructure and composition. (a) HAADF-STEM image of $NiPS_3/TiO_2$ and the corresponding elemental mapping images of (b) Ti, (c) O, (d) Ni, (e) P and (f) S. (g) HAADF-STEM image of $NiPS_3/In_2ZnS_4$ and the corresponding elemental mapping images of (h) In, (i) Zn, (j) S, (k) Ni and (l) P. (m) HAADF-STEM image of $NiPS_3/C_3N_4$ and the corresponding elemental mapping images of (n) C, (o) N, (p) Ni, (q) P and (r) S.

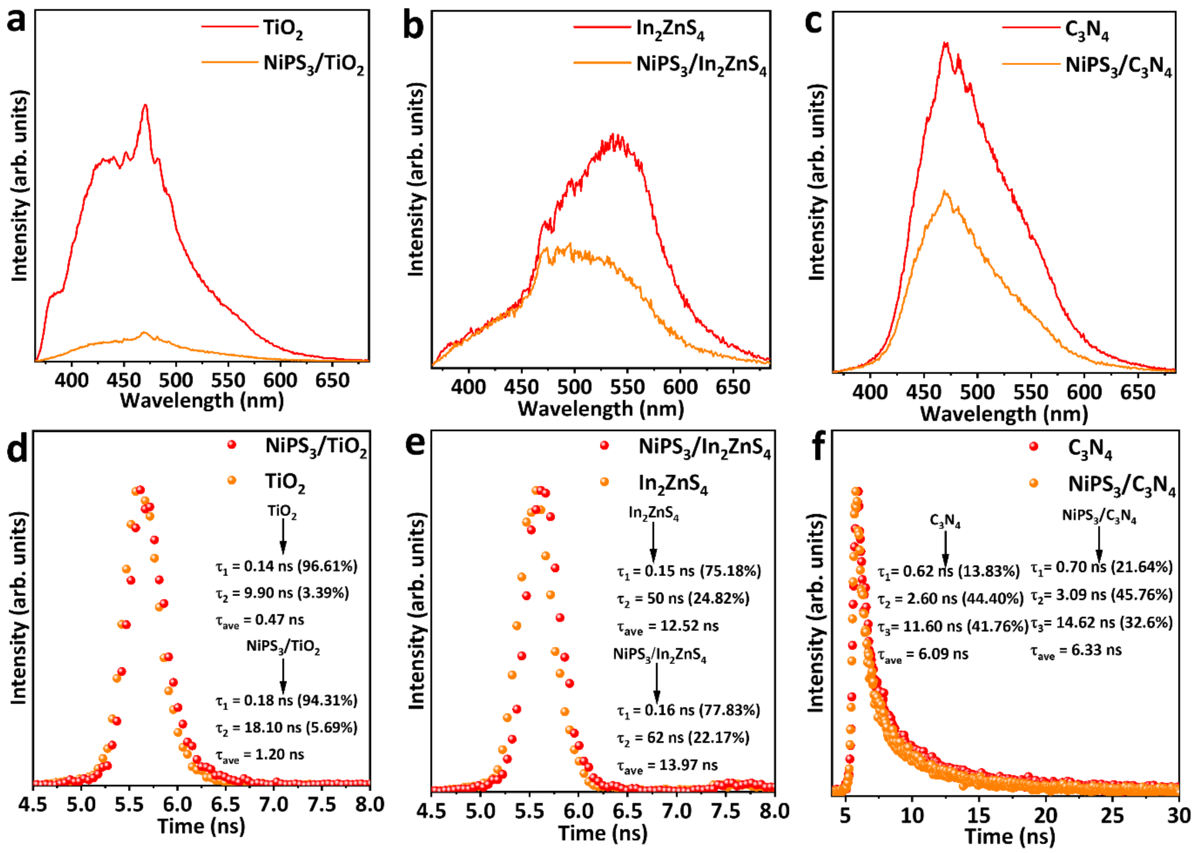

Supplementary Fig. 32. Photoluminescence spectroscopy characterizations. Steady-state photoluminescence spectra of (a) TiO_2 and $NiPS_3/TiO_2$, (b) In_2ZnS_4 and $NiPS_3/In_2ZnS_4$ and (c) C_3N_4 and $NiPS_3/C_3N_4$. Transient-state photoluminescence spectra of (d) TiO_2 and $NiPS_3/TiO_2$, (e) In_2ZnS_4 and $NiPS_3/In_2ZnS_4$ and (f) C_3N_4 and $NiPS_3/C_3N_4$. The insets in Supplementary Figs. 32d-f show the fitting and averaged charge carrier lifetimes.

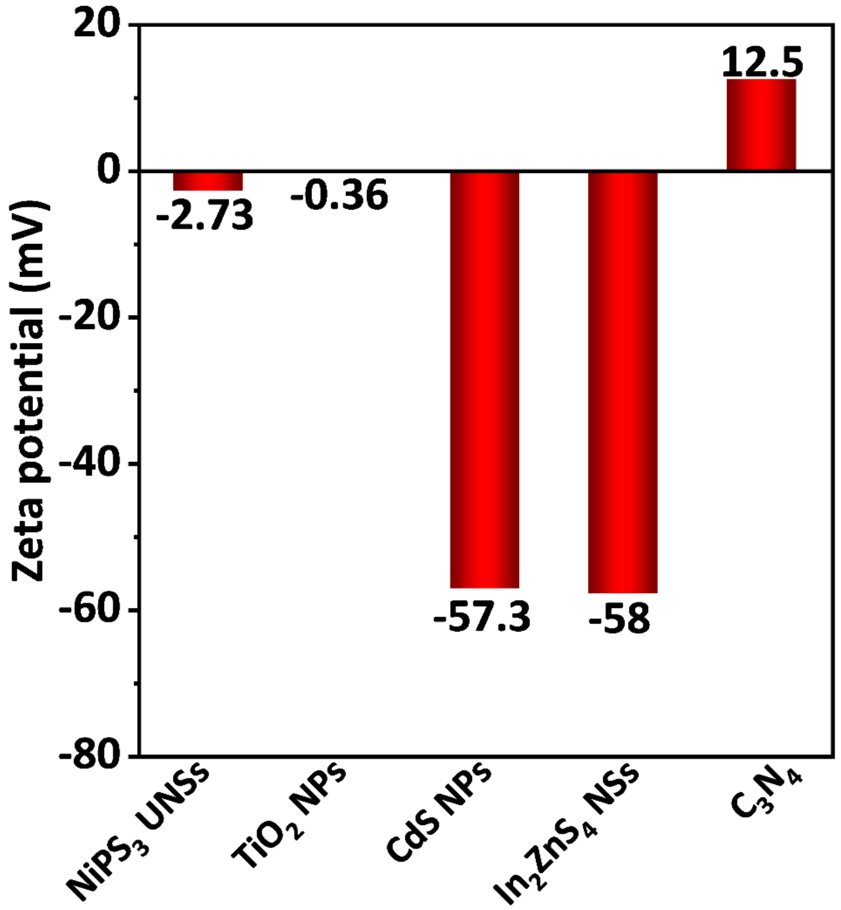

Supplementary Fig. 33. Zeta potentials analysis. Zeta potentials of $NiPS_3$ UNSs, TiO_2 NPs, CdS NPs, In_2ZnS_4 NSs and C_3N_4 in ethanol.

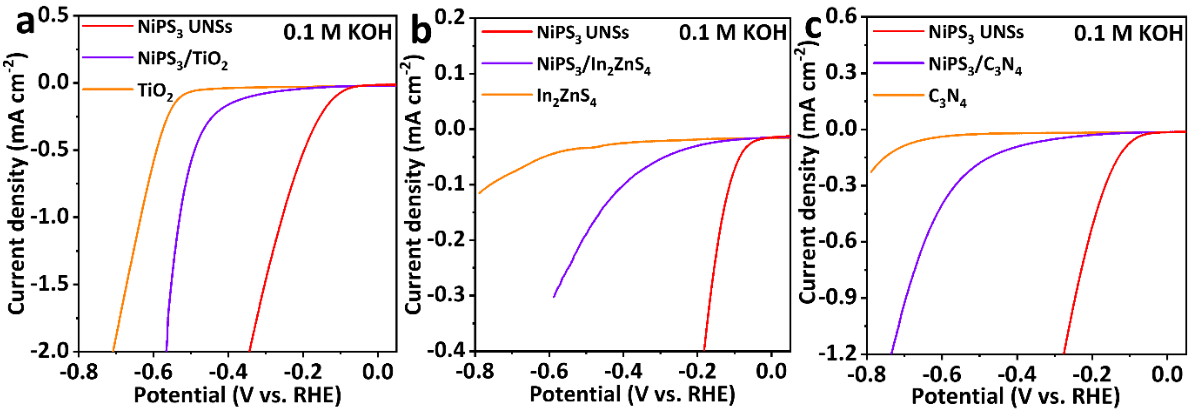

Supplementary Fig. 34. HER activities. HER activities of (a) $NiPS_3$ UNSs, $NiPS_3/TiO_2$ and TiO_2, (b) $NiPS_3$ UNSs, $NiPS_3/In_2ZnS_4$ and In_2ZnS_4, and (c) $NiPS_3$ UNSs, $NiPS_3/C_3N_4$ and C_3N_4 in 0.1 M KOH aqueous solution.

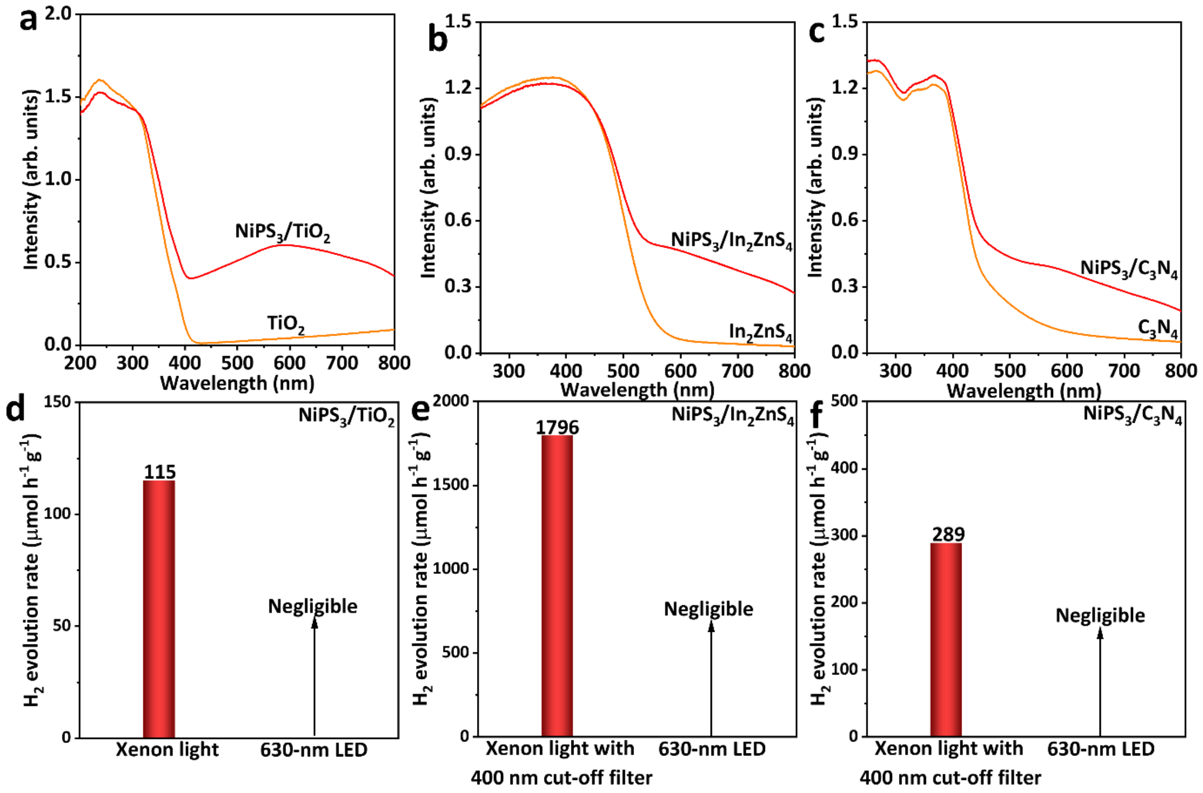

Supplementary Fig. 35. Optical properties and photocatalytic activities. UV-Vis diffuse reflectance spectra of (a) TiO_2 and $NiPS_3/TiO_2$, (b) In_2ZnS_4 and $NiPS_3/In_2ZnS_4$ and (c) C_3N_4 and $NiPS_3/C_3N_4$. (d) Photocatalytic H_2-production rates on $NiPS_3/TiO_2$ in ~17 vol% triethanolamine aqueous solution under xenon light irradiation and 630-nm LED, respectively. (e) Photocatalytic H_2-production rates on $NiPS_3/In_2ZnS_4$ in ~17 vol% triethanolamine aqueous solution under xenon light irradiation (λ > 400 nm) and 630-nm LED, respectively. (f) Photocatalytic H_2-production rates on $NiPS_3/C_3N_4$ in ~17 vol% triethanolamine aqueous solution under xenon light irradiation (λ > 400 nm) and 630-nm LED, respectively.

Supplementary Table 1. Reported activities for electrocatalytic HER on the MPC_x (M = Cr, Mn, Fe, Co, Ni, Zn, Ga, Cd, Sn and Bi; C = S and Se) catalysts.

MPC_x	overpotentials in Acid solution (j = -10 mA cm^{-2})	overpotentials in Alkaline solution (j = -10 mA cm^{-2})	References
$CrPS_4$	890 mV < < 1200 mV in 0.5 M H_2SO_4	N/A	1
$MnPS_3$	835 mV in 0.5 M H_2SO_4	1090 mV in 1.0 M KOH	2
$MnPS_3$	N/A	1140 mV in 1.0 M KOH	3
$MnPSe_3$	640 mV in 0.5 M H_2SO_4	992 mV in 1.0 M KOH	2
$FePS_3$	211 mV in 0.5 M H_2SO_4	337 mV in 0.5 M KOH	4
$FePS_3$	241 mV in 0.5 M H_2SO_4	N/A	5
$FePS_3$	429 mV in 0.5 M H_2SO_4	N/A	6
$FePSe_3$	~800 mV in 0.5 M H_2SO_4	~910 mV in 1.0 M KOH	7
$FePSe_3$	818 mV in 0.5 M H_2SO_4	N/A	8
$CoPS_3$	590 mV in 0.5 M H_2SO_4	N/A	1
$NiPS_3$	N/A	193 mV in 1.0 M KOH	9
$ZnPS_3$	890 mV < < 1200 mV in 0.5 M H_2SO_4	N/A	1
$ZnPSe_3$	~1300 mV in 0.5 M H_2SO_4	~2000 mV in 1.0 M KOH	7
$GaPS_4$	890 mV < < 1200 mV in 0.5 M H_2SO_4	N/A	1

$CdPS_3$	~1200 mV in 0.5 M H_2SO_4	N/A	1
$CdPSe_3$	~1300 mV in 0.5 M H_2SO_4	~2000 mV in 1.0 M KOH	7
$SnPS_3$	> 890 mV in 0.5 M H_2SO_4	N/A	1
$SnPSe_3$	~1200 mV in 0.5 M H_2SO_4	~2000 mV in 1.0 M KOH	7
$BiPS_4$	~400 mV in 0.5 M H_2SO_4	N/A	1

Supplementary Table 2. Hydrogen adsorption Gibbs energy values (ΔG_{H*}) on the different basal sites of $NiPS_3$ monolayer via the Volmer-Heyrovsky or Volmer-Tafel pathway considering the solvation effect in 17 vol% triethanolamine aqueous solution.

**basal sites**	**first hydrogen adsorption Gibbs energy values of Volmer step ΔG_{H*} (eV)**	**second hydrogen adsorption Gibbs energy values of Heyrovsky step ΔG_{H*} (eV)**	**second hydrogen adsorption Gibbs energy values of Tafel step ΔG_{H*} (eV)**
Ni	0.454	0.199	1.109
P	0.794	0.208	2.284
S	0.765	0.258	2.187

Supplementary Table 3. Hydrogen adsorption Gibbs energy values (ΔG_{H*}) on the different (100) edge sites of $NiPS_3$ monolayer via the Volmer-Heyrovsky or Volmer-Tafel pathway considering the solvation effect in 17 vol% triethanolamine aqueous solution.

**(100) edge sites**	**first hydrogen adsorption Gibbs energy values of Volmer step ΔG_{H*} (eV)**	**second hydrogen adsorption Gibbs energy values of Heyrovsky step ΔG_{H*} (eV)**	**second hydrogen adsorption Gibbs energy values of Tafel step ΔG_{H*} (eV)**
Ni	0.628	0.156	0.647
P	0.235	0.188	0.936
S1	0.401	0.244	0.978
S2	0.260	0.183	-0.129
S3	0.242	0.165	0.684

Supplementary Table 4. Hydrogen adsorption Gibbs energy values (ΔG_{H*}) on the different (010) edge sites of $NiPS_3$ monolayer via the Volmer-Heyrovsky or Volmer-Tafel pathway considering the solvation effect in 17 vol% triethanolamine aqueous solution.

**(010) edge sites**	**first hydrogen adsorption Gibbs energy values of Volmer step ΔG_{H*} (eV)**	**second hydrogen adsorption Gibbs energy values of Heyrovsky step ΔG_{H*} (eV)**	**second hydrogen adsorption Gibbs energy values of Tafel step ΔG_{H*} (eV)**
Ni	0.674	0.538	0.522
P	0.983	0.112	0.948
S	0.168	0.129	0.702

Supplementary Table 5. Hydrogen adsorption Gibbs energy values (ΔG_{H*}) on the different (1-30) edge Ni and P sites of $NiPS_3$ monolayer via the Volmer-Heyrovsky or Volmer-Tafel pathway considering the solvation effect in 17 vol% triethanolamine aqueous solution.

**(1-30) edge sites**	**first hydrogen adsorption Gibbs energy values of Volmer step ΔG_{H*} (eV)**	**second hydrogen adsorption Gibbs energy values of Heyrovsky step ΔG_{H*} (eV)**	**second hydrogen adsorption Gibbs energy values of Tafel step ΔG_{H*} (eV)**
Ni1	0.561	0.593	0.094
Ni2	0.379	0.077	0.364
P1	0.280	0.222	0.408
P2	0.355	0.051	0.857

Supplementary Table 6. Hydrogen adsorption Gibbs energy values (ΔG_{H^*}) on the different (1-30) edge S sites of $NiPS_3$ monolayer via the Volmer-Heyrovsky or Volmer-Tafel pathway considering the solvation effect in 17 vol% triethanolamine aqueous solution.

**(1-30) edge sites**	**first hydrogen adsorption Gibbs energy values of Volmer step ΔG_{H^*} (eV)**	**second hydrogen adsorption Gibbs energy values of Heyrovsky step ΔG_{H^*} (eV)**	**second hydrogen adsorption Gibbs energy values of Tafel step ΔG_{H^*} (eV)**
S1	0.428	0.163	0.680
S2	0.276	0.202	0.302
S3	0.115	0.601	0.182
S4	0.256	0.334	0.418
S5	0.181	0.513	1.004
S6	0.362	0.325	0.209
S7	0.122	0.584	0.742
S8	0.220	0.230	0.970
S9	0.349	0.964	0.836

Supplementary Table 7. Physicochemical properties of 0.0N and 20.0N.

**Samples**	**$NiPS_3$ (wt%) (ICP-AES)**	**S_{BET} (m^2 g^{-1})**	**PV [a] (cm^3 g^{-1})**	**APS [b] (nm)**
0.0N	0	53	0.28	21.2
20.0N	3.603	42	0.25	24.3

[a] PV: Pore volume. [b] APS: Average pore size.

Supplementary Table 8. Photocatalytic H_2-production rates for the representative noble-metal-free CdS-based heterostructured photocatalysts.

Photocatalysts	Amount of photocatalysts (mg)	Light sources	Sacrificial reagents	Photocatalytic H_2-production rates ($\mu mol\ h^{-1}\ g^{-1}$)	Quantum yield (%)	References
$NiPS_3/CdS$ (20.0N)	20	300 W Xe lamp (λ > 400 nm)	~17 vol% triethanolamine aqueous solution	13,600	20.2% at 420 nm	This work
CdS/In_2O_3	20	300 W Xe lamp (λ > 400 nm)	10 vol% triethanolamine aqueous solution	235.05	N/A	[10]
$Co_9S_8/Cd/CdS$	2	300 W Xe lamp (λ > 400 nm)	0.35 M Na_2S and 0.25 M Na_2SO_3 aqueous solution	5,210	N/A	[11]
CdS/MoC	40	300 W Xe lamp (λ > 420 nm)	10 vol% lactic acid aqueous solution	5,613	7.6% at 420 nm	[12]
VB_2/CdS	40	300 W Xe lamp (λ > 420 nm)	10 vol% lactic acid aqueous solution	12,100	4.4% at 420 nm	[13]
g-$C_3N_4/CdS/Ni_2P$	30	300 W Xe lamp (λ > 400 nm)	10 vol% triethanolamine aqueous solution	2,906	N/A	[14]
Polytriptycene@CdS	10	300 W Xe lamp (λ > 420 nm)	0.10 M Na_2S and 0.10 M Na_2SO_3 aqueous solution	9,480	N/A	[15]
Diethylenetriamine functionalized CdS/Cu_2S	20	300 W Xe lamp (λ > 420 nm)	0.35 M Na_2S and 0.25 M Na_2SO_3 aqueous solution	9,000	N/A	[16]
NiS/CdS	10	300 W Xe lamp	Na_2S and Na_2SO_3 aqueous solution	2,569	N/A	[17]
Bi_2MoO_6/CdS	5	150 W Xe lamp (λ > 420 nm)	10% lactic acid aqueous solution	6,830	5.9% at 420 nm	[18]

Nitrided $CoWO_4$/CdS	20	300 W Xe lamp (λ > 400 nm)	10 vol% lactic acid aqueous solution	3,650	N/A	[19]
CdS/MoO_x	30	300 W Xe lamp (λ > 400 nm)	10 vol% lactic acid aqueous solution	5,420	1.65% at 450 nm	[20]
CdS/NiS	100	300 W Xe lamp (λ > 400 nm)	~9.1 vol% lactic acid aqueous solution	542	N/A	[21]
MoS_2/CdS	80	300 W Xe lamp (λ > 420 nm)	10 vol% lactic acid aqueous solution	3,135	3.66% at 420 nm	[22]
CdS/Zn_2GeO_4	50	300 W Xe lamp (λ > 420 nm)	0.35 M Na_2S and 0.25 M Na_2SO_3 aqueous solution	1,720	N/A	[23]
$Ti_3C_2T_x$/CdS	20	300 W Xe lamp (λ > 420 nm)	10 vol% lactic acid aqueous solution	825	10.2% at 450 nm	[24]
Co_4S_3/CdS	50	300 W Xe lamp (λ > 420 nm)	10 vol% lactic acid aqueous solution	5,893	N/A	[25]
CdS/$CdWO_4$	20	300 W Xe lamp (λ > 420 nm)	10 vol% lactic acid aqueous solution	9,170	N/A	[26]
CdS/Ni-Fe LDH	50	300 W Xe lamp	10 vol% methanol aqueous solution	469	N/A	[27]
Co_2P/CdS	50	300 W Xe lamp (λ > 400 nm)	10 vol% lactic acid aqueous solution	6,060	13.88% at 420 nm	[28]
CdS/Cu_7S_4/C_3N_4	50	300 W Xe lamp (λ > 420 nm)	0.35 M Na_2S and 0.25 M Na_2SO_3 aqueous solution	3,570	4.4% at 420 nm	[29]
CdS/ZnSe	10	300 W Xe lamp (λ > 422 nm)	0.25 M Na_2S and 0.35 M Na_2SO_3 aqueous solution	119	14.8% at 420 nm	[30]
CdS/NiCo-LDH	50	300 W Xe lamp (λ > 400 nm)	10 vol% lactic acid aqueous solution	8,665	14% at 420 nm	[31]

CdS/MoS_2	200	300 W Xe lamp ($\lambda > 420$ nm)	10 vol% lactic acid aqueous solution	49,800	41.37% at 420 nm	[32]
$WO_3/CdS/WS_2$	30	300 W Xe lamp ($\lambda > 420$ nm)	16.67 vol% lactic acid aqueous solution	14,340	22.96% at 435 nm	[33]

Table 9. Photocatalytic H_2-production rates for the reported MPC_x based photocatalysts.

Photocatalysts	Amount of photocatalysts (mg)	Light sources	Sacrificial reagents	Photocatalytic H_2-production rates (μmol h^{-1} g^{-1})	Quantum yield (%)	References
$NiPS_3$/CdS (20.0N)	20	300 W Xe lamp (λ > 400 nm)	~17 vol% triethanolamine aqueous solution	13,600	20.2% at 420 nm	This work
$NiPS_3$ nanosheets covered carbon fiber	~10-15	300 W Xe lamp	None sacrificial reagents (in pure water)	~26.4	N/A	[34]
$NiPS_3$ nanosheets covered carbon fiber	~10-15	300 W Xe lamp	Na_2S & Na_2SO_3 aqueous solution	74.67	N/A	[34]
Eosin Y sensitized $NiPS_3$ sheets	N/A	400 W Xe lamp	15 vol% triethanolamine aqueous solution	2,600	N/A	[35]
$FePS_3$ Quantum sheets	5	300 W Xe lamp	10 vol% triethanolamine aqueous solution	290	N/A	[36]
$FePS_3$ nanosheets	N/A	AM 1.5 G solar simulator	0.1 M Na_2S and Na_2SO_3 aqueous solution	402.4	0.94% at 350 nm	[37]
porous $FePS_3$ nanosheets	10	300 W Xe lamp	10 vol% triethylamine aqueous solution	305.6	N/A	[38]
$MnPS_3$ nanosheets covered carbon fiber	N/A	AM 1.5 G solar simulator	0.35 M Na_2S & 0.25 M Na_2SO_3 aqueous solution	21.2	N/A	[39]
$MnPSe_3$ nanosheets covered carbon fiber	~20	AM 1.5 G solar simulator	0.35 M Na_2S & 0.25 M Na_2SO_3 aqueous solution	43.5	N/A	[39]

Supplementary Table 10. The fitting results of decay kinetics for 0.0N and 20.0N samples.

Samples	λ (nm)	A_1 (%)	τ_1 (ps)	A_2 (%)	τ_2 (ps)	A_3 (%)	τ_3 (ps)
0.0N	~516	1.634	40.47 ± 2.31	27.866	610.67 ± 33.57	70.500	2677.93 ± 187.45
20.0N	~514	1.131	94.80 ± 3.88	13.467	1128.62 ± 59.43	85.402	8458.55 ± 903.92
0.0N	~480	0.464	37.75 ± 3.68	20.375	627.27 ± 22.13	79.161	7918.81 ± 1169.49
20.0N	~474	0.438	42.40 ± 4.13	19.792	724.99 ± 26.44	79.769	9484.35 ± 1762.90

Supplementary Table 11. The fitting results of decay kinetics for 0.0N and 20.0N samples in the long-time transient absorption spectroscopy (up to 7.73 ns).

Samples	λ (nm)	A_1 (%)	τ_1 (ps)	A_2 (%)	τ_2 (ps)	A_3 (%)	τ_3 (ps)
0.0N	~502	0.692	11.11 ± 0.76	7.524	203.03 ± 20.27	91.784	5482.73 ± 1322.77
20.0N	~501	0.952	28.18 ± 1.73	11.447	762.60 ± 114.61	87.601	8488.71 ± 2451.34

Supplementary Table 12. Hydrogen adsorption Gibbs energy values (ΔG_{H^*}) on the Ni, P and S sites of $NiPS_3$ basal plane in $NiPS_3$/CdS via the Volmer-Heyrovsky or Volmer-Tafel pathway considering the solvation effect in 17 vol% triethanolamine aqueous solution.

basal sites	first hydrogen adsorption Gibbs energy values of Volmer step ΔG_{H^*} (eV)	second hydrogen adsorption Gibbs energy values of Heyrovsky step ΔG_{H^*} (eV)	second hydrogen adsorption Gibbs energy values of Tafel step ΔG_{H^*} (eV)
Ni	0.635	0.109	1.153
P	0.493	0.136	1.354
S	0.335	0.074	1.127

Supplementary Table 13. Atomic coordinates of (002) facet for $NiPS_3$ monolayer.

direct	x	y	z
Ni1	0.999995210	0.333207759	0.081760529
Ni2	0.499995210	0.833207759	0.081760529
Ni3	0.999995210	0.666792241	0.081760529
Ni4	0.499995210	0.166792241	0.081760529
P1	0.000315627	0.000000000	0.107092564
P2	0.500315627	0.500000000	0.107092564
P3	0.999813776	0.000000000	0.056410781
P4	0.499813776	0.500000000	0.056410781
S1	0.173028197	0.172708091	0.117042860
S2	0.673028197	0.672708091	0.117042860
S3	0.826964999	0.172711936	0.046466920
S4	0.326964999	0.672711936	0.046466920
S5	0.826964999	0.827288064	0.046466920
S6	0.326964999	0.327288064	0.046466920
S7	0.173028197	0.827291909	0.117042860
S8	0.673028197	0.327291909	0.117042860
S9	0.154942707	0.500000000	0.117061620
S10	0.654942707	0.000000000	0.117061620
S11	0.844951077	0.500000000	0.046474415
S12	0.344951077	0.000000000	0.046474415

Supplementary Table 14. Atomic coordinates of (100) edge for $NiPS_3$ monolayer.

direct	x	y	z
Ni1	0.334386398	0.123522703	0.081830039
Ni2	0.819220054	0.041345184	0.077728030
Ni3	0.665627073	0.123511932	0.081827216
Ni4	0.180730177	0.041354370	0.077711800
Ni5	0.319354609	0.292074053	0.085779576
Ni6	0.834343732	0.209804567	0.081736721
Ni7	0.680698871	0.292071514	0.085766684
Ni8	0.165692841	0.209804135	0.081733954
P1	0.000065561	0.123493105	0.111886175
P2	0.499960250	0.036810575	0.110461181
P3	0.000007602	0.124863737	0.060225597
P4	0.499993672	0.038652379	0.059746779
P5	0.000087603	0.294643107	0.103895886
P6	0.500030968	0.208490191	0.103208274
P7	0.000016693	0.296564188	0.053126644
P8	0.500033931	0.209811547	0.051477682
S1	0.173298267	0.157250158	0.114082387
S2	0.678714740	0.070363687	0.112857818
S3	0.167319325	0.097620223	0.046170287
S4	0.670666361	0.014437633	0.044646095
S5	0.832736822	0.097628926	0.046156529
S6	0.329341023	0.014432283	0.044645756
S7	0.826705549	0.157214443	0.114099402
S8	0.321272947	0.070363188	0.112858665
S9	0.500009329	0.150305828	0.114432948

S10	0.999950362	0.037652718	0.107006946
S11	0.500025441	0.099059053	0.050389805
S12	0.000012962	0.004050041	0.068718768
S13	0.170873172	0.318883359	0.119007497
S14	0.667015197	0.235747378	0.117356290
S15	0.178619700	0.262916622	0.050735865
S16	0.673125038	0.176054953	0.049356232
S17	0.821413107	0.262943837	0.050715783
S18	0.326543205	0.176114078	0.049403469
S19	0.829204895	0.318801285	0.119032614
S20	0.332827857	0.235721443	0.117316218
S21	0.500007006	0.329415571	0.094638228
S22	0.000208529	0.234214343	0.113191678
S23	0.499995457	0.295667953	0.056385470
S24	0.000020721	0.182990710	0.048973017

Supplementary Table 15. Atomic coordinates of (010) edge for $NiPS_3$ monolayer.

direct	x	y	z
Ni1	0.999536434	0.140373744	0.918107451
Ni2	0.500109468	0.281625360	0.918244398
Ni3	0.000109497	0.051707805	0.918244345
Ni4	0.499536359	0.192959854	0.918107498
P1	0.976218054	0.250449623	0.892999491
P2	0.476216618	0.082883554	0.892999406
P3	0.023673615	0.250460714	0.943559128
P4	0.523674415	0.082872513	0.943559197
S1	0.817254257	0.190619390	0.884243226
S2	0.269480714	0.031144402	0.884340336
S3	0.182638920	0.190526013	0.952145031
S4	0.730767900	0.031157213	0.952190328
S5	0.230766253	0.302176125	0.952190477
S6	0.682641130	0.142807220	0.952145076
S7	0.769481683	0.302189152	0.884340128
S8	0.317252208	0.142713819	0.884243195
S9	0.810109264	0.077572450	0.877894949
S10	0.310107638	0.255760801	0.877894576
S11	0.190212338	0.077407331	0.958695690
S12	0.690213235	0.255925919	0.958696074

Supplementary Table 16. Atomic coordinates of (1-30) edge for $NiPS_3$ monolayer.

direct	x	y	z
Ni1	0.408693735	0.221867532	0.144185294
Ni2	0.467315179	0.895867250	0.090610422
Ni3	0.440850885	0.527818517	0.054718545
Ni4	0.442745300	0.433914461	0.129871140
Ni5	0.484624097	0.111699552	0.071245488
Ni6	0.460496810	0.788782492	0.035959125
Ni7	0.307908844	0.775890186	0.175464184
Ni8	0.250124901	0.102453856	0.229133638
Ni9	0.277160171	0.469922539	0.265313325
Ni10	0.275943291	0.563790505	0.190090982
Ni11	0.233431771	0.886503981	0.248581831
Ni12	0.258622020	0.208896010	0.284075259
P1	0.366531024	0.331944060	0.042903751
P2	0.205324563	0.997886891	0.147046311
P3	0.161768869	0.661147493	0.087601196
P4	0.675148835	0.331269646	0.064542330
P5	0.608414379	0.001220151	0.005591386
P6	0.482264838	0.662711917	0.109391312
P7	0.351231447	0.666209271	0.276857166
P8	0.512088412	0.000306717	0.172746315
P9	0.555934843	0.336412210	0.232456566
P10	0.042563993	0.666915632	0.255262294
P11	0.109807943	0.997288212	0.314258011
P12	0.235361517	0.335207932	0.210622760
S1	0.297218465	0.221290146	0.021753053

S2	0.182718904	0.880121552	0.124360083
S3	0.135028389	0.551880284	0.061353799
S4	0.658722199	0.212759927	0.108061062
S5	0.693930178	0.880353672	0.048819591
S6	0.544929197	0.555365898	0.156477100
S7	0.861846144	0.334608313	0.024740097
S8	0.663381442	0.001643964	0.123279875
S9	0.608256014	0.656504595	0.056756817
S10	0.266932647	0.330787990	0.102430515
S11	0.358443669	0.001477312	0.040693239
S12	0.030647564	0.655402918	0.138672664
S13	0.323223857	0.448968183	0.006734630
S14	0.185097889	0.105536908	0.102855604
S15	0.144928464	0.769764611	0.042500728
S16	0.682654522	0.450183084	0.088761015
S17	0.727752860	0.107399919	0.031152980
S18	0.559188605	0.774236167	0.136714313
S19	0.419576908	0.777031694	0.298003932
S20	0.534332708	0.118181604	0.195360350
S21	0.582909031	0.445783918	0.258702304
S22	0.058444610	0.785335831	0.211741166
S23	0.024203833	0.118071379	0.271032357
S24	0.173722909	0.442521316	0.163452168
S25	0.856239518	0.663630637	0.295143428
S26	0.054861557	0.996444438	0.196682316
S27	0.109693481	0.341206543	0.263344985
S28	0.450896365	0.667385835	0.217354924

S29	0.359302188	0.996960325	0.279076432
S30	0.686997643	0.342288191	0.181372425
S31	0.395624454	0.549263084	0.313020246
S32	0.532545924	0.892783285	0.216982232
S33	0.573989347	0.227986137	0.277643627
S34	0.035666482	0.547750148	0.231129357
S35	0.990472649	0.891087814	0.288692641
S36	0.158021726	0.224075737	0.183059263

Supplementary Table 17. Atomic coordinates of $NiPS_3$ (002) facet/CdS (200) facet heterostructure.

direct	x	y	z
Cd1	0.080459997	0.354660004	0.100369997
Cd2	0.061918930	0.370452882	0.208892436
Cd3	0.580460012	0.104659997	0.100369997
Cd4	0.569949617	0.128983725	0.218220677
Cd5	0.080459997	0.104659997	0.045100000
Cd6	0.079842605	0.113524826	0.156420906
Cd7	0.580460012	0.354660004	0.045100000
Cd8	0.581986363	0.348415884	0.155657237
Cd9	0.080459997	0.854659975	0.100369997
Cd10	0.071206481	0.871650551	0.204192110
Cd11	0.580460012	0.604659975	0.100369997
Cd12	0.572174422	0.623155572	0.203763580
Cd13	0.080459997	0.604659975	0.045100000
Cd14	0.079097512	0.604439083	0.151912082
Cd15	0.580460012	0.854659975	0.045100000

Cd16	0.566035299	0.874877311	0.153597723
S1	0.330460012	0.229660004	0.017470000
S2	0.329701957	0.228909752	0.128673292
S3	0.830460012	0.479660004	0.017470000
S4	0.821368127	0.474392035	0.128191895
S5	0.330460012	0.479660004	0.072729997
S6	0.319943378	0.482819163	0.180531487
S7	0.830460012	0.229660004	0.072729997
S8	0.819306850	0.226895000	0.185633662
S9	0.330460012	0.729659975	0.017470000
S10	0.336508285	0.736105922	0.128092787
S11	0.830460012	0.979659975	0.017470000
S12	0.842084982	0.988323430	0.128651621
S13	0.330460012	0.979659975	0.072729997
S14	0.356439525	0.001142410	0.185443841
S15	0.830460012	0.729659975	0.072729997
S16	0.831890628	0.740667957	0.178673436
S17	0.172772565	0.141072775	0.308296637
S18	0.914739541	0.170010293	0.250348975
S19	0.716729640	0.632909028	0.301488044
S20	0.348354934	0.641915002	0.242740284
S21	0.167752793	0.826614912	0.311733240
S22	0.893422014	0.857113192	0.243330653
S23	0.695989379	0.310664743	0.299407816
S24	0.375568233	0.323419090	0.240322231
S25	0.207218939	0.470218704	0.298133573
S26	0.858253741	0.522029365	0.236585007

S27	0.669177262	0.967669732	0.305320965
S28	0.404572730	0.000365435	0.254329072
Ni1	0.053150362	0.291874904	0.284797503
Ni2	0.491891710	0.788306886	0.297643446
Ni3	0.086139215	0.657583311	0.295872018
Ni4	0.498131802	0.137219595	0.287566410
P1	0.010252507	0.981337817	0.296772350
P2	0.057456539	0.003403149	0.257589584
P3	0.550362767	0.473198442	0.288610904
P4	0.533810626	0.487131264	0.248853691

Supplementary Table 18. Atomic coordinates of CdS (200) facet.

**direct**	**x**	**y**	**z**
Cd1	0.080459997	0.354660004	0.107390001
Cd2	0.080521760	0.354729417	0.225900606
Cd3	0.580460012	0.104659997	0.107390001
Cd4	0.580408740	0.104646322	0.225890022
Cd5	0.080459997	0.104659997	0.049059998
Cd6	0.080416386	0.104647588	0.165827175
Cd7	0.580460012	0.354660004	0.049059998
Cd8	0.580651086	0.354737798	0.165815492
Cd9	0.080459997	0.854659975	0.107390001
Cd10	0.080521547	0.854729634	0.225900155
Cd11	0.580460012	0.604659975	0.107390001

Cd12	0.580408695	0.604645728	0.225889882
Cd13	0.080459997	0.604659975	0.049059998
Cd14	0.080416884	0.604647322	0.165827164
Cd15	0.580460012	0.854659975	0.049059998
Cd16	0.580650944	0.854737809	0.165815445
S1	0.330460012	0.229660004	0.019900000
S2	0.330457601	0.229629107	0.138048433
S3	0.830460012	0.479660004	0.019900000
S4	0.830177709	0.479611403	0.138035047
S5	0.330460012	0.479660004	0.078220002
S6	0.330421183	0.479659857	0.197544793
S7	0.830460012	0.229660004	0.078220002
S8	0.830517525	0.229656506	0.197549711
S9	0.330460012	0.729659975	0.019900000
S10	0.330457647	0.729629171	0.138048446
S11	0.830460012	0.979659975	0.019900000
S12	0.830177625	0.979611349	0.138035053
S13	0.330460012	0.979659975	0.078220002
S14	0.330420586	0.979659346	0.197544850
S15	0.830460012	0.729659975	0.078220002
S16	0.830517876	0.729656751	0.197549879

Supplementary References

[1] Mayorga-Martinez, C. C. et al. Layered metal thiophosphite materials: magnetic, electrochemical and electronic properties. *ACS Appl. Mater. Interfaces* **9**, 12563-12573 (2017).

[2] Gusmão, R., Sofer, Z. & Pumera, M. Exfoliated layered manganese trichalcogenide phosphite ($MnPX_3$, X = S, Se) as electrocatalytic van der Waals materials for hydrogen evolution. *Adv. Funct. Mater.* **29**, 1805975 (2019).

[3] Rakov, D., Li, Y., Niu, S. & Xu, P. Insight into Mn and Ni doping of $Ni_{1-x}Mn_xPS_3$ and $Mn_{1-x}Ni_xPS_3$ nanosheets on electrocatalytic hydrogen and oxygen evolution activity. *J. Alloys Compd.* **769**, 532-538 (2018).

[4] Mukherjee, D., Austeria, P. M. & Sampath, S. Two-dimensional, few-layer phosphochalcogenide, $FePS_3$: a new catalyst for electrochemical hydrogen evolution over wide pH range. *ACS Energy Lett.* **1**, 367-372 (2016).

[5] Yu, Z. et al. Amine-assisted exfoliation and electrical conductivity modulation toward few-layer $FePS_3$ nanosheets for efficient hydrogen evolution. *J. Mater. Chem. A* **7**, 13928-13934 (2019).

[6] Wang, S. et al. Cobalt doping of $FePS_3$ promotes intrinsic active sites for the efficient hydrogen evolution reaction. *Nanoscale* **12**, 14459-14464 (2020).

[7] Gusmao, R., Sofer, Z., Sedmidubsky, D., Huber, Š. & Pumera M. The role of the metal element in layered metal phosphorus triselenides upon their electrochemical sensing and energy applications. *ACS Catal.* **7**, 8159-8170 (2017).

[8] Sanna, M., Ng, S. & Pumera, M. Layered transition metal selenophosphites for visible light photoelectrochemical production of hydrogen. *Electrochem Commun.* **129**, 107077 (2021).

[9] Song, B. et al. Tuning mixed nickel iron phosphosulfide nanosheet electrocatalysts for enhanced hydrogen and oxygen evolution. *ACS Catal.* **7**, 8549-8557 (2017).

[10] Ren, J.-T., Yuan, K., Wu, K., Zhou, L. & Zhang, Y.-W. A robust CdS/In_2O_3 hierarchical heterostructure derived from a metal-organic framework for efficient visible-light photocatalytic hydrogen production. *Inorg. Chem. Front.* **6**, 366-375 (2019).

[11] Zhang, T. et al. Z-scheme transition metal bridge of Co_9S_8/Cd/CdS tubular heterostructure for enhanced photocatalytic hydrogen evolution. *Appl. Catal. B-Environ.* **286**, 119853 (2021).

[12] Lei, Y. et al. Noble-metal-free metallic MoC combined with CdS for enhanced visible-light-driven photocatalytic hydrogen evolution. *J. Clean. prod.* **322**, 129018 (2021).

[13] Tian, L., Wang, F., Zhang, Z. & Min, S. Vanadium diboride as an efficient cocatalyst coupled with CdS for enhanced visible light photocatalytic H_2 evolution. *Int. J. Hydrog. Energy* **45**, 19017-19026 (2020).

[14] Gong, Q. et al. Mesoporous g-C_3N_4 decorated by Ni_2P nanoparticles and CdS nanorods together for enhancing photocatalytic hydrogen evolution. *Int. J. Hydrog. Energy* **46**, 21442-21453 (2021).

[15] Liang, Q. et al. Polytriptycene@CdS double shell hollow spheres with enhanced interfacial charge transfer for highly efficient photocatalytic hydrogen evolution. *J. Mater. Chem. A* **9**, 9105-9112 (2021).

[16] Li, X., Dai, K., Pan, C. & Zhang, J. Diethylenetriamine-functionalized CdS nanoparticles decorated on Cu_2S snowflake microparticles for photocatalytic hydrogen production. *ACS Appl. Nano Mater.* **3**, 11517-11526 (2020).

[17] Tan, W., Li, Y., Jiang, W., Gao, C. & Zhuang, C. CdS nanospheres decorated with NiS quantum dots as nobel metal-free photocatalysts for efficient hydrogen evolution. *ACS Appl. Energy Mater.* **3**, 8048-8054 (2020).

[18] Chava, R. K., Son, N., Kim, Y. S. & Kang, M. Integration of perovskite type Bi_2MoO_6 nanosheets onto one dimensional CdS: a type-II heterostructured photocatalytic system for efficient charge separation in the hydrogen evolution reaction. *Inorg. Chem. Front.* **7**, 2818-2832 (2020).

[19] Kuang, W. et al. Nitridation of $CoWO_4$/CdS nanocomposite formed metal nitrides assisting efficiently photocatalytic hydrogen evolution. *ACS Omega* **5**, 9969-9976 (2020).

[20] Jiang, C. et al. Promoting photocatalytic hydrogen production by a core-shell CdS@MoO_x photocatalyst connected by an S-Mo “bridge”. *Catal. Sci. Technol.* **10**, 1368-1375 (2020).

[21] Yang, Y. et al. Photocatalytic performance of NiS/CdS composite with multistage structure. *ACS Appl. Energy Mater.* **3**, 7736-7745 (2020).

[22] Zhang, Z.-W., Li, Q.-H., Qiao, X.-Q., Hou, D. & Li D.-S., One-pot hydrothermal synthesis of willow branch-shaped MoS_2/CdS heterojunctions for photocatalytic H_2 production under visible light irradiation. *Chinese J. Catal.* **40**, 371-379 (2019).

[23] Hou, Z. et al. Fabrication of CdS/Zn_2GeO_4 heterojunction with enhanced visible-light photocatalytic H_2 evolution activity. *Int. J. Hydrog. Energy* **44**, 28649-28655 (2019).

[24] Yang, Y., Zhang, D. & Xiang, Q. Plasma-modified $Ti_3C_2T_x$/CdS hybrids with oxygen-containing groups for high-efficiency photocatalytic hydrogen production. *Nanoscale* **11**, 18797-18805 (2019).

[25] Huang, R. et al. Two dimensional metal-organic frameworks-derived leaf-like Co_4S_3/CdS composite for enhancing photocatalytic water evolution. *J. Colloid Interface Sci.* **554**, 39-47 (2019).

[26] Jia, X. et al. Direct Z-scheme composite of CdS and oxygen-defected $CdWO_4$: An efficient visible-light-driven photocatalyst for hydrogen evolution. *Appl. Catal. B-Environ.* **198**, 154-161 (2016).

[27] Zhou, H. et al. Fabrication of CdS/Ni-Fe LDH heterostructure for improved photocatalytic hydrogen evolution from aqueous methanol solution. *Int. J. Hydrog. Energy* **43**, 14328-14366 (2018).

[28] Li, S. et. al. In situ synthesis of strongly coupled Co_2P-CdS nanohybrids: an effective strategy to regulate photocatalytic hydrogen evolution activity. *ACS Sustainable Chem. Eng.* **6**, 9940-9950 (2018).

[29] Chu, J. et. al. Highly efficient visible-light-driven photocatalytic hydrogen production on CdS/Cu_7S_4/g-C_3N_4 ternary heterostructures. *ACS Appl. Mater. Interfaces* **10**, 20404-20411 (2018).

[30] Durian-shaped CdS@ZnSe core@mesoporous-shell nanoparticles for enhanced and sustainable photocatalytic hydrogen evolution. *J. Phys. Chem. Lett.* **9**, 2212-2217 (2018).

[31] Li, S. et al. Novel photocatalyst incorporating Ni-Co layered double hydroxides with P doped CdS for enhancing photocatalytic activity towards hydrogen evolution. *Appl. Catal. B-Environ.* **254**, 145-155 (2019).

[32] Yin, X.-L. et al. MoS_2/CdS Nanosheets-on-nanorod heterostructure for highly efficient photocatalytic H_2 generation under visible light irradiation. *ACS Appl. Mater. Interfaces* **8**, 15258-15266 (2016).

[33] Xue, C., Zhang, P., Shao, G. & Yang, G. Effective promotion of spacial charge separation in direct Z-scheme WO_3/CdS/WS_2 tandem heterojunction with enhanced visible-light-driven photocatalytic H_2 evolution. *Chem. Eng. J.* **398**, 125602 (2020).

[34] Wang, F. et al. Two-dimensional metal phosphorus trisulfide nanosheet with solar hydrogen-evolving activity. *Nano Energy* **40**, 673-680 (2017).

[35] Barua, M., Ayyub, M., Vishnoi, P., Pramoda, K. & Rao, C. N. R. et al. Photochemical HER activity of layered metal phospho-sulfides and -selenides. *J. Mater. Chem. A* **7**, 22500-22506 (2019).

[36] Cheng, Z. et al. High-yield production of monolayer $FePS_3$ quantum sheets via chemical exfoliation for efficient photocatalytic hydrogen evolution. *Adv. Mater.* **30**, 1707433 (2018).

[37] Cheng, Z., Sendeku, M. G. & Liu, Q. Layered metal phosphorous trichalcogenides nanosheets: facile synthesis and photocatalytic hydrogen evolution. *Nanotechnology* **31**, 135405 (2020).

[38] Zhang, J. et al. Tailoring the porosity in iron phosphosulfide nanosheets to improve the performance of photocatalytic hydrogen evolution. *ChemSusChem* **12**, 2651-2659 (2019).

[39] Shifa, T. et al. High crystal quality 2D manganese phosphorus trichalcogenide nanosheets and their photocatalytic activity. *Adv. Funct. Mater.* **28**, 1800548 (2018).
